# A Bifunctional Small Molecule Degrader of the Long Noncoding RNA MALAT1 Triplex

**DOI:** 10.1002/chem.202600025

**Published:** 2026-02-05

**Authors:** Christian A. T. Brega, Benjamin A. Craig, Sigitas Mikutis, Rupert S. J. Proctor, Yuliia Vyborna, Galway Ivey, Maria Eleftheriou, Kostas Tzelepis, Mo Yang, Shaifaly Parmar, Javier Bonet‐Aleta, John S. Schneekloth, Gonçalo J. L. Bernardes

**Affiliations:** ^1^ Yusuf Hamied Department of Chemistry University of Cambridge Cambridge UK; ^2^ Cambridge Stem Cell Institute University of Cambridge Cambridge UK; ^3^ Department of Haematology University of Cambridge Cambridge UK; ^4^ Milner Therapeutics Institute University of Cambridge Cambridge UK; ^5^ Experimental Cancer Genetics Wellcome Trust Sanger Institute Cambridge UK; ^6^ Department Chemical Biology Laboratory Institution Center for Cancer Research National Cancer Institute Frederick Maryland USA; ^7^ Translational Chemical Biology Group Spanish National Cancer Research Centre (CNIO) Madrid Spain

**Keywords:** degraders, MALAT1, proximity induced, RNA, small‐molecule

## Abstract

The targeted degradation of RNA, particularly long noncoding RNAs (lncRNAs), holds immense potential for therapeutic interventions in diseases associated with aberrant RNA regulation. Here, we introduce a novel Proximity–Induced Nucleic Acid Degrader (**PINAD–1)**, a first‐in‐class small molecule that selectively induces the degradation of MALAT1, a lncRNA implicated in the regulation of metastatic processes. **PINAD–1** is designed by conjugating a binder specific for the triple helix structure of MALAT1 to an imidazole‐based RNA‐degrading warhead, enabling specific cleavage of the MALAT1 transcript in vitro and in cellulo, with minimal off‐target effects on the structurally similar NEAT1 lncRNA. Through mechanistic studies, we show that effective RNA degradation is not solely dependent on proximity but requires a precise structural context, as demonstrated by the differential activity of **PINAD–1** compared to the structurally analogous but functionally inert conjugate **PINAD–2**. Our findings underscore the importance of binder‐induced destabilization and RNA geometry in facilitating RNA degradation. This work lays the foundation for the design of bifunctional small‐molecule RNA degraders as powerful tools for the modulation of structured noncoding RNAs, offering potential applications in RNA‐based therapeutics.

## Graphical abstract

Targeting disease‐linked RNAs with small molecules is often limited by the modest impact of binding RNA structure alone. Here, we present a first‐in‐class bifunctional molecule that degrades the oncogenic lncRNA MALAT‐1 by inducing proximity between the RNA and a cleaving warhead. The approach presented herein underscores the relevance of converting a binder into a degrader for generating biological activity beyond simple RNA binding.

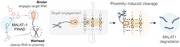

## 1 Introduction

**FIGURE 1:**
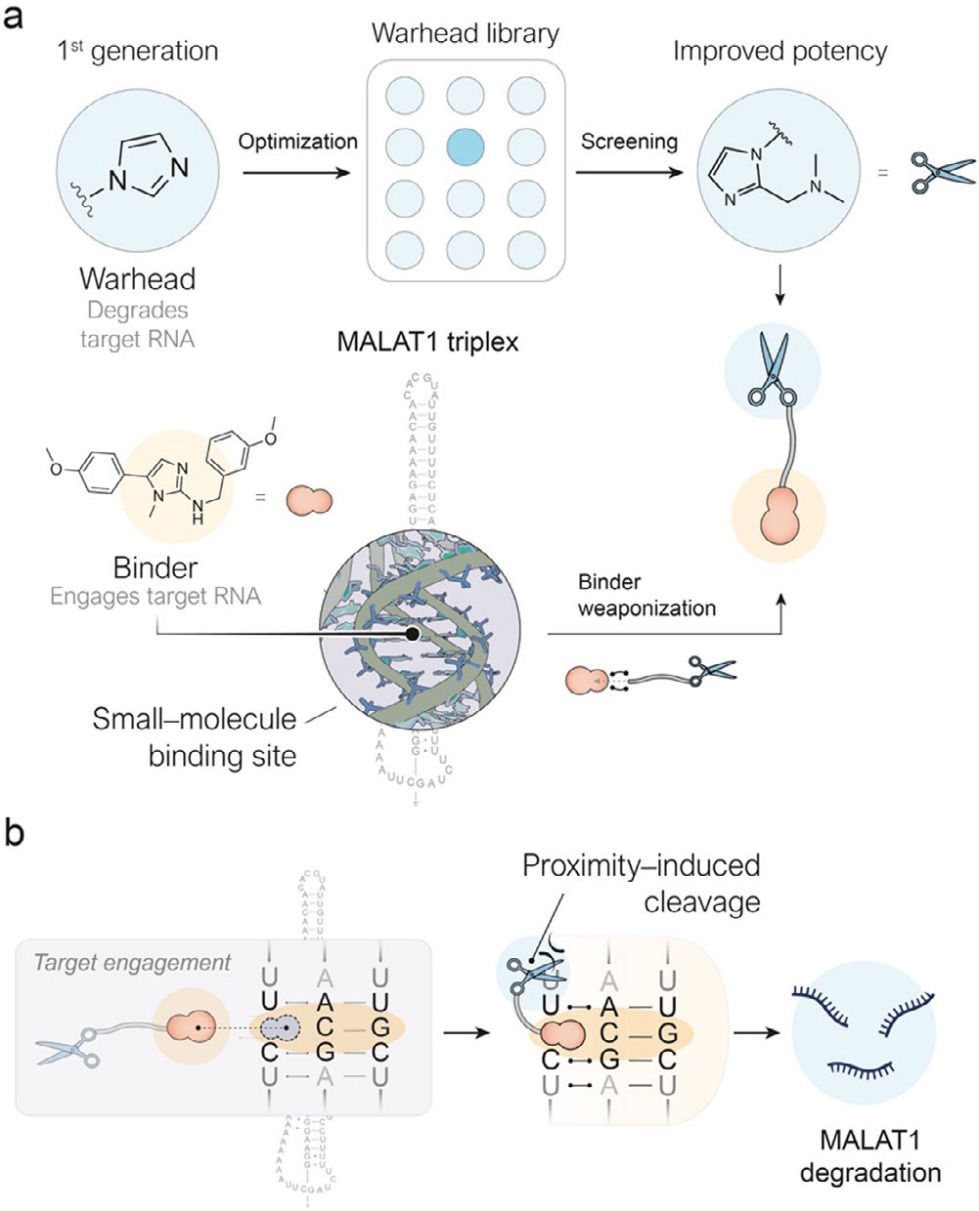
(a) Design strategy to develop a potent MALAT1 degrader by weaponizing a small‐molecule binder—known to engage the MALAT1 triplex—using an optimized warhead. (b) Mechanism of action of the bifunctional molecule in MALAT1 degradation. In the initial step, the binder interacts with a triplex motif present in the MALAT1 transcript, bringing the RNA into proximity with the warhead, which cleaves the nucleotide structure and leads to MALAT1 degradation.

Targeting RNA with small molecules remains an area with significant potential for the development of novel therapeutics, largely due to the challenges in discovering small‐molecule binders and their often‐limited ability to impact cellular phenotypes [1]. Transforming small‐molecule RNA binders into active degraders can be achieved by “weaponizing” the binder: linking it to a warhead to generate a bifunctional molecule capable of degrading the target RNA through a proximity‐induced mechanism (Figure 1a). Examples of types of RNAs targeted with small‐molecule RNA degraders include mRNAs [1, 2], pre‐miRs [3, 4], or viral RNA [5]. To expand the scope of druggable RNAs, we decided to focus on a novel target class: long noncoding RNAs (lncRNAs), which have recently gained increased attention due to emerging studies demonstrating their role as functional molecules in the cellular environment, regulating several processes related to gene expression [6], such as chromatin remodeling [7] and epigenetic modifications [8]. Among these transcripts, Metastasis‐Associated Lung Adenocarcinoma Transcript 1 (MALAT1) [9] has emerged as a prominent oncogenic lncRNA, owing to its consistent overexpression in cancer tissues and its established role in promoting tumorigenesis. Notably, MALAT1 knockdown has been shown to reduce the incidence of secondary neoplasms [10, 11, 12]. Within the 8000 kb that make up the long noncoding sequence of MALAT1, a ∼1500 nt segment located near the 3’ end (nts 6918–8441), has been specifically identified as critical for its pro‐metastatic activity [13]. Of particular interest is the highly conserved 3′‐end sequence of MALAT1 (nts 8254–8413), which possesses a unique bipartite triple helix secondary structure [14]. This structural motif is of exceptional interest for therapeutic targeting as its distinctive architecture offers opportunities for selective molecular recognition. Notably, uncommon architectures at the 3’ end of RNA transcripts are known to have a stabilizing effect against exonucleolytic decay in RNA transcripts lacking a 3’ polyA tail [15], thus promoting nuclear speckle retention [16] followed by severe overexpression, which can trigger the metastatic cascade. Over the past two decades, multiple studies have reported that disruption of this triplex motif is tightly correlated to MALAT1 level decrease [14] and impaired cell proliferation and migration [10, 17, 18]. As a result, the MALAT1 triplex has been deemed a target of primary importance in the oncogenic community.

## 2 Results and Discussion

### 2.1 Discovery of an Optimized Warhead for RNA Degradation

**FIGURE 2:**
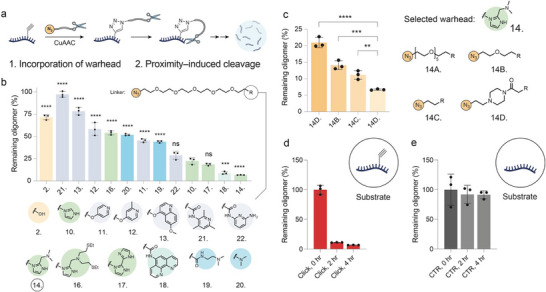
(a) Schematic representation of the methodology used to assess the activity of novel RNA click degraders. The warhead–linker–N_3_ is covalently attached to a model oligonucleotide containing a propargyl moiety at one of its 6A positions via CuAAC chemistry. The resulting oligonucleotide–warhead conjugate is then incubated at 37 °C under click assay conditions, and the remaining oligonucleotide is quantified by LC–MS. (b) Quantification of the remaining oligomer for every warhead tested. Confidence intervals are shown for each warhead against compound 10 – the first‐generation imidazole warhead. (c) Screen of linkers for degrader 14. (d) Change in concentration of RNA‐degrader 14 conjugate at 0, 2, 4 h timepoints. (e) Control unlabeled RNA sequence incubated under the same conditions at 0, 2, 4 h timepoints. ns = not significant, *p < 0.05, **p < 0.01, ***p < 0.001.

The considerable scientific interest in this target, combined with the recent identification of a limited pool of small molecules that bind MALAT1 with high specificity [17, 19], prompted our group to investigate the synthesis of a small‐molecule RNA degrader designed to target MALAT1 (Figure 1b). This strategy involves the covalent linkage of a MALAT1‐binding small molecule to a warhead developed in our laboratory in an effort to produce a second generation of RNA‐degrading moieties that mimic the active site of ribonucleases (Figure 1a). Previously, our group presented promising results in the field of RNA degradation using an imidazole modification, both through covalent attachment to target transcripts, as in the MeClickSeq case [20], and in a noncovalent manner through the use of bifunctional small molecule RNA–degrader, termed Proximity‐Induced Nucleic Acid Degraders (PINADs) [5]. Whilst imidazole alone has been sufficient for these previous studies, a high activity is particularly important in a therapeutic setting to maximize phenotypic effect at low concentrations. Therefore, we sought to weaponize a MALAT1 binder using a more potent warhead (Figure 1a). In the previous works published by our group, we adopted a click‐liquid chromatography mass spectroscopy (LC‐MS)‐based method for quantifying degrader‐mediated RNA cleavage in vitro, which was shown to be predictive of relative activity in‐cellulo [5, 20]. Screening of a range of functionally similar warhead moieties was therefore carried out using this platform (Figure 2a). Imidazole, pyridine, and diamine functionalities were chosen due to their perceived ability to act through general acid/base or metal‐bound‐based mechanisms of RNA cleavage. As before, click degraders were synthesized as warhead—linker—azide; comparative activity was determined using CuAAC attachment of the degraders to a synthetic 11‐mer functionalized with a propargyl moiety on one of its ^6^A positions, followed by incubation under click conditions at 37°C (Figure 2b). Mass spectroscopy (MS) was used to quantify the intact RNA‐degrader conjugate (Figure 2b and Figure S1). Most degraders displayed moderate cutting ability, with four novel moieties with the same or better activity than the original imidazole warhead. Degrader 18 represents a positive control here, since phenanthroline is well reported in the literature for use in the cleavage of DNA or RNA in the presence of Cu or Zn ions. However, its ability to intercalate duplexes can lead to nonselective damage of nucleic acids and cytotoxic effects, limiting its potential for therapeutic development [21]. For this reason, we decided to use the optimal degrader (14) to optimize the linker (i.e., the spacer between binder and warhead) composition.

As previously reported [5, 20], we found that a longer (PEG6) linker promoted a faster rate of cleavage than the two shorter PEG linkers used, as well as when compared to the activity with a short and rigid piperazine linker (Figure 2c). This linker‐warhead combination was therefore chosen for use in novel PINADs targeting MALAT1. Finally, the use of an unmodified 11‐mer as a control demonstrated that the degrader selectively targets only the conjugated sequence, highlighting the requirement for induced proximity of the warhead to the RNA to achieve efficient cleavage (Figures 2d,e).

### 2.2 Weaponization of a MALAT1 Binder

**FIGURE 3:**
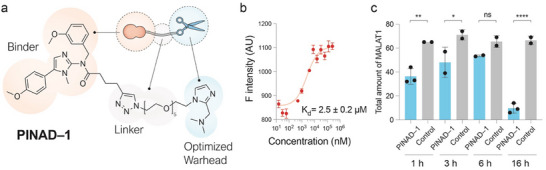
(a) Structure of PINAD–1. (b) Affinity measurements for PINAD–1 as determined via fluorescence titration using a Cy5‐tagged MALAT1 model oligomer. (c) Degradation of MALAT1 in vitro was determined via the LC–MS method at different timepoints using PINAD–1. Reaction conditions: [MALAT1] transcript = 200 µM, [PINAD–1] = 2 mM, T = 37°C. Buffer composition: 25 mM sodium cacodylate, 50 mM KCl, 1 mM MgCl_2,_ and 1 mM ZnCl_2_ at pH 6.9. ns = not significant, *p < 0.05, **p < 0.01, ***p < 0.001.

Following the discovery of a more potent warhead, we focused our efforts on weaponize two candidate MALAT1 triplex binders reported in the literature [17, 19], aiming to generate a small library of structurally diverse compounds featuring various scaffolds and modifications at different positions (Figure S2), which were then evaluated for their ability to bind the MALAT1 triplex in order to identify optimal candidates. A shorter version of the MALAT1 triplex tagged with Cy5 (MALAT1–Cy5, Figure S3) was used as a model oligonucleotide, following the approach described by Abulwerdi et al. [17]. Fluorescence titrations using MALAT1–Cy5 model oligomer showed PINAD‐1 (Figure 3a), derived from one of the binders presented by Abulwerdi et al. and weaponized with the optimal warhead/linker combination at the NH position (Figure S2, position 1), as the most promising hit with *K*_d_ = 2.5 ± 0.2 µM (Figure 3b). In contrast, the compound derived from the same binder but weaponized at the methoxy position (PINAD–1.2, Figure S2) yielded a significantly worse binding constant, *K*_d_ = 42.8 ± 3.8 µM (Figure S4a), remarking the relevance of this position in the binding process [17]. We also decided to weaponize Binder–2 (Figure S2), a compound originally presented in the previous study showing mild biological activity [17]. The resulting PINAD–2 showed a *K*_d_ value of 9.4 ± 0.5 µM (Figure S4b). To support these observations, we synthesized the analogue molecules but using a carboxyfluorescein (FAM) instead of the warhead to monitor its fluorescence rather than the one from the Cy5‐tagged oligomer, finding *K*_d_ values in the same order of magnitude, confirming the consistency of the previous claim (Figure S5).

Given their promising *K*_d_ values, the MALAT1 triplex degradation capabilities of PINAD–1 and PINAD–2 were evaluated using LC–MS. Compared to the control experiment, PINAD–1 induced significant degradation of the transcript at all time points, achieving 89% degradation after 16 h (Figure 3c). In contrast, PINAD–2 showed no significant difference, even after 16 h of incubation (Figure S6). Although we previously determined that PINAD–2 exhibited a *K*_d_ value similar to that of PINAD–1, we hypothesize that the nature of this binding interaction plays a critical role in enabling transcript degradation. Previous studies have demonstrated similar binding affinities of Binder–1 and Binder–2 for the MALAT1 transcript [17]. However, although Binder–1 induced destabilization of the MALAT1 triplex, no comparable effect was observed with Binder–2 [17]. We propose that triplex destabilization, triggered by Binder–1 engagement, is a key factor facilitating degradation by the warhead [17]. This effect likely weakens the intramolecular interactions within the oligonucleotide, making it more susceptible to cleavage induced by the warhead.

### 2.3 Specific MALAT1 Degradation in Cells

**FIGURE 4:**
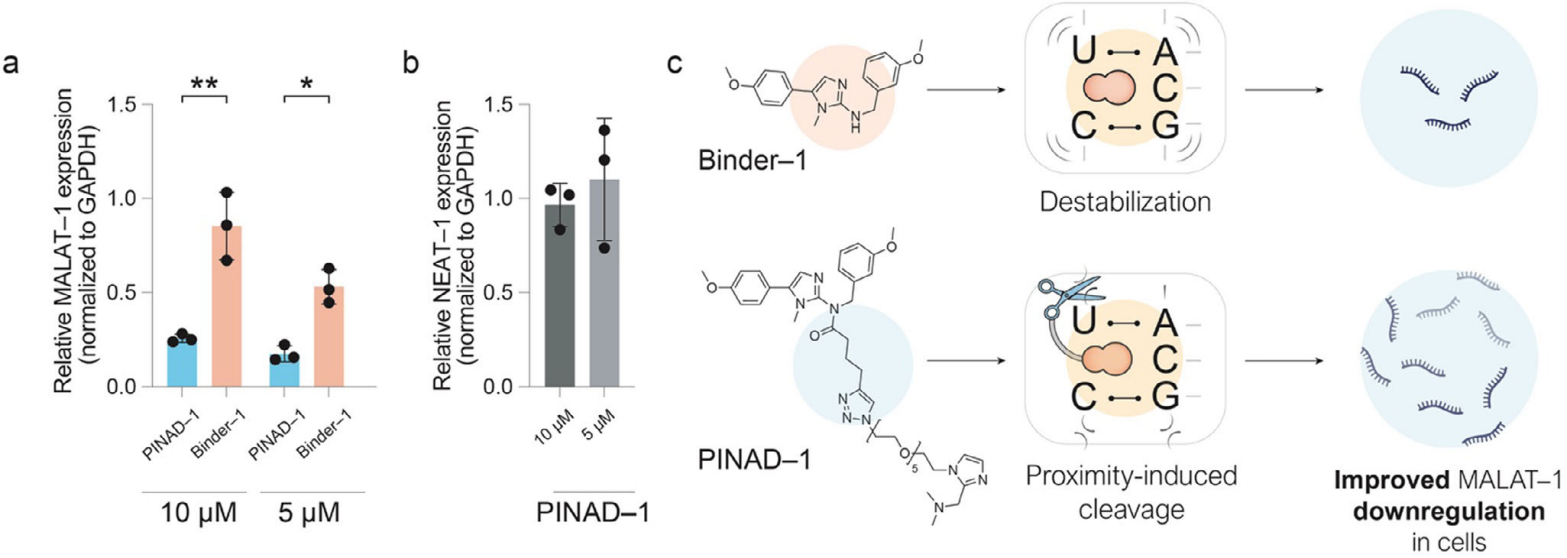
qPCR assays on MALAT1 and NEAT1 using PINAD–1. (a) Quantification of MALAT1 levels after 24 h incubation of MCF‐7 cells with PINAD–1 compared to the downregulation induced by its binder Binder–1. (b) Quantification of NEAT1 levels in MCF‐7 cells, a transcript structurally similar to MALAT1. (c) Mechanism of action of Binder–1 and PINAD–1 on MALAT1 in cells. Binder–1 binds to the triplex structure within the MALAT1 transcript, leading to its destabilization and resulting in partial downregulation of MALAT1 expression in cells. PINAD–1 targets the same structural motif; however, the attached warhead induces significant cleavage of the transcript when brought into proximity, leading to striking downregulation of the lncRNA in cells. ns = not significant, *p < 0.05, **p < 0.01, ***p < 0.001, ****p < 0.0001.

Finally, we moved on to evaluating the efficacy of our degraders in a cellular environment. We decided to focus on MCF‐7 cells due to their overexpression of, and sensitivity to, MALAT1 [22, 23]. In parallel, we also monitored the expression of NEAT1, another lncRNA transcript encoded in the same chromosomal locus. NEAT1 features a structurally similar triplex‐forming region and is considered the closest relative within the ENE (element for nuclear expression) class of MALAT1‐like lncRNAs, thus representing the standard control for MALAT1 specificity [24, 25, 26] (Figure S7). After 24 h of incubation, MALAT1 expression was analyzed using qPCR, with gene expression levels normalized to the housekeeping gene GAPDH. In agreement with the previous in vitro experiments, PINAD–1 emerged as the most effective compound, achieving an 85% reduction in MALAT1 transcript levels at 5 µM (Figure 4a). The difference in activity between both compounds reflects a substantial increase in degradation efficiency compared to Binder–1, previously reported to act via destabilization of the triplex structure alone [17], while maintaining the same specificity against the structurally related NEAT1 triplex (Figure 4b, c). Treatment with the warhead (Figure 2, compound 14A) resulted in no alteration of MALAT1 expression, highlighting the necessity of the proximity effect provided by the binder in the bifunctional molecule for effective cleavage of the target RNA (Figure S8). Consistent with our previous LC–MS results, treatment of MCF–7 cells with PINAD–2 resulted in minimal alteration of MALAT1 expression (Figure S9), further supporting our hypothesis that combining the warhead with a binder capable of partially destabilizing the target RNA is crucial for facilitating proximity‐induced cleavage. Neither PINAD–1 nor PINAD–2 was toxic after 24 h of incubation, discarding potential off–target activity due to toxicity (Figure S10).

## 3 Conclusion

Taken all together, our data further elucidates the still underexplored mechanism of RNA degradation mediated by imidazole‐based warheads, supporting the hypothesis that effective RNA degradation requires not only proximity but also a specific structural context. This is demonstrated by the stark contrast in activity between PINAD–1 and the structurally similar but functionally inert PINAD–2. In addition, these findings confirm that transcript geometry and binder‐induced destabilization play key roles in enabling selective degradation in a similar fashion to previously published studies that exploited bulge‐loop inducing expedients to facilitate specific RNA degradation [27]. While the broader cellular effects and transcriptome‐wide selectivity of PINAD–1 remain to be explored, our work establishes a framework for rational design of PINADs and provides a valuable chemical tool for the study of structured noncoding RNAs. Given the promising results with MALAT1, we foresee the application of this approach to further disease‐associated RNA targets. The ability of PINADs to induce selective degradation of structured, disease‐relevant lncRNAs may offer new therapeutic avenues, particularly in cancer and other disorders driven by RNA overexpression. Future efforts will focus on optimizing warhead reactivity, improving cellular delivery, and assessing phenotypic outcomes of transcript knockdown.

## Conflicts of Interest

G.J.L.B., S.M. and R.S.J.P. are co‐inventors on patent applications (ref. PCT/EP2021/072517, filed on 12th August 2021 and ref. PCT/EP2022/080220, filled on 28th October 2022) that describe methods for nucleic acid cleavage. All other authors declare no confilct of interests.

## Supporting information




**Supporting File 1**: Supporting Information file encompasses supporting figures and schemes depicting the synthesis of compounds used, experimental methods, transcript sequences, chemical synthesis and compound characterization data, as well as copies of ^1^H and ^13^C NMR spectra. Additional references cited within the Supporting Information (5).
