# Supplementary material for: A Bifunctional Small Molecule Degrader of the Long Noncoding RNA MALAT1 Triplex
Source: Chemistry. 2026 Feb 5;32(15):e00025. doi: 10.1002/chem.202600025 (PMC13107498; doi:10.1002/chem.202600025)
Supplement: Supplementary file 1 — Supporting File 1: Supporting Information file encompasses supporting figures and schemes depicting the synthesis of compounds used, experimental methods, transcript sequences, chemical synthesis and compound characterization data, as well as copies of 1H and 13C NMR spectra. Additional references cited within the Supporting Information (5). [file CHEM-32-e00025-s001.pdf]

SUPPORTING INFORMATION

TABLE OF CONTENTS

Supplementary **Figures S1-11** 2

Supplementary **Table S1** 11

Experimental section 11

General chemistry methods and synthetic procedures 15

^{1}H and ^{13}C NMR characterization spectra 58

References 102

Supplementary Figures S1–S10

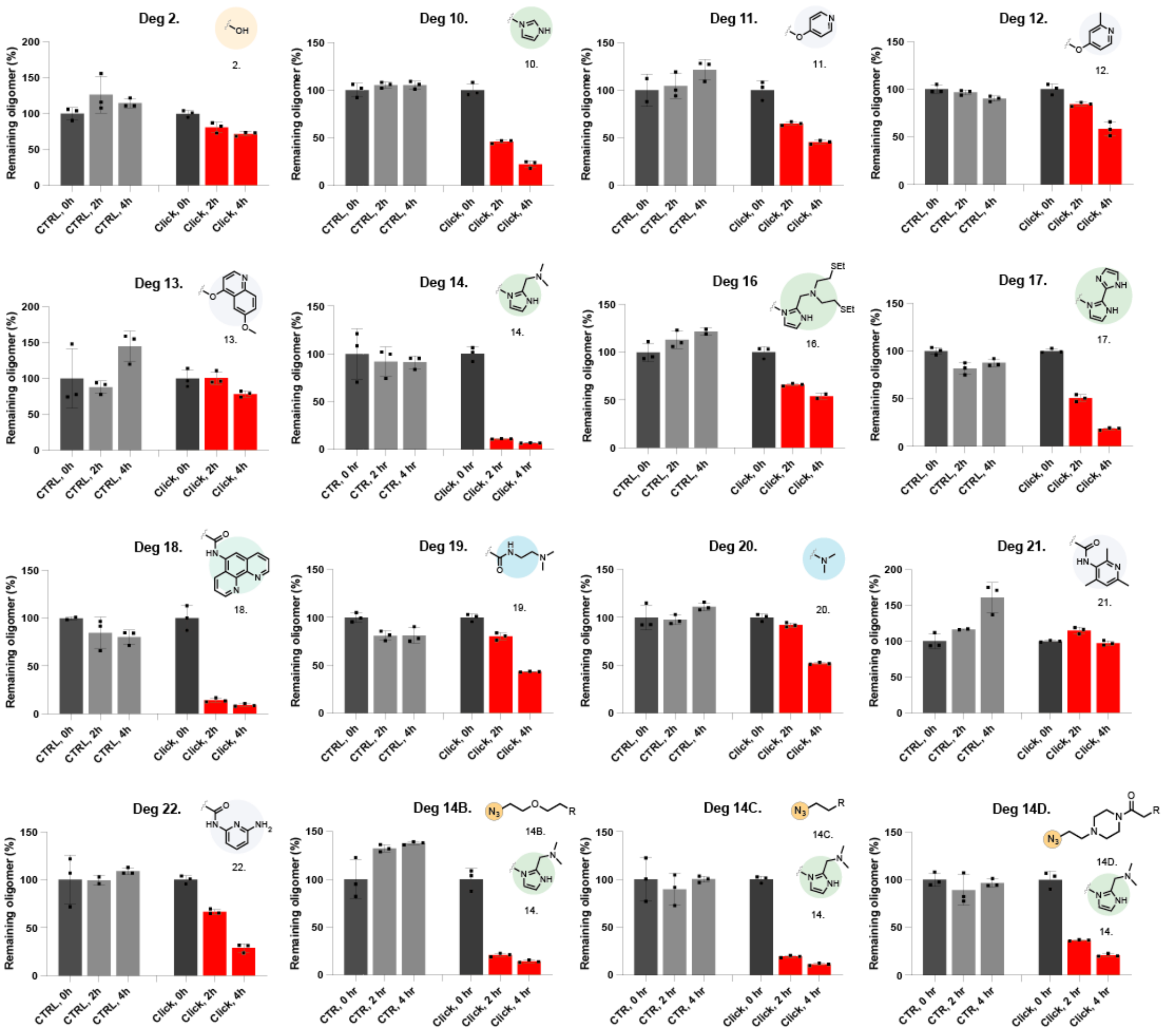

Figure S1. Cleavage of a range of degrader click conjugates incubated under CuAAC click conditions for 4 hours at 37˚C, with degrader-azide (2 mM) and alkyne (small molecule 10 mM, or RNA 200 μM), and click conditions the same as described in the method section. Quantification was performed by LC-MS as described below.

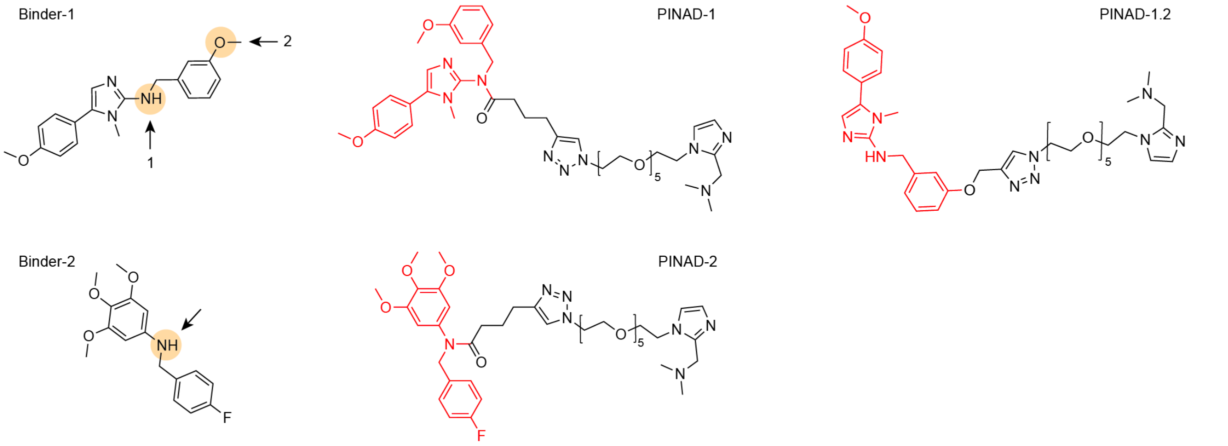

Figure S2. Chemical structures both of the weaponized MALAT–1 triplex binders and three of the PINADs readily synthetized. PINAD–1 and PINAD–1.2 share the same scaffold. Weaponization site of binders is highlighted.

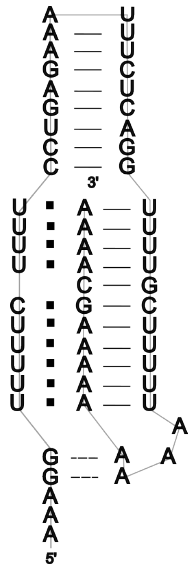

Figure S3. Schematic representation of the shortened MALAT1 triplex model oligonucleotide used in this study. Full sequence is available in Table S1.

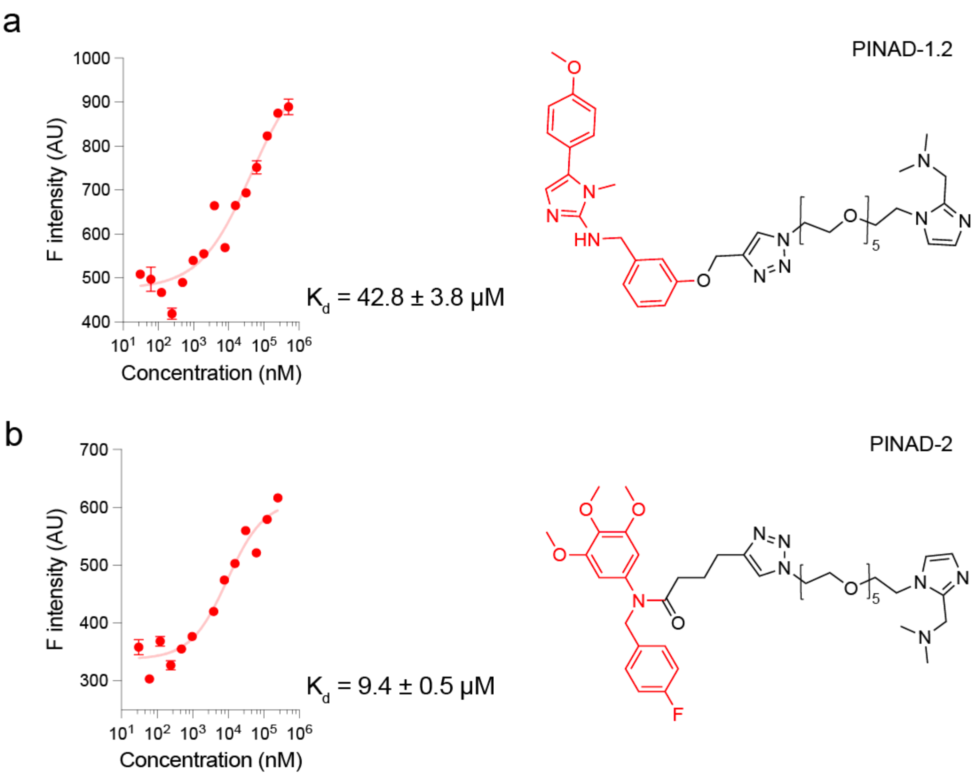

Figure S4. Fluorescence titrations of a) PINAD–1.2 and b) PINAD–2 toward Cy5–MALAT1 triplex.

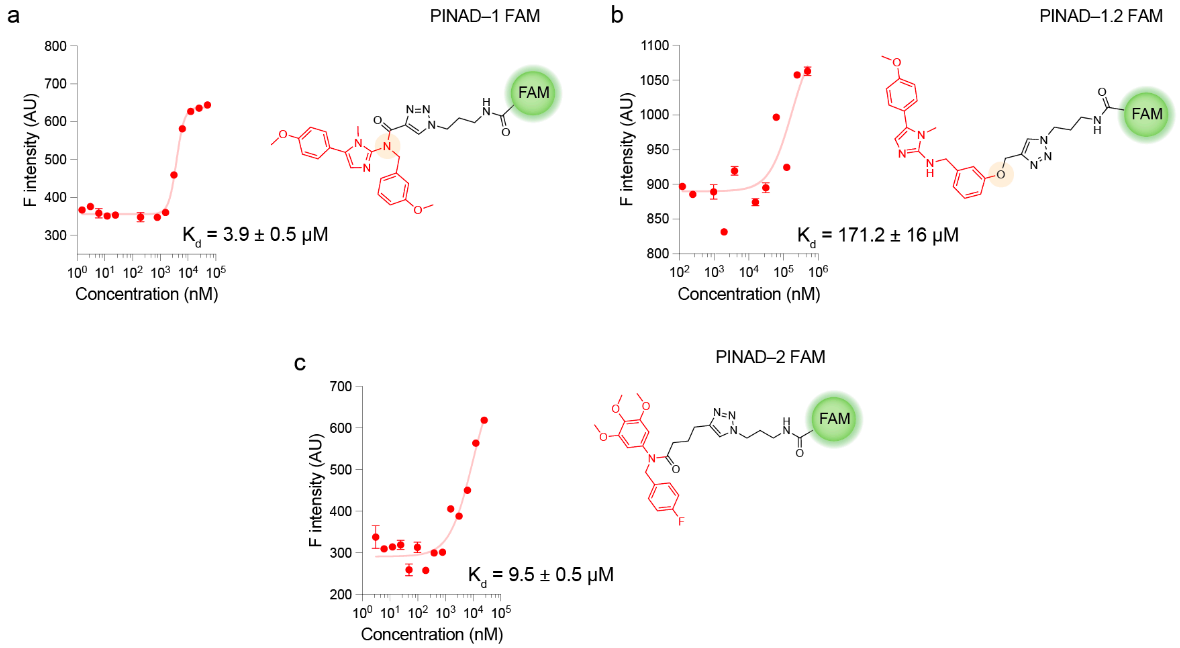

Figure S5. Fluorescence titrations of a) PINAD–1 FAM, b) PINAD–1.2 FAM and c) PINAD–2 FAM toward MALAT1 triplex.

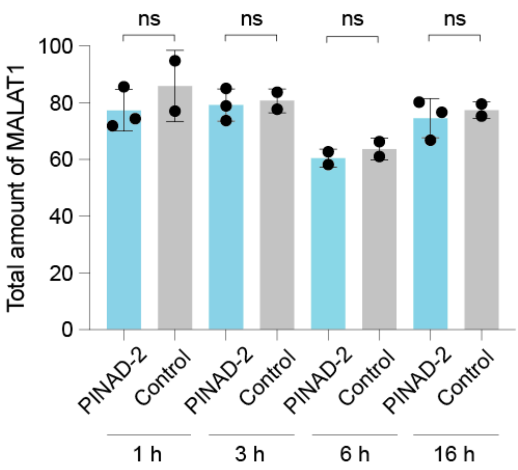

Figure S6. Degradation of MALAT1 in vitro determined via LC–MS method at different timepoints using PINAD–2. Reaction conditions: [MALAT1] transcript = 200 μM, [PINAD–2] = 2 mM, T = 37ºC. Buffer composition is described in experimental section. ns = not significant, *$p < 0.05$, **$p < 0.01$, ***$p < 0.001$.

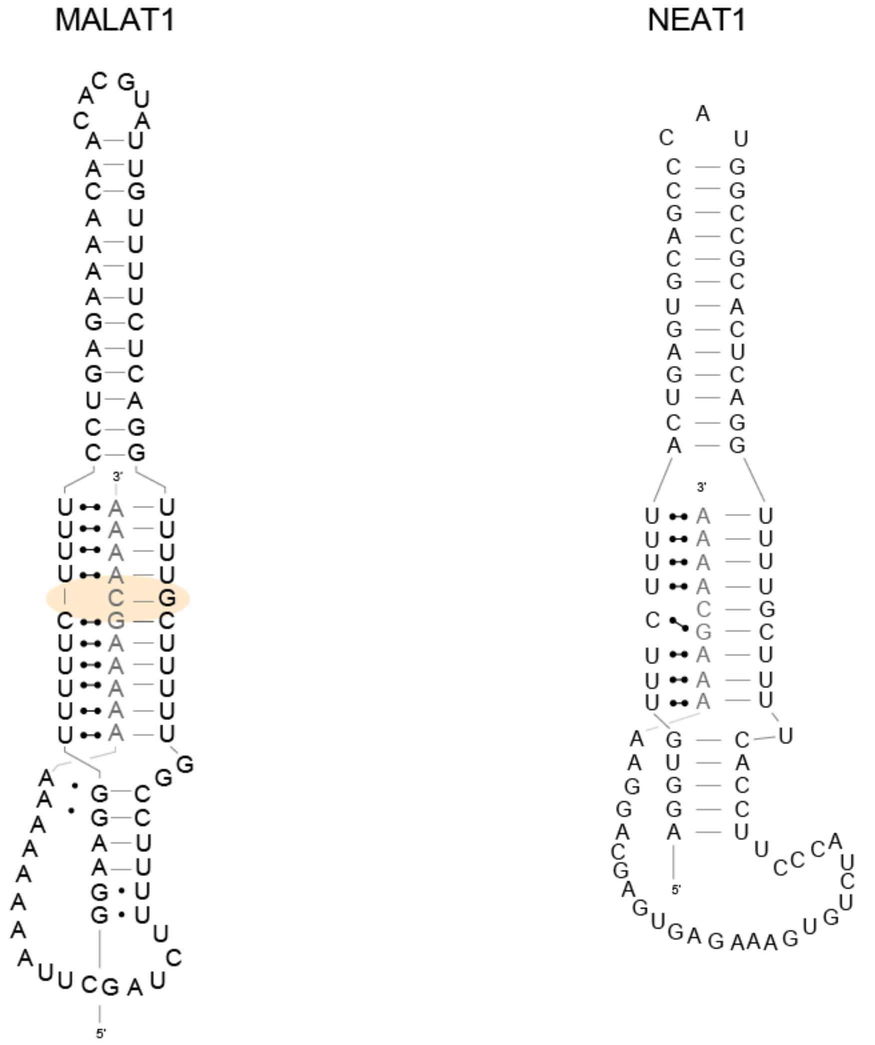

Figure S7. Secondary structures of MALAT1 and NEAT1 triple helices, respectively.

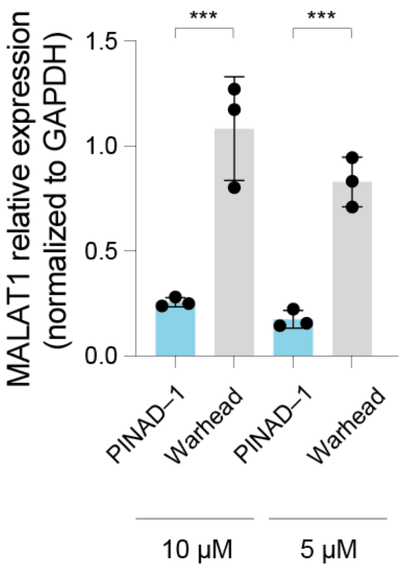

Figure S8. Quantification of MALAT1 expression in MCF–7 cells after 24h of incubation with PINAD–1 or warhead 14. [PINAD–1] or [14A] = 5 and 10 μM. ns = not significant, *p < 0.05, **p < 0.01, ***p < 0.001, ****<0.0001.

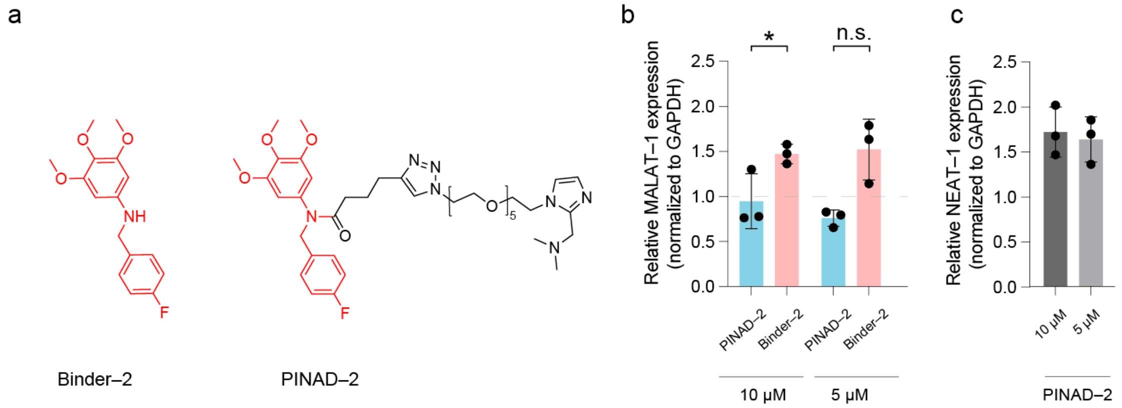

Figure S9. (a) Structures of Binder–2 and PINAD–2, compounds evaluated in the experiments shown in this figure. (b-c) Quantification of (b) MALAT1 or (c) NEAT1 levels after 24 h incubation of MCF–7 cells with PINAD–2 or Binder–2. [PINAD–2] or [Binder-2] = 5 and 10 μM. ns = not significant, *$p < 0.05$, **$p < 0.01$, ***$p < 0.001$, ****<0.0001.

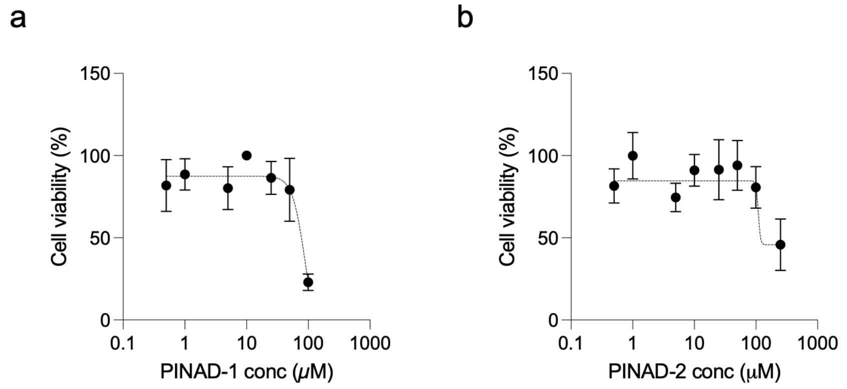

Figure S10. Cell viability assay on MCF-7 cells after 24 h incubation at 37°C with a dilution row of (a) PINAD-1 and (b) PINAD-2. The concentration-dependent curve was fitted on GraphPad Prism using the [Inhibitor] vs response – Variable slope (four parameters) equation.

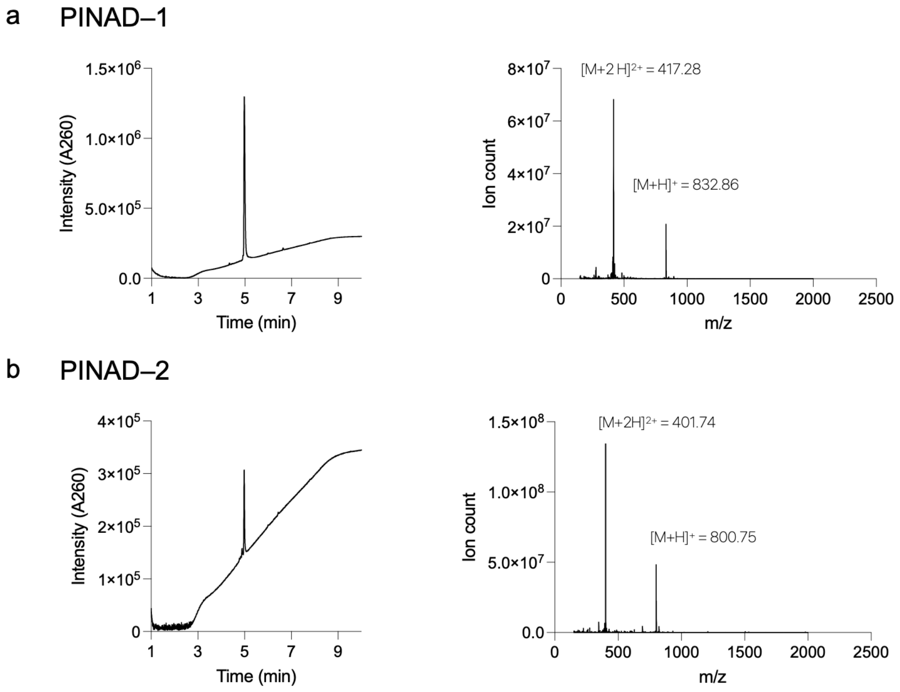

Figure S11. HPLC (A260) and MS traces of (a) PINAD–1 and (b) PINAD–2.

Supplementary Tables S1

Transcript	Sequence
Cy-5 MALAT1	Cy5_AAAGGUUUUUCUUUUCCUGAGAAAUUUCUCAGGUUUUGCUUUUUAAAAAAAAAAGCAAAA
MALAT1	AAAGGUUUUUCUUUUCCUGAGAAAUUUCUCAGGUUUUGCUUUUUAAAAAAAAAAGCAAAA
MALAT1_F primer	GCTCTGTGGTGTGGGATTGA
MALAT1_R primer	GTGGCAAAATGGCGGACTTT
NEAT1_F primer	TGGCTAGCTCAGGGCTTCAG
NEAT1_R primer	TCTCCTTGCCAAGCTTCCTTC
GAPDH_F primer	GGTGGTCTCCTCTGACTTCAACA
GAPDH_R primer	CAAAATTCGTTGTCATACCAGGAAATG

Table S1. Sequences of the transcripts used in this study. Oligomers were purchased from Integrated DNA Technologies, Inc.

Experimental section

***In vitro* degrader warhead direct functionalisation-degrader efficiency evaluation reactions.** $CuSO_4$ (final concentration 1.0 mM), THPTA (3.0 mM), degrader warhead-hexaethylene glycol-azide construct (2.0 mM) and the RNA oligo (200 µM) were added to pH 7.5 buffer (HEPES) supplemented with 10 mM MgCl2 and 100 mM KCl. CuAAC was initiated by adding NaAsc (50 mM). The reaction mixture was then incubated at 37 ºC for 10 minutes. Reaction was quenched either immediately or after a further 2- or 4-hour incubation at 37 ºC. After quenching the reaction mixtures were analysed using LC-MS. MS signal corresponding to initial degrader-functionalised RNA concentration was estimated from reactions quenched immediately after 10-minute functionalisation, by integrating 3 or 4 m/z intensities corresponding to appropriate RNA species. MS signal corresponding to 2- or 4-hour degradation was compared to the t=0 signal.

LC-MS analysis of click degrader conjugates. Conjugate cleavage was quantified by LC-MS as described previously.[1] Oligomers were analyzed using a Xevo G2-S TOF mass spectrometer coupled to an Acquity UPLC equipped with an Acquity UPLC BEH C18 1.7 µm column. A gradient of eluents composed of 16.3 mM TEA, 400 mM HFIP in H_2O and 16.3 mM TEA, 400 mM HFIP in 80:20 v/v MeCN and H_2O, with a flow rate of 0.200 mL/min, was used to separate conjugates from the reaction mixture. The change in concentration of each conjugate over time was calculated by integrating 3-4 negative mz peaks corresponding to the ion series for each conjugate and comparing these with the mean value for the same m/z peak at t_0. Percentage remaining was calculated as the mean change in peak integral assuming a linear relationship with concentration, which was shown to be an appropriate approximation for these oligomer concentrations in our previous publications.[1] Intensities of the integrated peaks were calculated in Python programming language, using Spyder interface. For control experiments quantifying changes in concentration of small molecule conjugates rather than oligomeric conjugates, the same setup was used, except that only two positive m/z peaks were present for use in quantification. The eluent in this case was replaced with a gradient of water/acetonitrile supplemented with 1% formic acid.

Oligonucleotide preparation for binding studies. To prepare samples for binding studies, the Cy5-tagged MALAT1 oligonucleotide was dissolved in the appropriate folding buffer (25 mM sodium cacodylate, 50 mM KCl and 1 mM $MgCl_2$ at pH 6.9) to a final concentration of 100 nM, then incubated at 95°C for 3 min, snap-cooled on ice for 10 minutes and slowly equilibrated to room temperature for 1 h.

Fluorescence titration assay. Titration assays were performed using the START Cap-Scan option available on a Monolith NT.115 system (NanoTemper Technologies). The experiment consisted in a dilution row for each compound of interest ranging from a final concentration of conjugate ranging from 500 μM to 0.3 nM against a constant concentration of the 5′-Cy5-labeled MALAT1 triplex construct equal to 50 nM. Each sample has been prepared and run accordingly to the NanoTemper MST handbook. Finally, initial fluorescence scans were performed in triplicate. The dissociation constant was then determined on GraphPad Prism by fitting the concentration-dependent curve ([Agonist] vs. response -- Variable slope (four parameters) or [Agonist] vs. response (three parameters) for **PINAD–1**).

In vitro MALAT1 triplex oligo degradation LC-MS assay

Oligonucleotide preparation for degradation studies. To prepare samples for degradation studies, the 59nt MALAT1 construct was dissolved in the appropriate folding buffer (25 mM sodium cacodylate, 50 mM KCl and 1mM $MgCl_2$ at pH 6.9) to a final concentration of 500 μM, then incubated at 95°C for 3 min, snap-cooled on ice for 10 minutes and slowly equilibrated to room temperature for 1 h.

LC-MS analysis of oligonucleotides. Folded MALAT1 triplex construct was added to a solution of conjugate in a degradation-enabling buffer solution (25 mM sodium cacodylate, 50 mM KCl, 1mM $MgCl_2$ and 1 mM $ZnCl_2$ at pH 6.9) to a final concentration of 200 μM RNA transcript and 2 mM selected degrader. Control samples testing the effect of zinc-mediated degradation on the triplex structure were prepared by dissolving 200 μM RNA transcript in the same buffer solution. The mixture was incubated at 37 ºC and checked for degradation after 1, 3, 6 and 16 h. The reaction mixtures were analyzed by LC-MS Xevo G2-S TOF mass spectrometer coupled to an Acquity UPLC system using an Acquity UPLC BEH C18 1.7 μm column and two mobile phases: 16.3 mM TEA, 400 mM HFIP in H_2O and 16.3 mM TEA, 400 mM HFIP in 80:20 v/v MeCN and H_2O, with a flow rate of 0.200 mL/min.

Degradation analysis. Peaks from the negative ion series were analyzed utilizing MassLynx software. Three m/z values were chosen for their peaks to be integrated with GraphPad Prism and their average values were confronted with the ones obtained at different timepoints. Deconvoluted mass spectra were reconstructed from the negative ion series using the MaxEnt algorithm on MassLynx software (v. 4.1 from Waters).

PCR analysis

Cell culture. MCF7 cells were maintained in Dulbecco's Modified Eagle Medium (DMEM) medium (1X) + GlutaMAX™ supplemented with 4.5 g/L D-Glucose and 10% fetal bovine serum (FBS) at 37 °C and 5% CO_2 in adherent tissue culture flasks.

Cell incubation with compounds. MCF7 cells were split in a 96-well plate to a concentration of 1 million cells in 70 µL PBS. Then each well was treated with 70 µL of degrader/control with variable concentration to create a dilution row. Cells were incubated for 24 h at 37°C and 5% CO_2.

RNA extraction. PBS was removed from the wells and the cells were treated with QIAzol following the user manual to completely lysate them. The total RNA thus obtained was then purified with Direct-zol RNA Miniprep kit following the manufacturer's protocol to obtain clear RNA samples. RNA concentration was then analyzed by NanoDrop™ 2000.

Reverse transcription. To perform a RT-PCR experiment, 700 ng of total RNA were added to 6 µL of SuperScript™ VILO™ Master Mix and DEPC-treated RNAse-free water was added to obtain a final volume of 35 µL. Samples were then subjected to 40 thermocycles (94°C for 30 s, 60°C for 30 s and 72°C for 30 s) to obtain the converted and amplified cDNA. For Figure S10, cDNA was synthesised from 1 µg of total RNA using SuperScript IV VILO (Invitrogen) following the manufacturer's instructions.

Quantification of MALAT1 via qPCR. 5 µL of each sample were introduced into 6 different wells of a 96-well white bottom plate, where they were mixed with a master solution of 25 µL of SYBR Green Master Mix and the required amount of primers to achieve a concentration of 300 nM (3 wells for MALAT1 primers, 3 wells for GAPDH primers). DEPC-treated RNAase-free water was finally added to reach a final volume of 50 µL in each well. Samples were run on a QuantStudio™ 5 real-time PCR machine using a 95°C for 15 s, 60°C for 15 s and 68°C for 1 minute thermal-cycle for 40 cycles followed by a standard melting point assay pre-programmed on the instrument. Relative quantification of target gene expression was performed using the comparative cycle threshold (CT) method.

The primer sequences are listed in the Supplementary Table 1. All data were plotted using GraphPad Prism (Version 9).

Cell viability analysis. A cell viability assay was performed using the MCF-7 cell line with the CellTiter-Glo kit, following the manufacturer's instructions. For this experiment, 2,500 cells per well were seeded in a 96-well plate and incubated with a dilution series of PINAD-1 (0, 0.5, 1, 5, 10, 25, 50, 100 μM) and PINAD-2 (0, 0.5, 1, 5, 10, 25, 50, 100, and 250 μM) for 24 h at 37 °C and 5% CO_2. The [PINAD-1] = 250 μM data point could not be collected due to solubility issues. Luminescence was measured using a SpectrMax i3x plate reader. The concentration-dependent curve was fitted on GraphPad Prism using the [Inhibitor] vs response – Variable slope (four parameters) equation.

General chemistry methods and synthetic procedures

17-hydroxy-3,6,9,12,15-pentaoxaheptadecyl 4-methylbenzenesulfonate (1)

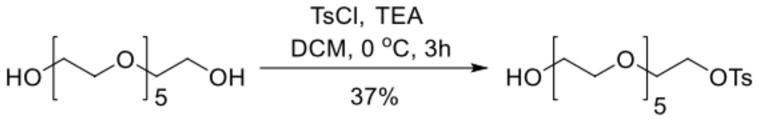

Hexaethylene glycol (3.0 g, 11 mmol) was dissolved in DCM and cooled to 0˚C, followed by addition of p-toluenesulfonyl chloride (2.2 g, 12 mmol) and TEA (2.1 g, 2.9 ml, 21 mmol). The solution was stirred for 3 h at 0˚C and 30 min at room temperature and quenched with H_2O (20 ml). Organic solvents and volatiles were removed *in vacuo*, followed by silica column purification (dry loading, gradient EtOAc with 5% MeOH to EtOAc with 10% MeOH). The product was obtained as a colourless oil (1.72 g, 3.9 mmol, 37%).

^{1}H NMR (700 MHz, CDCl3) δ 7.82 (d, J = 8.3 Hz, 2H), 7.36 (d, J = 7.9 Hz, 2H), 4.20 – 4.17 (m, 2H), 3.76 – 3.74 (m, 2H), 3.72 – 3.70 (m, 2H), 3.69 – 3.62 (m, 14H), 3.62 – 3.60 (m, 4H), 2.48 (s, 1H), 2.47 (s, 3H).

^{13}C NMR (176 MHz, CDCl3) δ 144.79, 133.04, 129.84, 128.00, 72.52, 70.70, 70.61, 70.57, 70.55, 70.52, 70.50, 70.48, 70.24, 69.30, 68.70, 61.73, 21.66.

MS [+ scan] calculated m/z $C_{19}H_{32}O_9S$ 436.18; observed 437.18 (M+H).

17-azido-3,6,9,12,15-pentaoxaheptadecan-1-ol (2)

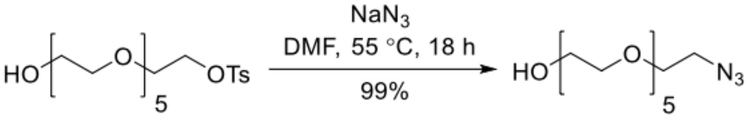

17-hydroxy-3,6,9,12,15-pentaoxaheptadecyl 4-methylbenzenesulfonate (524 mg, 1.2 mmol) was dissolved in anhydrous DMF (3 ml). Sodium azide (112 mg, 1.7 mmol) was added, the mixture was placed under N_2 and stirred for 18 h at 55˚C. Solvent was removed in vacuo. To remove traces of DMF the residue was co-evaporated successively with portions of Toluene. The resulting residue was dissolved in DCM and filtered. The resulting residue was purified by silica column (gradient, 9:1 DCM:MeOH). The product was obtained as a pale-yellow oil (200 mg, 0.64 mmol, 54%).

^{1}H NMR (500 MHz, $CDCl_3$) δ 3.73 (t, J = 4.3 Hz, 2H), 3.71 – 3.64 (m, 18H), 3.64 – 3.60 (m, 2H), 3.41 (t, J = 5.1 Hz, 2H), 2.29 (s, 1H).

^{13}C NMR (126 MHz, $CDCl_3$) 72.80, 70.63, 70.61, 70.59, 70.58, 70.51, 70.47, 70.45, 70.15, 70.02, 61.66, 50.68.

HR-MS [+ scan] calculated m/z $C_{12}H_{25}N_3O_6$ 307.17 observed 330.1641 (M+Na).

17-azido-3,6,9,12,15-pentaoxaheptadecyl 4-methylbenzenesulfonate (3)

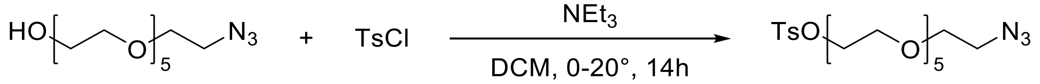

A solution of O-(2-Azidoethyl)hexaethylene glycol (0.595 g, 1.90 mmol) and TEA (0.540 mL, 3.87 mmol) in anhydrous DCM (13 mL) was purged with N_2 at 0 ℃ for 15 minutes. Tosyl chloride (0.406 g, 2.13 mmol) was added, solution degassed for a further 10 minutes, then stirred under nitrogen for 2 hours at 0 ℃ and then overnight at ambient temperature. Water (15 mL) was added, and then the product was extracted by washing the aqueous phase with DCM (3 × 15 mL). Volatiles were removed in vacuo followed by purification via flash column chromatography on silica, eluting with EtOAc. The dried product was collected as a colourless oil (0.615 g, 1.33 mmol, 70%).

^{1}H NMR (700 MHz, $CDCl_3$) δ 7.82 (d, *J* = 8.3 Hz, 2H), 7.37 (d, *J* = 7.8 Hz, 2H), 4.20 – 4.17 (m, 2H), 3.72 – 3.63 (m, 16H), 3.61 (s, 4H), 3.41 (t, *J* = 5.1 Hz, 2H), 2.47 (s, 3H).

^{13}C NMR (176 MHz, $CDCl_3$) δ 144.80, 133.04, 129.84, 128.01, 70.77, 70.71, 70.69, 70.64 (2C), 70.60, 70.58, 70.54, 70.04, 69.26, 68.70, 50.71, 21.66.

HR-MS [+ scan]: calculated m/z $C_{19}H_{31}N_3O_8S$ 461.18; observed 484.1723 (M+Na).

2-(2-azidoethoxy)ethyl 4-methylbenzenesulfonate (4)

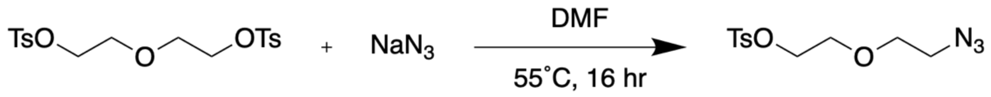

Ethylene di(p-toluenesulfonate) (2.50 g, 6.0 mmol) in anhydrous DMF (15 mL) was degassed by bubbling with nitrogen for 10 minutes. Sodium azide (0.390 g, 6.0 mmol) was added, the mixture purged for a further 20 minutes, and then stirred overnight at 55℃ under nitrogen. Solvent was removed in vacuo using portions of toluene, then the product purified via flash column chromatography on silica, eluting with Hexane:EtOAc (3:2). The product was collected and dried as a colourless oil (726 mg, 2.54 mmol, 42%).

^{1}H NMR (400 MHz, $CDCl_3$) δ 7.83 (d, J = 8.3 Hz, 2H), 7.37 (d, J = 8.1 Hz, 2H), 4.23 – 4.16 (m, 2H), 3.80 – 3.67 (m, 2H), 3.66 – 3.59 (m, 2H), 3.34 (t, J = 5.1 Hz, 2H), 2.47 (s, 3H).

^{13}C NMR (101 MHz, $CDCl_3$) δ 144.92, 132.91, 129.86, 128.00, 70.20, 69.12, 68.71, 50.64, 21.66.

HR-MS [+ scan]: calculated m/z $C_{11}H_{15}N_3O_4S$ 285.08; observed 308.0678 (M+Na).

2-Azidoethan-1-ol (5)

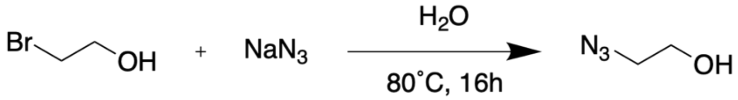

A solution of 2-bromoethan-1-ol (1.43 g, 11.4 mmol) and sodium azide (2.23 g, 34.2 mmol) in water (12 mL) was stirred at 80 ℃ overnight. This was extracted with ether (4 × 15 mL) and then the solvent cautiously removed under reduced pressure yielding a colourless oil (990 mg, 84% purity, 9.5 mmol, 83%) used without further purification.

^{1}H NMR (700 MHz, $CDCl_3$) δ 3.80 (t, J = 5.1 Hz, 2H) 3.47 (t, J = 5.1 Hz, 2H), 2.04 (s, 1H), residual ether 3.50 (q, J = 7.0 Hz), 1.23 (t, J = 7.0 Hz).

^{13}C NMR (176 MHz, $CDCl_3$) δ 61.47, 53.49, residual ether 65.82, 15.18.

HR-MS [+ scan]: calculated m/z $C_2H_5N_3O$ 87.0436; observed 87.0433.

2-Azidoethyl 4-methylbenzenesulfonate (6)

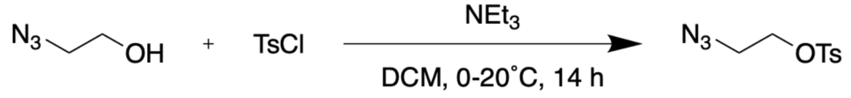

To a flask loaded with 2-azidoethan-1-ol (834 mg, 9.58 mmol) and TEA (2.67 mL, 1.94 g, 19.2 mmol) in anhydrous DCM (20 mL) on ice was added tosyl chloride (2.19 g, 11.5 mmol). The mixture was stirred overnight under N_2, warming from 0℃ to ambient temperature, then quenched with water (20 mL), and following separation the aqueous layer was extracted with DCM (20 mL). The crude was purified by flash column chromatography on silica, eluting with 25-30% EtOAc/hexane, yielding the product as a colourless oil (1.63 g, 6.76 mmol, 71%).

^{1}H NMR (500 MHz, $CDCl_3$) δ 7.85 (d, J = 8.3 Hz, 2H), 7.39 (d, J = 8.0 Hz, 2H), 4.19 (t, J = 5.1 Hz, 2H), 3.51 (t, J = 5.1 Hz, 2H), 2.49 (s, 3H).

^{13}C NMR (101 MHz, $CDCl_3$) δ 145.25, 132.61, 129.99, 127.99, 68.02, 49.59, 21.69.

HR-MS [+ scan]: calculated m/z $C_9H_{11}N_3O_3S$ 241.0419; observed 264.0424 (M+Na)

Tert-butyl 4-(2-azidoethyl)piperazine-1-carboxylate (7)

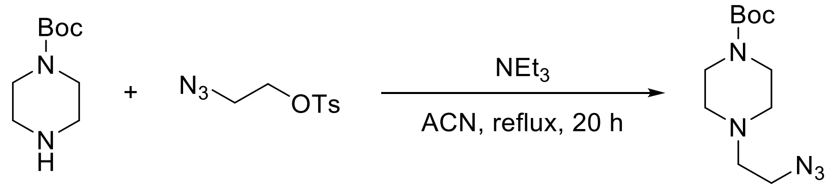

To a flask loaded with 1-Boc-piperazine (463 mg, 2.49 mmol) and TEA (0.578 mL, 419 mg, 4.14 mmol) in ACN (10 mL) was added 2-azidoethyl tosylate (500 mg, 2.07 mmol), following which the reaction mixture was refluxed for 20 hours, during which time the colour was observed to change from colourless to orange. The crude was purified by flash column chromatography on silica, eluting with EtOAc, yielding a colourless oil (401 mg, 2.07 mmol, 76%).

^{1}H NMR (500 MHz, $CDCl_3$) δ 3.47 (t, J = 5.0 Hz, 4H), 3.37 (t, J = 6.0 Hz, 2H), 2.63 (t, J = 6.0 Hz, 2H), 2.47 (t, J = 5.1 Hz, 4H), 1.48 (s, 9H).

^{13}C NMR (126 MHz, $CDCl_3$) δ 154.73, 79.70, 57.24, 52.97, 48.14, 43.07, 28.43.

HR-MS [+ scan]: calculated m/z $C_{11}H_{21}N_5O_2$ 255.18; observed 256.1767 (M+H).

1-(2-Azidoethyl)piperazine bis(2,2,2-trifluoroacetate) (8)

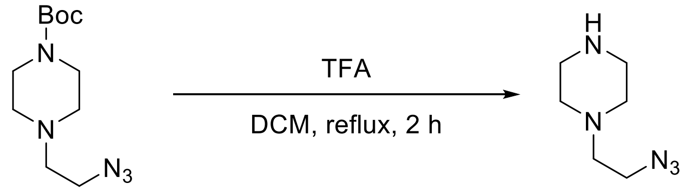

Boc deprotection was performed by refluxing the compound in 50% TFA/DCM (10 mL) for 2 hours. Solvent and excess TFA were removed in vacuo aided by two portions of toluene, yielding the product as a crystalline white salt (544 mg, 1.42 mmol, 93%).

^{1}H NMR (500 MHz, D_2O) δ 3.83 – 3.78 (m, 2H), 3.61 - 3.51 (m, 8H), 3.41 – 3.35 (m, 2H).

^{13}C NMR (126 MHz, D_2O) δ 162.98 (q, J = 35.5 Hz, TFA), 116.32 (q, J = 291.7 Hz, TFA), 55.52, 48.56, 44.74, 40.55.

HR-MS [+ scan]: calculated m/z $C_6H_{13}N_5$ 155.11171; observed 155.1173.

1-(4-(2-azidoethyl)piperazin-1-yl)-2-chloroethan-1-one) (9)

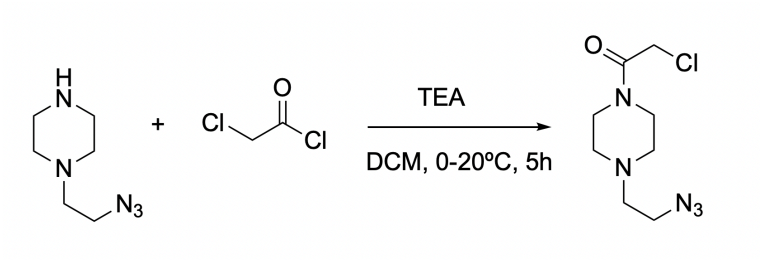

To a stirred mixture of 1-(2-azidoethyl)piperazine TFA salt (490 mg, 1.28 mmol) in DCM (7 mL) was added TEA (356 μL, 259 mg, 2.56 mmol) followed by chloroacetyl chloride (132 μL, 188 mg, 1.66 mmol). The reaction mixture was stirred for 30 minutes on ice and for three hours at room temperature, then quenched with water (7 mL). The aqueous layer was extracted with DCM (2 x 7 mL), then TEA (0.5 mL) was added before again extracting with DCM (2 x 7 mL). The product was purified by silica column chromatography eluting with 0-5% MeOH/EtOAc, yielding the product as a colourless oil (207 mg, 1.28 mmol, 70%).

^{1}H NMR (500 MHz, $CDCl_3$) δ 4.09 (s, 2H), 3.70 (t, J = 5.2 Hz, 2H), 3.62 – 3.56 (m, 2H), 3.41 (m, 2H), 2.71 – 2.66 (m, 2H), 2.63 (t, J = 5.1 Hz, 2H), 2.57 (t, J = 5.2 Hz, 2H).

^{13}C NMR (126 MHz, D_2O) δ 165.08, 56.92, 53.01, 52.44, 47.97, 46.09, 41.93, 40.78.

HR-MS [+ scan]: calculated m/z $C_8H_{14}ClN_5O$ 231.10; observed 232.0962 (M+H).

1-(17-azido-3,6,9,12,15-pentaoxaheptadecyl)-1H-imidazole (10)

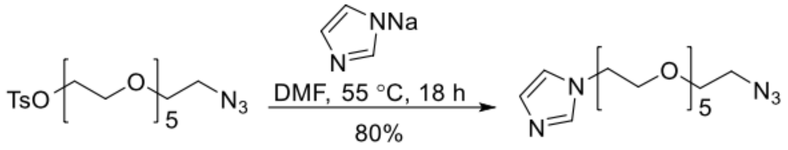

Imidazole (44 mg, 0.65 mmol) and NaH (60% dispersion in mineral oil, 26 mg, 0.65 mmol) were suspended in anhydrous DMF (2 ml) at 0 ºC. The mixture was placed under N_2 atmosphere, allowed to warm to room temperature and stirred for 30 min. 17-azido-3,6,9,12,15-pentaoxaheptadecyl 4-methylbenzenesulfonate (250 mg, 0.54 mmol) was dissolved in anhydrous DMF (3 ml) and the resulting solution was added to the first mixture. It was then stirred for 20 h at 55 ºC. Solvent was then removed *in vacuo* and the resulting residue was purified via flash chromatography (dry loading, gradient EtOAC to 9:1 EtOAc: MeOH). The product was obtained as a colourless oil (154 mg, 0.43 mmol, 80%).

^{1}H NMR (700 MHz, $CDCl_3$) δ 7.92 (s, 1H), 7.16 (s, 1H), 7.12 (s, 1H), 4.20 (t, J = 5.1 Hz, 2H), 3.80 (t, J = 5.1 Hz, 2H), 3.71 – 3.66 (m, 12H), 3.66 – 3.62 (m, 6H), 3.41 (t, J = 5.0 Hz, 2H).

^{13}C NMR (176 MHz, $CDCl_3$) δ 136.96, 126.85, 120.05, 70.68, 70.61, 70.59, 70.56, 70.13, 70.02 (Multiple PEG peaks), 50.71, 47.73.

HR-MS [+ scan]: calculated m/z $C_{15}H_{27}N_5O_5$ 357.20; observed 358.2084 (M+H).

4-((17-azido-3,6,9,12,15-pentaoxaheptadecyl)oxy)-pyridine (11)

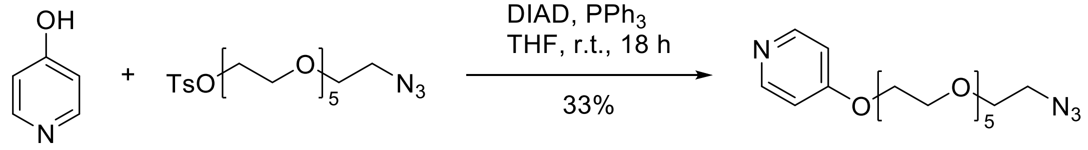

4-hydroxy-pyridine (77.4 mg, 810 µmol), hexaethylene glycol azide (200 mg, 650µmol) and triphenylphosphine (214 mg, 810 µmol) were dissolved in dry THF, degassed via freeze-pump-thaw (3 cycles) and put under 0˚C and argon atmosphere. DIAD (165 mg, 810 µmol) was then added, mixture was allowed to warm to room temperature and stirred overnight. 3 g/l solution of NH_4Cl (20 ml) was then added to quench the reaction and the resulting mixture was washed with DCM (3 x 10 ml). 1% NaOH solution (10 ml) was added to the aqueous fraction and the product was extracted with DCM (9 x 20 ml). Organic solvent was removed, the resulting yellow oil was purified on a silica column (gradient, AcOEt to AcOEt with 10% MeOH and 1% TEA. The title compound was obtained as a yellow oil (82 mg, 0.21 mmol, 33%).

^{1}H NMR (700 MHz, $CDCl_3$) δ 7.34 (d, J = 7.5 Hz, 2H), 6.33 (d, J = 7.6 Hz, 2H), 3.90 (t, J = 4.9 Hz, 2H), 3.75 (t, J = 4.9 Hz, 2H), 3.68 – 3.59 (m, 12H), 3.59 – 3.57 (m, 6H), 3.36 (t, J = 5.1 Hz, 2H).

^{13}C NMR (176 MHz, $CDCl_3$) δ 178.97, 140.31, 118.48, 70.84, 70.69, 70.68, 70.64, 70.63, 70.59, 70.57, 70.56, 70.07, 69.99, 56.62, 50.71

HR-MS [+ scan]: calculated m/z $C_{17}H_{28}N_4O_6$ 384.20; observed 385.2079 (M+H).

4-((17-azido-3,6,9,12,15-pentaoxaheptadecyl)oxy)-2-methylpyridine (12)

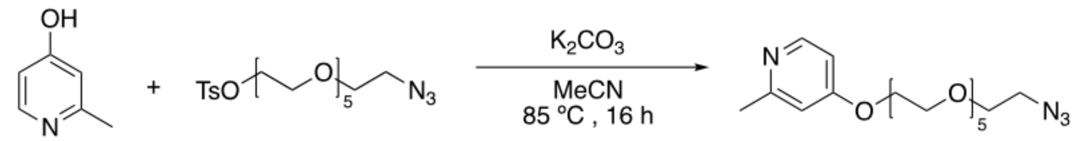

A mixture of 2-methylpyridin-4-ol (10.9 mg, 0.10 mmol, 1.0 equiv.), 17-azido-3,6,9,12,15-pentaoxaheptadecyl 4-methylbenzenesulfonate (47.3 g, 0.10 mmol, 1.0 equiv.) and K_2CO_3 (26.6 mg, 0.20 mmol, 2.0 equiv.) in acetonitrile (2 mL) was stirred under nitrogen in a sealed tube at 85 °C for 16 h. The reaction was cooled, ethyl acetate (20 mL) was added and the mixture was filtered through Celite and concentrated *in vacuo*. The resultant residue was purified on silica (eluting with 80% acetone/hexanes) to yield the title compound as a colourless oil (28.1 mg, 0.071 mmol, 71%).

^{1}H NMR (400 MHz, $CDCl_3$) δ 8.29 (d, J = 5.8 Hz, 1H), 6.68 (d, J = 2.4 Hz, 1H), 6.64 (dd, J = 5.8, 2.4 Hz, 1H), 4.15-4.13 (m, 2H), 3.86-3.84 (m, 2H), 3.71-3.69 (m, 2H), 3.67-3.64 (m, 16H), 3.37 (t, J = 5.1 Hz, 2H), 2.49 (s, 3H)

^{13}C NMR (126 MHz, $CDCl_3$) δ 165.3, 160.1, 150.4, 109.6, 107.8, 71.0, 70.8, 70.8, 70.8, 70.7, 70.7, 70.7, 70.2, 69.5, 67.2.

HR-MS [+ scan]: calculated m/z $C_{18}H_{30}N_4O_6$ 398.22; observed 399.2243 (M+H).

4-((17-azido-3,6,9,12,15-pentaoxaheptadecyl)oxy)-6-methoxyquinoline (13)

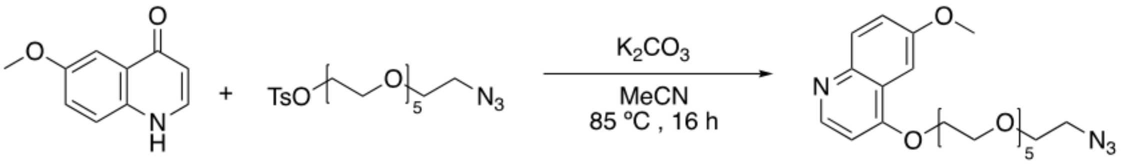

A mixture of 6-methoxyquinolin-4-ol (35.0 mg, 0.20 mmol, 1.0 equiv.), 17-azido-3,6,9,12,15-pentaoxaheptadecyl 4-methylbenzenesulfonate (92.3 g, 0.20 mmol, 1.0 equiv.) and K_2CO_3 (55.3 mg, 0.40 mmol, 2.0 equiv.) in acetonitrile (2 mL) was stirred under nitrogen in a sealed tube at 85 °C for 16 h. The reaction was cooled, ethyl acetate (20 mL) was added and the mixture was filtered through Celite and concentrated *in vacuo*. The resultant residue was purified on silica (eluting with 50% acetone/hexanes) to yield the title compound as a colourless oil (67.5 mg, 0.145
mmol, 73%).

^{1}H NMR (500 MHz, $CDCl_3$) δ 8.62 (d, *J* = 5.5 Hz, 1H), 8.08 (d, *J* = 9.2 Hz, 1H), 7.48 (d, *J* = 2.9 Hz, 1H), 7.39 (dd, *J* = 9.2, 2.9 Hz, 1H), 6.82 (d, *J* = 5.5 Hz, 1H), 4.42 (dd, *J* = 5.6, 4.2 Hz, 2H), 4.03-4.02 (m, 2H), 3.94 (s, 3H), 3.79-3.77 (m, 2H), 3.70-3.68 (m, 2H), 3.66-3.63 (m, 14H), 3.37 (t, *J* = 5.0 Hz, 2H).

^{13}C NMR (126 MHz, $CDCl_3$) δ 161.7, 157.8, 147.3, 140.8, 129.0, 123.0, 122.1, 101.1, 100.1, 71.0, 70.7, 70.7, 70.7, 70.6, 70.6, 70.0, 69.3, 68.4, 55.7, 50.7.

HR-MS [+ scan]: calculated m/z $C_{22}H_{32}N_4O_7$ 464.23; observed 465.2353 (M+H).

1-(1-(17-azido-3,6,9,12,15-pentaoxaheptadecyl)-1H-imidazol-2-yl)-*N*,*N* dimethylmethanamine (14A)

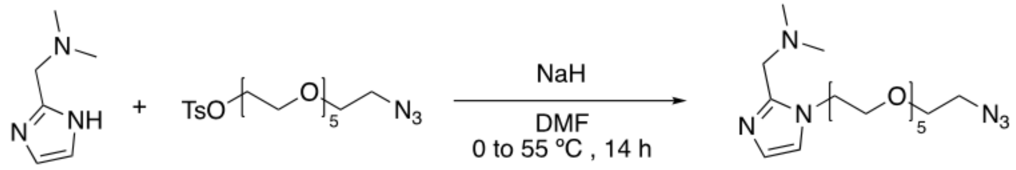

1-(1*H*-imidazol-2-yl)-*N*,*N*-dimethylmethanamine was prepared according to literature proceedings. To a solution of this compound (24.4 mg, 0.195 mmol, 1.2 equiv.) and 17-azido-3,6,9,12,15-pentaoxaheptadecyl 4-methylbenzenesulfonate (75.0 mg, 0.160 mmol, 1.0 equiv.) in DMF (2 mL) was added NaH (5.1 mg, 0.211 mmol, 1.3 equiv.) at 0 ºC, and then warmed to room temperature and stirred at 55°C for 14 h. The reaction mixture was subsequently cooled and carefully quenched with H_2O (25 mL) at 0 °C. The aqueous layer was then extracted with EtOAc (25mL × 3). The combined organic layer was dried over Mg_2SO_4 and concentrated under reduced pressure. The crude residue was purified on basic alumina, eluting with a gradient of 0% to 30% MeOH / EtOAc to yield the title compound as a colourless oil (35.1 mg, 0.085 mmol, 53%).

^{1}H NMR (500 MHz, $CDCl_3$) δ δ 7.02 (d, J = 1.3 Hz, 1), 6.94 (d, J = 1.3 Hz, 1H), 4.26 (t, J = 5.4 Hz, 2H), 3.75 (t, J = 5.4 Hz, 2H), 3.69 – 3.58 (m, 20H), 3.40 (t, J = 5.1 Hz, 2H), 2.26 (s, 6H).

^{13}C NMR (126 MHz, $CDCl_3$) δ 137.90, 127.05, 121.08, 70.74, 70.72, 70.69, 70.65, 70.64, 70.63, 70.60, 70.55, 70.05, 50.70, 45.97, 45.82, 45.14.

HR-MS [+ scan]: calculated m/z $C_{18}H_{34}N_6O_5$ 414.26; observed 415.2662 (M+H).

1-(1-(2-(2-azidoethoxy)ethyl)-1H-imidazol-2-yl)-N,N -dimethylmethanamine (14B)

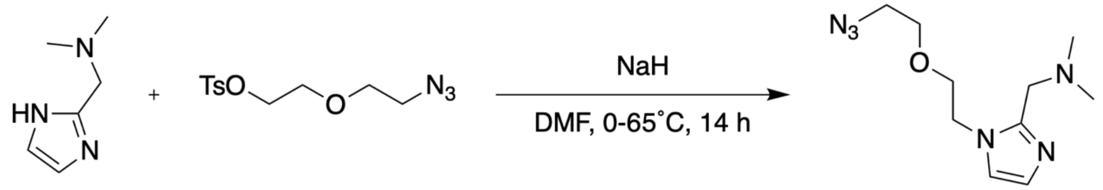

NaH (22.8 mg, 570 µmol, 60%) was added to 2-(2-azidoethoxy)ethyl 4-methylbenzenesulfonate (65.8 mg, 585 µmol) in DMF (1 mL) on ice, and degassed with N_2 for 15 mins. 2-(2-azidoethoxy)ethyl tosylate (125 mg, 438 µmol) in degassed DMF (2 mL) was added, the reaction mixture stirred for 5 minutes, removed from ice, then heated to 65℃ overnight under N_2. After 18 hours the reaction was quenched with water (50 mL), then extracted with EtOAc (3 × 50 mL). Organics were dried over $MgSO_4$, filtered and solvent removed in vacuo using toluene (25 mL) to remove residual DMF. Product was purified by flash column chromatography on silica, eluting with 15- 30% MeOH/EtOAc with 1% TEA, yielding the product as an orange wax (71.0 mg, 298 µmol, 68%).

^{1}HNMR (700 MHz, $CDCl_3$) δ 7.05 (s, 1H), 7.02 (s, 1H), 4.38 (t, J = 5.2 Hz, 2H), 3.82 (s, 2H), 3.78 (t, J = 5.2 Hz, 2H), 3.61 (t, J = 4.9 Hz, 2H), 3.38 (t, J = 4.9 Hz, 2H), 2.43 (s, 6H).

^{13}C NMR (176 MHz, $CDCl_3$) δ 127.37, 121.47, 70.77, 70.10, 54.55, 50.78, 46.55, 44.56.

HR-MS [+ scan]: calculated m/z $C_{10}H_{18}N_6O$ 238.15; observed 239.1613 (M+H).

1-(1-(2-azidoethyl)-1H-imidazol-2-yl)-N,N-dimethylmethanamine (14C)

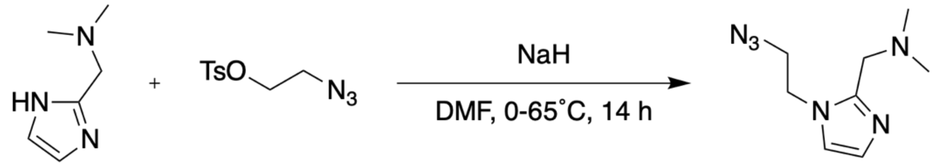

NaH (19.4 mg, 485 µmol, 60%) was added to 2-azidoethyl 4-methylbenzenesulfonate (56.0 mg, 448 µmol) in DMF (1 mL) on ice, and degassed with N_2 for 15 mins. 2-azidoethyl tosylate (90.0 mg, 373 µmol) in degassed DMF (2 mL) was added, the reaction mixture stirred for 5 minutes, removed from ice, then heated to 65℃ overnight under N_2. After 18 hours the reaction was quenched with water (50 mL), then extracted with EtOAc (3 × 50 mL). Organics were dried over $MgSO_4$, filtered and solvent removed in vacuo using toluene (25 mL) to remove residual DMF. Product was purified by flash column chromatography on silica, eluting with 0-30% MeOH/EtOAc with 1% TEA, yielding the product as an orange wax (45.0 mg, 232 µmol, 62%).

^{1}H NMR (500 MHz, $CDCl_3$) δ 7.00 (d, J = 1.3 Hz, 1H), 6.98 (d, J = 1.3 Hz, 1H), 4.23 (t, J = 5.9 Hz, 2H), 3.67 (t, J = 5.9 Hz, 2H), 3.61 (s, 2H), 2.27 (s, 6H).

^{13}C NMR (126 MHz, $CDCl_3$) δ 144.33, 127.87, 120.85, 55.59, 51.83, 45.41, 44.88.

HR-MS [+ scan]: calculated m/z $C_8H_{14}N_6$ 194.13; observed 195.1343 (M+H).

1-(4-(2-azidoethyl)piperazin-1-yl)-2-(2-((dimethylamino)methyl)-1H-imidazol-1-yl)ethan-1-one (14D)

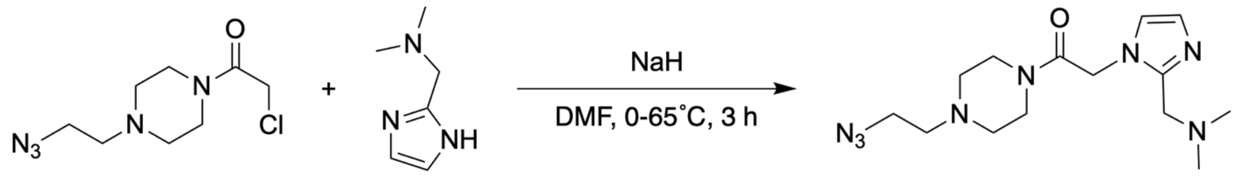

NaH (20.7 mg, 518 μmol, 60%) was added to compound 9. (59.4 mg, 475 μmol) in DMF (1 mL) on ice, and degassed with N2 for 15 mins. 1-(4-(2- azidoethyl)piperazin-1-yl)-2-chloroethan-1-one (100 mg, 432 μmol) in degassed DMF (2 mL) was added, the reaction mixture stirred for 5 minutes, removed from ice, then heated to 65°C for 3 hours. The reaction was quenched on ice with methanol (3 mL) and purified by prep HPLC, yielding the TFA salt of the product as a clear oil which crystallised on drying (173 mg, 398 μmol, 92%).

^{1}H NMR (500 MHz, $CDCl_3$) δ 7.40 (d, J = 1.7 Hz, 1H), 7.39 (d, J = 1.7 Hz, 1H), 5.40 (s, 2H), 4.46 (s, 2H), 3.95 (m, 2H), 3.93 – 3.91 (m, 4H), 3.54 (m, 2H), 3.44 – 3.42 (m, 4H), 2.90 (s, 6H).

^{13}C NMR (126 MHz, MeOD) δ 165.48, 160.36, 160.07, 139.68, 124.36, 115.78, 113.52, 55.22, 51.27, 50.59, 45.01, 42.79, 41.46, 38.87.

HR-MS [+ scan]: calculated m/z $C_{14}H_{24}N_8O$ 320.21; observed 321.2168 (M+H).

N-((1H-imidazol-2-yl)methyl)-2-(ethylthio)-N-(2-(ethylthio)ethyl)ethan-1-amine (15)

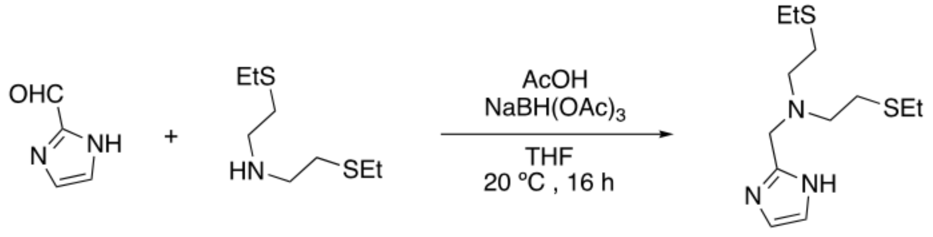

1*H*-imidazole-2-carbaldehyde (353 mg, 3.67 mmol, 1.1 equiv.) and bis(2-(ethylthio)ethyl)amine (646 mg, 3.34 mmol, 1.0 equiv.) were mixed in THF (12 mL) at rt under N_2. Glacial AcOH (0.191 mL, 3.34 mmol, 1 equiv.) was added, followed by sodium triacetoxyborohydride (1.06 g, 5.01 mmol, 1.5 equiv.) and the mixture was stirred at 20°C for 16 h. The reaction mixture was quenched with aqueous saturated $NaHCO_3$ solution (50 mL), and the product was extracted with EtOAc. The combined organic phases were dried over $MgSO_4$ and concentrated. The residue was purified by prep HPLC to give the title compound as a brown gum, which was isolated as a TFA salt (320 mg, 1.17 mmol, 35%).

^{1}H NMR (500 MHz, $CDCl_3$) δ 7.23 (s, 2H), 4.14 (s, 2H), 2.82 (dd, J = 8.1, 6.2 Hz, 4H), 2.62 (dd, J = 8.0, 6.2 Hz, 4H), 2.48 (q, J = 7.4 Hz, 4H), 1.18 (t, J = 7.4 Hz, 6H). (7.23 ppm peak overlaps with $CDCl_3$, visible by HSQC).

^{13}C NMR (126 MHz, $CDCl_3$) δ 145.65, 119.13, 53.98, 48.79, 29.21, 26.10, 14.71.

HR-MS [+ scan]: calculated m/z $C_{12}H_{23}N_3S_2$ 273.13; observed 274.1409 (M+H).

N-((1-(17-azido-3,6,9,12,15-pentaoxaheptadecyl)-1H-imidazol-2-yl)methyl)-2-(ethylthio)-N-(2-(ethylthio)ethyl)ethan-1-amine (16)

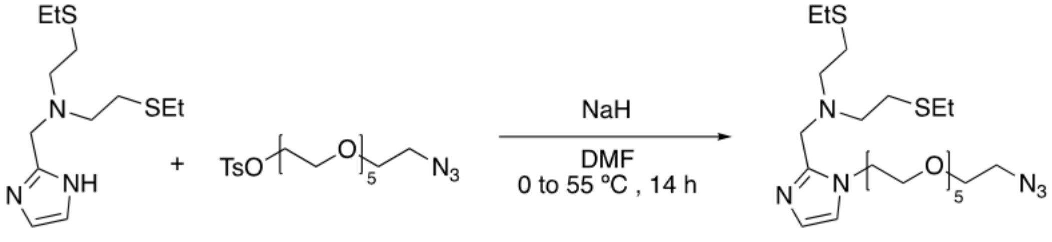

To a solution of *N*-((1H-imidazol-2-yl)methyl)-2-(ethylthio)-N-(2-(ethylthio)ethyl)ethan-1-amine (78 mg, 0.283 mmol, 1.2 equiv.) in dry DMF (2 mL) was added NaH (12 mg, 0.295 mmol, 1.25 equiv) and the resulting suspension stirred for 30 minutes at 0°C. 17-Azido-3,6,9,12,15-pentaoxaheptadecyl 4-methylbenzenesulfonate (109 mg, 0.236 mmol, 1.0 equiv.) in 1 mL dry DMF was then added at 0°C. The mixture was warmed to room temperature and stirred at 55°C for 20 h. The reaction mixture was subsequently cooled and carefully quenched with saturated $NaHCO_3$ solution (25 mL) at 0°C. The mixture was then extracted with diethyl ether (3 × 25 mL). The combined organic layer was dried over $MgSO_4$ and concentrated under reduced pressure. The crude residue was purified on silica, equilibrated with 1% NEt_3 /EtOAc. The title product was eluted with a gradient of 0% to 8% MeOH / EtOAc to yield a yellow oil (99 mg, 0.176 mmol, 74%).

^{1}H NMR (700 MHz, $CDCl_3$) δ 7.03 (s, 1H), 6.92 (s, 1H), 4.34 (t, *J* = 4.5 Hz, 2H), 3.80 (s, 2H), 3.77 (t, *J* = 5.2 Hz, 2H), 3.68-3.63 (m, 12H), 3.62-3.57 (m, 6H), 3.38 (t, *J* = 5.1 Hz, 2H), 2.72 (t, *J* = 7.2 Hz, 4H), 2.57 (t, *J* = 7.2 Hz, 4H), 2.47 (q, *J* = 7.4 Hz, 4H), 1.21 (t, *J* = 7.4 Hz, 6H).

^{13}C NMR (126 MHz, $CDCl_3$) δ 145.0,1 127.22, 121.37, 70.99, 70.96, 70.83, 70.78, 70.76, 70.75, 70.68, 70.19, 53.93, 51.66, 50.84, 46.10, 29.25, 26.16, 15.03.

HR-MS [+ scan]: calculated m/z $C_{24}H_{46}N_6O_5S_2$ 562.30; observed 563.3045 (M+H).

1-(17-azido-3,6,9,12,15-pentaoxaheptadecyl)-1H,1'H-2,2'-biimidazole (17)

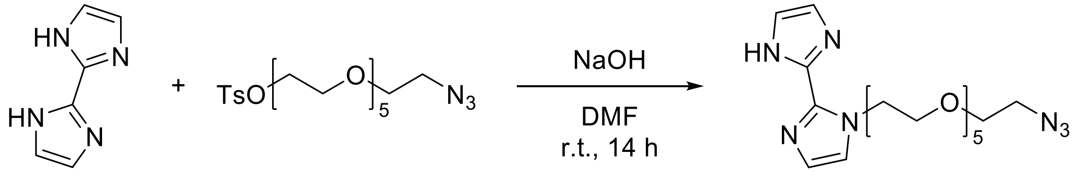

To 2,2'-biimidazole (40.0 mg, 298 µM) in anhydrous DMF (3 mL) was added NaOH (50 mg, 1.3 mmol), and the mixture stirred to a dark green solution. 17-azido- 3,6,9,12,15-pentaoxaheptadecyl 4-methylbenzenesulfonate (106 mg, 229 µM) in DMF (1 mL) was added, and the resulting purple solution stirred overnight under inert atmosphere. The reaction mixture was filtered, the residue washed with ethanol, and solvent removed in vacuo aided by portions of toluene. The product was extracted from water (40 mL) using EtOAc (3 x 40 mL), washed with brine (25 mL), dried over $MgSO_4$ and filtered, yielding an impure mixture (59 mg). Purification was performed via prep HPLC, yielding the product as a white solid (26 mg, 61 µmol, 27%).

^{1}H NMR (500 MHz, $CDCl_3$) δ 12.29 (s, 1H), 7.53 (s, 1H), 7.39 (s, 1H), 7.37 (s, 2H), 5.01 (t, J = 4.7 Hz, 2H), 3.95 (t, J = 4.6 Hz, 2H), 3.72 – 3.57 (m, 18H), 3.40 (t, J = 5.1 Hz, 2H).

^{13}C NMR (126 MHz, $CDCl_3$) δ 131.68, 126.05, 125.57, 124.45, 119.27, 70.53, 70.50, 70.43, 70.43, 70.42, 70.42, 70.39, 70.25, 69.90, 69.19, 50.65, 49.43.

HR-MS [+ scan]: calculated m/z $C_{18}H_{29}N_7O_5$ 423.22; observed 424.2303 (M+H).

20-azido-N-(1,10-phenanthrolin-5-yl)-3,6,9,12,15,18-hexaoxaicosanamide (18)

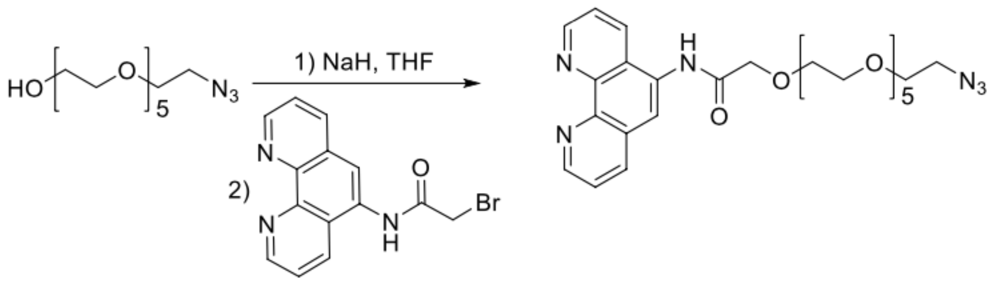

Hexaethylene glycol azide (48.7 mg, 320 µmol) was dissolved in THF (1.5 ml) and put under argon atmosphere at 0°C. NaH (60% in mineral oils, 6.3 mg, 320 µmol) was then added, mixture was then stirred for 30 minutes at 0˚C, followed by 30 minutes at room temperature. 2-bromo-N-(1,10-phenanthrolin-5-yl)acetamide (49.7 mg, 160 µmol) was then added, and the mixture was stirred at room temperature overnight. Solvent was then removed in vacuo; the resulting red oil was subjected to HPLC purification and lyophilisation. The title compound was obtained as a red oil (18.3 mg, 33.7 µmol, 17%).

^{1}H NMR (700 MHz, D_2O) δ 8.92 (dd, J = 4.4, 1.8 Hz, 1H), 8.87 (dd, J = 4.5, 1.9 Hz, 1H), 8.19 (d, J = 8.2 Hz, 1H), 8.15 (d, J = 7.9 Hz, 1H), 7.70 (s, 1H), 7.65 (dd, J = 8.4, 4.3 Hz, 1H), 7.60 (dd, J = 8.1, 4.3 Hz, 1H), 4.36 (s, 2H), 3.92 (dd, J = 4.4, 2.3 Hz, 2H), 3.84 – 3.80 (m, 2H), 3.73 – 3.66 (m, 4H), 3.60 – 3.57 (m, 2H), 3.52 (t, J = 5.0 Hz, 2H), 3.46 – 3.40 (m, 4H), 3.37 – 3.32 (m, 8H).

^{13}C NMR (176 MHz, D_2O) δ 172.40, 150.10, 150.01, 144.62, 143.19, 136.98, 131.85, 129.44, 127.77, 124.73, 124.09, 123.67, 122.56, 70.84, 69.91, 69.85, 69.72, 69.64, 69.52, 69.46, 69.45, 69.41, 69.38, 69.35, 69.14, 50.12.

HR-MS [+ scan]: calculated m/z $C_{26}H_{34}N_6O_7$ 542.25; observed 543.2560 (M+H).

17-azido-N-(2-(dimethylamino)ethyl)-3,6,9,12,15-pentaoxaheptadecanamide (19)

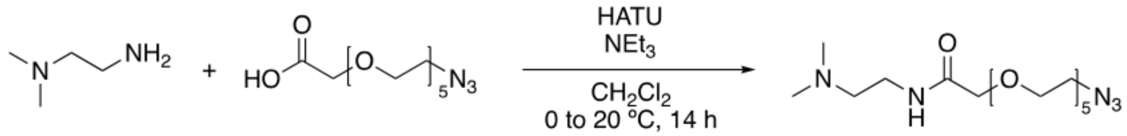

To a solution of 17-azido-3,6,9,12,15-pentaoxaheptadecanoic acid (63 mg, 0.20 mmol, 1.25 equiv.), HATU (119 mg, 0.31 mmol, 2 equiv.) and triethylamine (55 mL, 0.39 mmol, 2.5 equiv.) in dry dichloromethane stirring under a nitrogen atmosphere at 0°C was added *N'*,*N'*-dimethylethane-1,2-diamine (17 mL, 0.16 mmol, 1.0 equiv.), dropwise. The resulting mixture was stirred at 0°C for 30 mins, then warmed to 20°C and stirred for a further 14 h. The solvent was removed *in vacuo* and the residue was redissolved in EtOAc (50 mL) and transferred to a separating funnel. Saturated aqueous $NaCO_3$ (10 mL) and H_2O (40 mL) were added, the funnel shaken, and the layers separated. The aqueous layer was further extracted with EtOAc (2 × 50 mL), and the combined organic phases were dried over anhydrous $MgSO_4$ and concentrated. The residue was purified on basic alumina (eluting with a gradient of 0% to 10% MeOH/CH_2Cl_2) to yield the title compound as a colourless oil (25.0 mg, 0.064 mmol, 40%).

^{1}H NMR (500 MHz, $CDCl_3$) δ 7.15 (br. s, 1H), 3.98 (s, 2H), 3.67-3.64 (m, 18H), 3.37 (q, J = 6.0 Hz, 4H), 2.44 (t, J = 6.4 Hz, 2H), 2.25 (s, 6H).

^{13}C NMR (126 MHz, $CDCl_3$) δ 170.02, 71.06, 70.80, 70.78, 70.72, 70.71, 70.69, 70.68, 70.41, 70.14, 58.18, 50.78, 45.37, 36.50.

HR-MS [+ scan]: calculated m/z $C_{16}H_{33}N_5O_6$ 391.25; observed 392.2506 (M+H).

17-azido-*N*,*N*-dimethyl-3,6,9,12,15-pentaoxaheptadecan-1-amine (20)

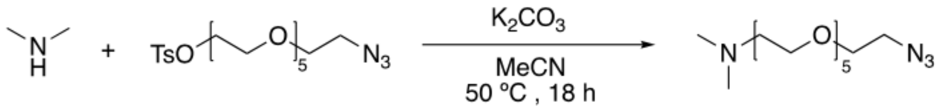

A 4 mL reaction vial was charged with 17-azido-3,6,9,12,15-pentaoxaheptadecyl 4-methylbenzenesulfonate (46.4 mg, 0.10 mmol, 1.0 equiv.) and K_2CO_3 (26.6 mg, 0.20 mmol, 2.0 equiv.), sealed and set under nitrogen. Dry acetonitrile (2 mL) was then added, followed by a 2 M solution of diethylamine in methanol (75 mL, 0.15 mmol, 1.5 equiv.). The sealed tube was then heated at 50°C for 18 h. The reaction was cooled, ethyl acetate (20 mL) was added and the mixture was filtered through Celite and concentrated *in vacuo*. The resultant residue was purified on basic alumina (eluting with ethyl acetate) to yield the title compound as a colourless oil (28.1 mg, 0.084 mmol, 84%).

^{1}H NMR (500 MHz, $CDCl_3$) δ 3.68-3.63 (m, 16H), 3.62-3.60 (m, 2H), 3.57 (t, *J* = 5.9 Hz, 2H), 3.38 (t, *J* = 5.1 Hz, 2H), 2.50 (t, J = 5.9 Hz, 2H), 2.25 (s, 6H).

^{13}C NMR (126 MHz, $CDCl_3$) δ 70.83, 70.80, 70.76, 70.72, 70.70, 70.50, 70.16, 69.46, 58.95, 50.82, 46.01.

HR-MS [+ scan]: calculated m/z $C_{14}H_{30}N_4O_5$ 334.22; observed 335.2285 (M+H).

17-azido-N-(2,4,6-trimethylpyridin-3-yl)-3,6,9,12,15-pentaoxaheptadecanamide (21)

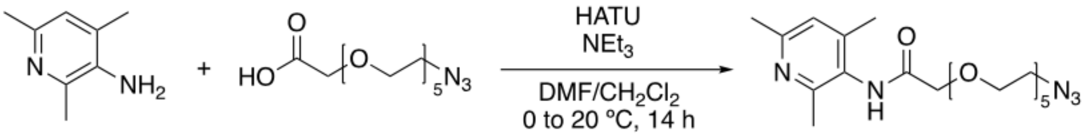

To a solution of 17-azido-3,6,9,12,15-pentaoxaheptadecanoic acid (30 mg, 0.09 mmol, 1 equiv.) in 1 mL of dry CH_2Cl_2 was added HATU (38 mg, 0.1 mmol, 1.1 equiv.), followed by triethylamine (20 mg, 0.2 mmol, 2.2 equiv) dropwise. The solution was cooled to 0°C under nitrogen atmosphere, incubated for 30 min, then a solution of 2,4,6-trimethylpyridin-3-amine (13.6 mg, 0.11 mmol, 1.1 equiv.) in 1 mL dry CH_2Cl_2 was added. The reaction was allowed to warm to 20°C and stir for 14 hours. The resulting mixture was diluted with CH_2Cl_2 and washed with saturated sodium carbonate solution (3 × 20 ml). The combined organic phases were dried over $MgSO_4$, filtered and concentrated under reduced pressure. The residue was purified by chromatography, eluting with 5% methanol in ethyl acetate to yield the title compound as a yellowish oil (5 mg, 11%, 0.011 mmol).

^{1}H NMR (500 MHz, $CDCl_3$) δ 9.10 (s, 1H), 7.16 (s, 1H), 4.22 (s, 2H), 3.85 – 3.80 (m, 2H), 3.76 – 3.71 (m, 2H), 3.70 – 3.64 (m, 2H), 3.62 (dd, J = 5.6, 4.5 Hz, 2H), 3.59 (q, J = 1.3 Hz, 4H), 3.56 – 3.54 (m, 2H), 3.53 – 3.48 (m, 2H), 3.46 – 3.40 (m, 2H), 3.36 (t, J = 5.0 Hz, 2H), 2.67 (s, 3H), 2.63 (s, 3H), 2.36 (s, 3H).

^{13}C NMR (126 MHz, $CDCl_3$) δ 169.50, 162.35, 153.12, 152.33, 130.54, 125.23, 71.58, 70.72, 70.66, 70.63, 70.58, 70.54, 70.38, 70.26, 70.07, 50.78, 20.66, 19.05, 18.07.

HR-MS [+ scan]: calculated m/z $C_{20}H_{33}N_5O_6$ 439.25; observed 440.2510 (M+H).

N-(6-aminopyridin-2-yl)-17-azido-3,6,9,12,15-pentaoxaheptadecanamide (22)

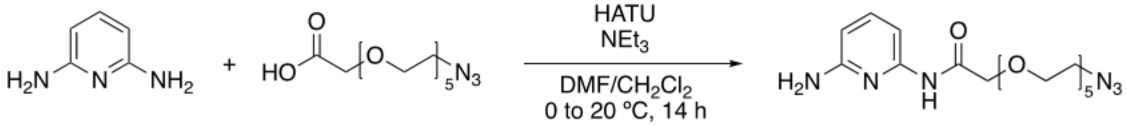

To a solution of 17-azido-3,6,9,12,15-pentaoxaheptadecanoic acid (126 mg, 0.39 mmol, 1 equiv.) in 1 mL of dry CH_2Cl_2 was added HATU (224 mg, 0.59 mmol, 1.5 equiv.), followed by triethylamine (99 mg, 0.23 mmol, 2.5 equiv) dropwise. The solution was cooled to 0°C under nitrogen atmosphere, incubated for 30 min, then a solution of 1,2-diaminopyridine (193 mg, 1.8 mmol, 4.5 equiv.) in dry CH_2Cl_2 / DMF was added. The reaction was allowed to warm to 20°C and stir for 14 hours. The resulting mixture was diluted with CH_2Cl_2 and washed with saturated sodium carbonate solution (3 × 20 ml). The combined organic phases were dried over $MgSO_4$, filtered and concentrated under reduced pressure. The residue was purified by chromatography, eluting with ethyl acetate to yield the title compound as a pale yellow oil (100 mg, 81%, 0.24 mmol).

^{1}H NMR (500 MHz, $CDCl_3$) δ 8.85 (s, 1H), 7.53 (dd, J = 7.9, 0.8 Hz, 1H), 7.44 (t, J = 7.9 Hz, 1H), 6.25 (dd, J = 7.9, 0.8 Hz, 1H), 4.44 (s, 2H), 4.10 (s, 2H), 3.78 – 3.73 (m, 4H), 3.73 – 3.69 (m, 4H), 3.69 – 3.63 (m, 10H), 3.39 (t, J = 5.1 Hz, 2H).

^{13}C NMR (126 MHz, $CDCl_3$) δ 168.60, 157.51, 149.43, 140.04, 104.62, 103.48, 71.47, 70.89, 70.83, 70.71, 70.70, 70.68, 70.41, 70.11, 50.84.

HR-MS [+ scan]: calculated m/z $C_{17}H_{28}N_6O_6$ 412.21; observed 413.2147 (M+H).

2-hydroxy-2-(4-methoxyphenyl)-methyl-2,3-dihydro-1H-imidazo[1,2-a]pyrimidin-4-ium bromide (23)

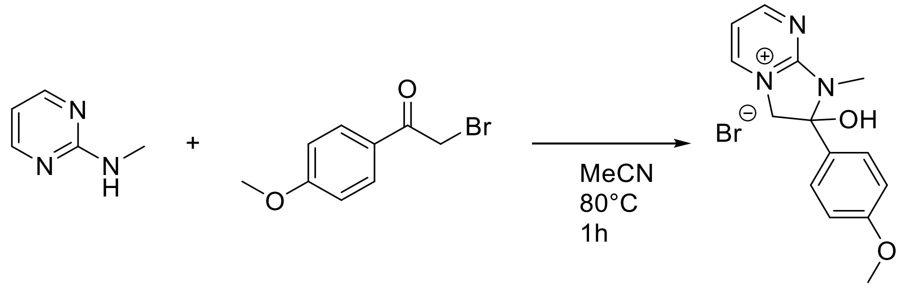

N-methylpyrimidin-2-amine (200 mg, 1.8 mmol) and 2-bromo-4'-methoxyacetophenone (567 mg, 2.5 mmol, 1.4 eqv) were dissolved in acetonitrile (10 mL). The solution was left stirring at reflux until the formation of a yellow precipitate which was then filtered to obtain the pure product (594.5 mg, 1.76 mmol, 98% yield).

^{1}H NMR (500 MHz, CD_3OD): δ 9.00 (dd, J = 4.7, 2.1, 1 H), 8.73 (dd, J = 6.4, 2.1 Hz, 1 H), 7.61 (d, J = 8.8 Hz, 2 H), 7.25 (dd, J = 6.4 Hz, 1H), 7.03 (d, J = 8.9 Hz, 2 H), 4.80 (dd, J = 43.0, 14.0 Hz, 2H), 3.86 (s, 3H), 3.02 (s, 3H).

^{13}C NMR (126 MHz, CD_3OD): δ 167.98, 160.74, 147.46, 129.15, 127.73, 113.88, 111.05, 62.88, 54.50, 25.03.

MS [+ scan]: calculated m/z $C_{14}H_{16}N_3O_2^+$ 258.12; observed 258.12

2-(4-methoxyphenyl)-1-methylimidazo[1,2-a]pyrimidin-1-ium bromide (24)

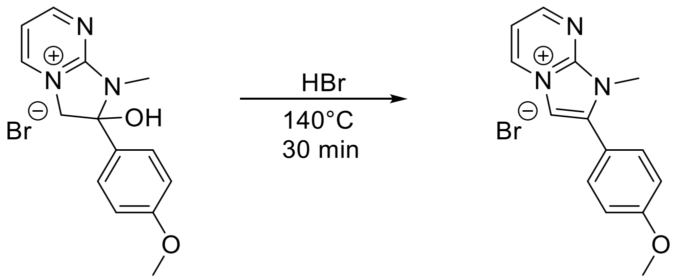

23 (100 g, 0.3 mmol) was dissolved in 33% hydrobromic acid solution in acetic acid (2 mL) and refluxed at 140°C for 30 minutes. The mixture was cooled before diluting it in tenfold toluene to precipitate a white solid that was then filtered and washed with toluene to obtain the product (95mg, 0.30 mmol, quantitative yield).

^{1}**H NMR** (400 MHz, CD_3OD): δ9.29 (dd, J = 6.7, 1.8 Hz, 1H), 9.10 (dd, J = 4.4, 1.8 Hz, 1H), 8.83 (s, 1H), 7.71 (m, 3H), 7.22 (d, J = 8.8 Hz, 2H), 4.09 (s, 3H), 3.94 (s, 3H).

^{13}**C NMR** (101 MHz, CD_3OD) δ 162.12, 156.84, 137.43, 131.00, 116.90, 114.65, 114.04, 109.90, 54.72, 30.45.

MS [+ scan]: calculated m/z $C_{14}H_{14}N_3O^+$ 240.11; observed 240.11

5-(4-methoxyphenyl)-1-methyl-1H-imidazol-2-amine (25)

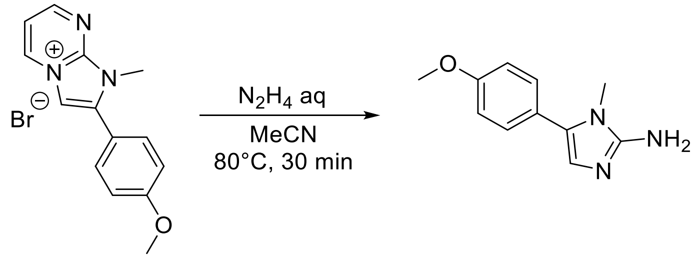

24 (100 mg, 0.31 mmol) was suspended in acetonitrile (2 mL) and hydrazine hydrate (20 µL, 0.61 mmol, 2 eqv) was added to the mixture. After the solution turned yellow, it was stirred at reflux for 2h. The mixture was concentrated under reduced pressure to facilitate precipitation of the final product that was collected by filtration to obtain a white-yellowish solid (47 mg, 0.23 mmol, 74% yield).

^{1}H NMR (500 MHz, CD_3OD): δ 7.41 (m, 2H), 7.08 (m, 2H), 6.85 (s, 1H), 3.87 (s, 3H), 3.43 (s, 3H).

^{13}C NMR (126 MHz, CD_3OD) δ 160.49, 130.19, 129.64, 124.09, 119.71, 114.11, 111.37, 54.49, 29.41.

MS [+ scan]: calculated m/z $C_{11}H_{13}N_3O$ 203.11; observed 204.11 (M+H).

3-methoxy-N-(5-(4-methoxyphenyl)-1-methyl-1H-imidazol-2-yl)benzamide (Binder-1)

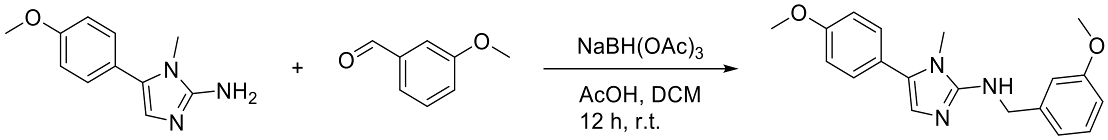

25 (100 mg, 0.49 mmol) and 3-methoxybenzaldehyde (72 mg, 0.59 mmol, 1.2 eqv) were added to a round flask with acetic acid (31 µL, 0.49 mmol, 1 eqv) and sodium triacetoxyborohydride (156 mg, 0.74 mmol, 15 eqv) in dichloromethane (2 mL). The mixture was stirred at room temperature for 12h under nitrogen atmosphere. Once the reaction reached completion, the borohydride in excess has been quenched with the addition of 1 mL of NaOH 2M in aqueous solution and 5 mL water. The mixture was the extracted with dichloromethane with the help of a couple drops of methanol. The organic phase was then dried over magnesium sulphate, filtered and concentrated under reduced pressure. The raw material was then purified by column chromatography (dichloromethane/methanol, slow gradient from 1% methanol to 10% over 20 minutes) using aluminum oxide as stationary phase to obtain the pure product as a yellowish oil (51 mg, 0.16 mmol, 52%).

^{1}H NMR (400 MHz, CD_3OD): δ 7.29 (m, 3H), 7.04 (m, 2H), 6.96 (m, 2H), 6.86 (dd, J = 8.3, 2.6 Hz, 1H), 6.73 (s, 1H), 4.56 (d, J = 5.6 Hz, 2H), 3.85 (s, 3H), 3.83 (s, 3H), 3.35 (s, 3H)

^{13}C NMR (101 MHz, $CDCl_3$) δ 159.86, 158.93, 150.35, 140.94, 129.67, 129.47, 123.21, 122.23, 120.33, 114.17, 113.72, 112.85, 55.35, 48.27, 29.87.

HR-MS [+ scan]: calculated m/z $C_{19}H_{21}N_3O_2$ 323.1645; observed 324.1718 (M+H).

Hex-5-ynoyl chloride (26)

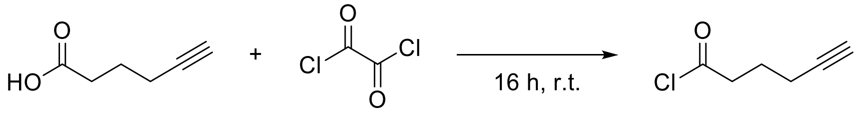

Oxalyl chloride (380 µL, 4.46 mmol, 5 eqv) was added to 5-hexynoic acid (100 mg, 0.89 mmol) while stirring under inert atmosphere. The reaction mixture was stirred overnight at room temperature before the oxalyl chloride in excess with distillation under reduced pressure to obtain the product as a brown-yellow liquid (116 mg, 0.89 mmol, quantitative yield).

^{1}H NMR (500 MHz, $CDCl_3$): δ 3.09 (t, J = 7.2 Hz, 2H), 2.33 (td, J = 6.8, 2.7 Hz, 2H), 2.04 (t, J = 2.6 Hz, 1H, -CH), 1.94 (q, J = 7.0 Hz, 2H).

^{13}C NMR (126 MHz, $CDCl_3$) δ 173.48, 82.15, 70.02, 45.60, 23.64, 17.25.

4-(1-(17-(2-((dimethylamino)methyl)-1H-imidazol-1-yl)-3,6,9,12,15-pentaoxaheptadecyl)-1H-1,2,3-triazol-4-yl)-N-(3-methoxybenzyl)-N-(5-(4-methoxyphenyl)-1-methyl-1H-imidazol-2-yl)butanamide (PINAD-1)

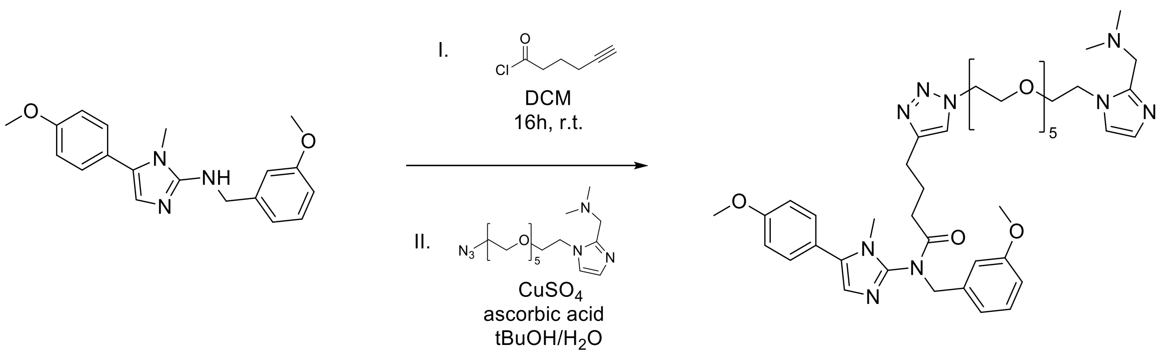

Binder-1 (100 mg, 0.31 mmol) was dissolved in dichloromethane (2 mL) with triethylamine (86 µL, 0.62 mmol, 2 eqv). The mixture was brought to 0°C before adding **26** (41 mg, 0.31 mmol, 1 eqv). The solution was left stirring at room temperature overnight and then concentrated under reduced pressure. The raw residual was then partially purified by column chromatography (dichloromethane/methanol 95:5) to obtain a yellowish liquid that wash used right away for the next synthetic step. Sodium ascorbate (24 mg, 0.12 mmol) was added to a solution of the synthetic intermediate (10 mg), warhead **14A** (10 mg, 0.024 mmol) and copper sulfate (1 mg) in water/t-butyl alcohol 1:1 (1 mL). The solution was stirred at room temperature for 30 minutes before filtering and purification by HPLC. The product is a yellow oil (3 mg, 3.6 µmol, 11.6% yield).

^{1}H NMR (700 MHz, $CDCl_3$): δ 7.60 (bs. 1H), 7.50 (d, J = 1.8 Hz, 1H), 7.41 (d, J = 1.9 Hz, 1H), 7.22-7.17 (m, 4H), 7.00 – 6.96 (m, 2H), 6.84 (dd, J = 8.3, 2.6 Hz, 1H), 6.77 (d, J = 7.6 Hz, 1H), 6.73 (bs, 1H), 5.03 (s, 2H), 4.54 (t, J = 4.8 Hz, 2H), 4.49 (bs, 2H), 3.85 (m, 6H), 3.79 (t, J = 4.7 Hz, 2H), 3.74 (s, 3H), 3.62-3.54 (m, 20H), 2.98 (bs, 2H), 2.93 (s, 6H), 2.74 (bs, 2H), 2.28-1.96 (m, 2H).

^{13}C NMR (126 MHz, $CDCl_3$) δ 171.89, 161.99, 161.68, 160.82, 159.97, 146.52, 139.72, 137.26, 136.29, 134.13, 130.50, 129.95, 124.00, 122.87, 122.56, 121.58, 119.32, 114.65, 114.44, 70.37, 70.29, 70.20, 70.13, 69.25, 69.12, 55.44, 55.29, 52.11, 50.34, 48.29, 47.40, 42.91, 33.11, 31.12, 24.34, 24.08.

HR-MS [+ scan]: calculated m/z $C_{43}H_{61}N_9O_8$ 831.4659; observed 832.4708 (M+H) and 416.7399 (M+2H).

N-(4-fluorobenzyl)-3,4,5-trimethoxyaniline (Binder-2)

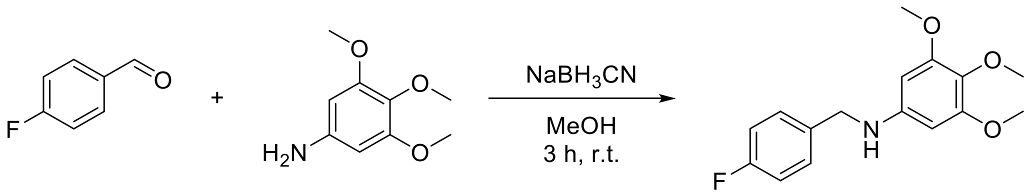

4-fluorobenzaldehyde (118 µL, 1.1 mmol), 3,4,5-trimethoxyaniline (200 mg, 1.1 mmol, 1 eqv) and sodium cyanoborohydride (346 mg, 5.5 mmol, 5 eqv) were dissolved in methanol (5 mL). The solution was left stirring at room temperature for 3h before diluting with additional methanol to quench any remaining cyanoborohydride. The mixture was dried under reduced pressure and the residues were taken up with water and extracted with dichloromethane. The reunited organic layers were then dried over magnesium sulfate, filtered, and concentrated under reduced pressure. The crude was then purified by column chromatography (petrol ether/ ethyl acetate 9:1) to obtain the product as a yellowish liquid (301 mg, 1 mmol, 94% yield).

^{1}H NMR (500 MHz, $CDCl_3$) : δ 7.26 (m, 2H), 6.94 (m, 2H), 6.57 (s, 2H), 4.28 (s, 2H), 3.84 (s, 3H), 3.78 (s, 6H).

^{13}C NMR (126 MHz, $CDCl_3$): δ 162.31, 154.02, 137.95, 132.49, 132.43, 115.84, 115.67, 99.90, 60.93, 56.18, 55.87.

MS [+ scan]: calculated m/z $C_{16}H_{18}FNO_3$ 291.13; observed 292.28 (M+H).(M+H).

N-(4-fluorobenzyl)-N-(3,4,5-trimethoxyphenyl)hex-5-ynamide (27)

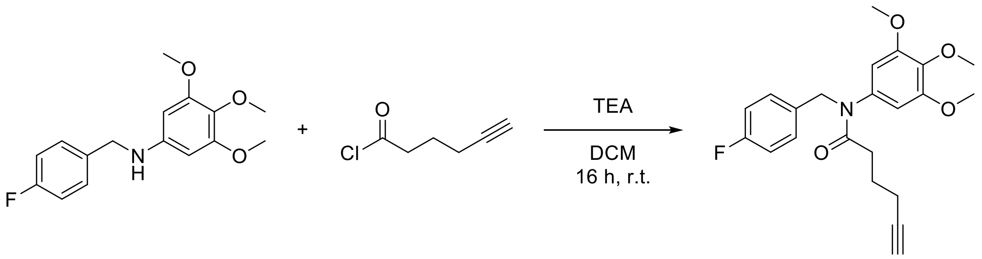

Binder-2 (100 mg, 0.34 mmol) was dissolved in dichloromethane (2 mL) and triethylamine (94 µL, 0.68 mmol, 2 eqv) was added. The solution was brought to 0°C before adding **26** (44 mg, 0.34 mmol, 1 eqv). The mixture was stirred at room temperature overnight. The crude solution was concentrated in vacuo before purification by column chromatography (dichloromethane/methanol 98:2) to obtain the product as a yellow liquid (59 mg, 0.15 mmol, 45% yield).

^{1}H NMR (500 MHz, $CDCl_3$): δ 7.22 (m, 2H), 6.98 (m, 2H), 6.13 (s, 2H), 4.81 (s, 2H), 3.87 (s, 3H), 3.73 (s, 6H), 2.27 (t, J = 7.2 Hz, 2H), 2.22 (td, J = 6.8, 2.6 Hz, 2H), 1.88-1.82 (m, 3H).

^{13}C NMR (126 MHz, $CDCl_3$): δ 172.18, 161.25, 153.58, 137.74, 137.58, 133.72, 130.93, 130.87, 115.25, 115.08, 105.73, 83.76, 68.80, 60.96, 56.15, 52.14, 32.64, 24.15, 17.86.

HR-MS [+ scan]: calculated m/z $C_{22}H_{24}FNO_4$ 385.1661; observed 386.1734 (M+H).

4-(1-(17-(2-((dimethylamino)methyl)-1H-imidazol-1-yl)-3,6,9,12,15-pentaoxaheptadecyl)-1H-1,2,3-triazol-4-yl)-N-(4-fluorobenzyl)-N-(3,4,5-trimethoxyphenyl)butanamide (PINAD-2)

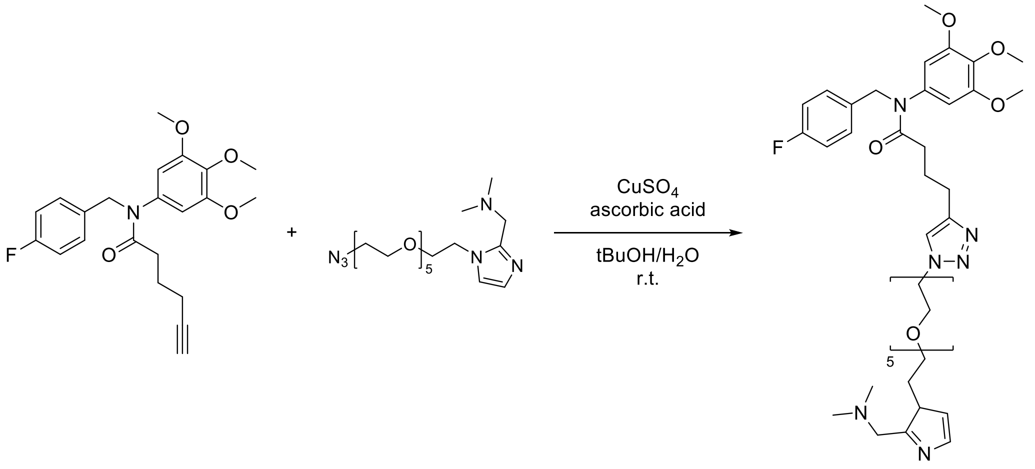

27 (30 mg, 0.075 mmol), **14A** (33 mg, 0.075 mmol, 1 eqv) and copper sulfate (3 mg, 0.015 mmol, 0.2 eqv) were dissolved in a 1:1 mixture of water and t-butyl alcohol (1 mL). Sodium ascorbate (78 mg, 0.39 mmol, 5.2 eqv) was added and the mixture was stirred at room temperature for 30 minutes. The resulting solution was then filtered and purified by HPLC to obtain a pale yellow oil (35 mg, 0.043 mmol, 58% yield).

^{1}**H NMR** (700 MHz, $CDCl_3$): δ 7.51 (m, 2H), 7.44 (bs, 1H), 7.18 (m, 2H), 6.96 (m, 2H), 6.10 (s, 2H), 5.07 (s, 2H), 4.78 (s, 2H), 4.57 (t, J = 4.7 Hz, 2H), 4.49 (t, J = 5.0 Hz, 2H), 3.88-3.84 (m, 5H), 3.79 (t, J = 4.9 Hz, 2H), 3.71 (s, 6H), 3.61-3.56 (m, 12H), 3.56-3.52 (m, 4H), 2.95 (s, 6H), 2.70 (t, J = 7.7 Hz, 2H), 2.20 (t, J = 7.2 Hz, 2H), 1.95 (p, J = 7.4 Hz, 2H).

^{13}**C NMR** (126 MHz, $CDCl_3$): δ 172.46, 163.18, 161.23, 153.61, 137.74, 137.37, 133.49, 133.46, 130.78, 130.71, 124.08, 122.51, 122.39, 115.28, 115.11, 105.55, 70.43, 70.41, 70.39, 70.37, 70.31, 70.29, 70.10, 69.79, 69.19, 69.06, 60.93, 56.15, 52.23, 50.56, 48.56, 47.30, 42.96, 33.27, 25.18, 24.51.

HR-MS [+ scan]: calculated m/z $C_{40}H_{58}FN_7O_9$ 799.4258; observed 399.7036 (M+2H) and 822.4151 (M+Na).

3',6'-dihydroxy-N-(3-(4-(4-((3-methoxybenzyl)(5-(4-methoxyphenyl)-1-methyl-1Himidazol-2-yl)amino)-4-oxobutyl)-1H-1,2,3-triazol-1-yl)propyl)-3-oxo-3Hspiro[isobenzofuran-1,9'-xanthene]-6-carboxamide (PINAD-1 FAM)

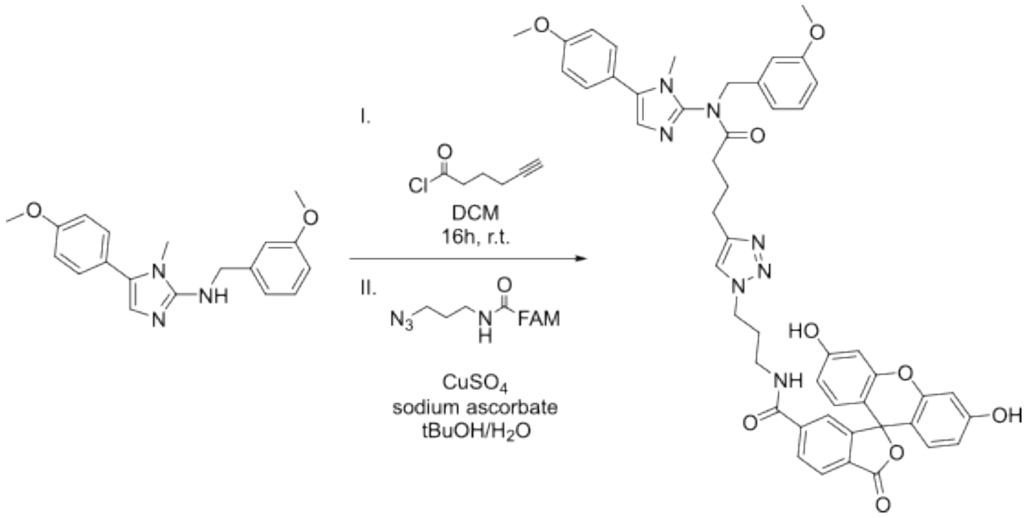

Binder-1 (100 mg, 0.31 mmol) was dissolved in dichloromethane (2 mL) with triethylamine (86 µL, 0.62 mmol, 2 eqv). The mixture was brought to 0°C before adding **26** (41 mg, 0.31 mmol, 1 eqv). The solution was left stirring at room temperature overnight and then concentrated under reduced pressure. The raw residual was then partially purified by column chromatography (dichloromethane/methanol 95:5) to obtain a yellowish liquid that was used immediately for the next synthetic step. Sodium ascorbate (24 mg, 0.12 mmol) was added to a solution of the synthetic intermediate (10 mg), N-(3-azidopropyl)-3',6'-dihydroxy-3-oxo-3H-spiro[isobenzofuran-1,9'-xanthene]-6- carboxamide (10 mg, 0.022 mmol) and copper sulphate (1 mg) in water/t-butyl alcohol 1:1 (1 mL). The solution was stirred at room temperature for 30 minutes before filtering and purification by HPLC. The product is a bright yellow oil (16.3 mg, 0.018 µmol, 81.8% yield).

^{1}H NMR (400 MHz, MeOD) δ 8.68 (s, 1H), 8.10 (d, J = 1.4 Hz, 1H), 7.73 (bs, 1H), 7.60 (s, 1H), 7.30 (d, J = 8.6 Hz, 2H), 7.23 (t, J = 7.9 Hz, 1H), 7.11 (bs, 1H), 7.01 (d, J = 8.1 Hz, 2H), 6.88 (dd, J = 8.3, 2.6 Hz, 1H), 6.81 (d, J = 7.6 Hz, 1H), 6.77 (s, 1H), 6.72 (d, J = 2.4 Hz, 2H), 6.63 (d, J = 8.7 Hz, 2H), 6.56 (dd, J = 8.7, 2.4 Hz, 2H), 5.05 (bs, 2H), 4.37 (bs, 2H), 3.84 (s, 3H), 3.74 (s, 3H), 3.34 - 3.32 (m, 5H), 3.10 (bs, 2H), 2.68 (bs, 2H), 2.12 (bs, 2H), 1.98 (bs, 2H).

MS [+ scan]: calculated m/z $C_{49}H_{45}N_7O_9$ 875.3287; observed 438.6732 (M+2H) and 876.3345 (M+H).

N-(2-(4-(4-((4-fluorobenzyl)(3,4,5-trimethoxyphenyl)amino)-4-oxobutyl)-1H-1,2,3-triazol-1-yl)ethyl)-3',6'-dihydroxy-3-oxo-3H-spiro[isobenzofuran-1,9'-xanthene]-6-carboxamide (PINAD-2 FAM)

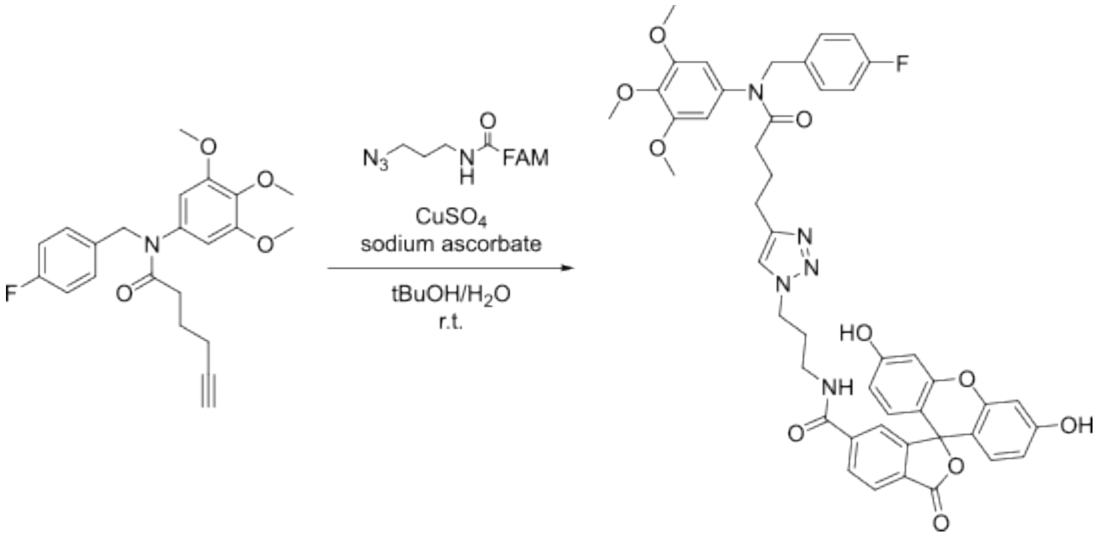

27 (30 mg, 0.075 mmol), N-(3-azidopropyl)-3',6'-dihydroxy-3-oxo-3Hspiro[isobenzofuran-1,9'-xanthene]-6-carboxamide (34 mg, 0.075 mmol, 1 eqv) and copper sulphate (3 mg, 0.015 mmol, 0.2 eqv) were dissolved in a 1:1 mixture of water and t-butyl alcohol (1 mL). Sodium ascorbate (78 mg, 0.39 mmol, 5.2 eqv) was added and the mixture was stirred at room temperature for 30 minutes. The resulting solution was then filtered and purified by HPLC to obtain a pale yellow oil (27 mg, 0.032 mmol, 43% yield).

^{1}H NMR (400 MHz, MeOD) δ 8.16 (s, 2H), 7.68 (s, 1H), 7.63 (s, 1H), 7.23 (m, 2H) 7.03 (m, 2H), 6.80 (d, J = 1.7 Hz, 2H), 6.76 (d, J = 8.9 Hz, 2H), 6.65 (dd, J = 8.8, 2.0 Hz, 2H), 6.25 (s, 2H), 4.84 (s, 2H), 4.36 (t, J = 6.8 Hz, 2H), 3.65 (s, 9H), 3.34 - 3.30 (m, 2H, overlapped by solvent signal), 2.63 (t, J = 7.1 Hz, 2H), 2.18 (t, J = 7.5 Hz, 2H), 2.10 (p, J = 6.8 Hz, 2H), 1.93 (p, J = 7.3 Hz, 2H).

HR-MS [+ scan]: calculated m/z Chemical Formula: $C_{46}H_{42}FN_5O_{10}$ 843.2928; observed 844.2995 (M+H) and 866.2830 (M+Na).

3-(prop-2-yn-1-yloxy)benzaldehyde (28)

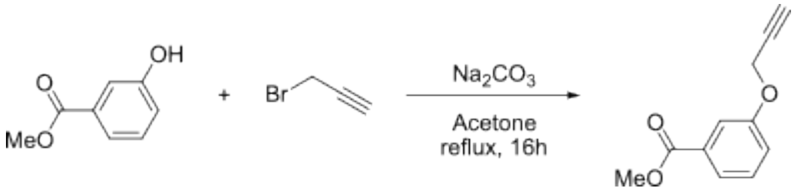

Methyl-3-hydroxybenzoate (100 mg, 0.66 mmol) and propargyl bromide (130 μL, 1.22 mmol, 1.8 eqv) were dissolved in a suspension of sodium carbonate (140 mg, 1.32 mmol, 2 eqv) in acetone (5 mL) and then stirred at reflux overnight. Once the reaction was completed, the mixture was allowed to cool to room temperature before filtering off the sodium carbonate in excess and the filtrate was concentrate under reduced pressure. The crude solution was purified by column chromatography (petrol ether/ethylacetate 8:2) to obtain the pure product as a yellow solid (104.1 mg, 0.55 mmol, 83% yield).

^{1}H NMR (500 MHz, $CDCl_3$) δ 7.71 (dt, J = 7.7, 1.3 Hz, 1H), 7.66 (dd, J = 2.6, 1.4 Hz, 1H), 7.39 (t, J = 8.0 Hz, 1H), 7.20 (ddd, J = 8.2, 2.7, 1.0 Hz, 1Hz), 4.76 (d, J = 2.5 Hz, 2H), 3.94 (s, 3H), 2.56 (t, J = 2.4 Hz, 1H).

^{13}C NMR (126 MHz, $CDCl_3$) δ 166.78, 157.49, 131.54, 129.50, 122.89, 120.26, 115.24, 78.11, 75.87, 55.99, 52.25.

MS [+ scan]: calculated m/z $C_{11}H_{10}O_3$ 190.06; observed 191.06 (M+H)

3-(prop-2-yn-1-yloxy)benzoic acid (29)

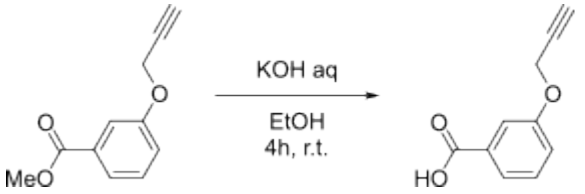

28 (200 mg, 1.06 mmol) was dissolved in a mixture of aqueous potassium hydroxide 1 N (4 mL) and ethanol (4 mL). The solution was stirred at room temperature for four hours and then quenched with the addition of extra water. The crude thus obtained was extracted with ethyl acetate and the reunited organic layers were washed with brine and water before drying them over magnesium sulphate, filtering them and evaporating the filtrate to obtain compound 15 as pure with crystalline solid (167 mg, 0.95 mmol, 90% yield).

^{1}H NMR (500 MHz, MeOD) δ, 7.66 (dt, J = 7.7, 1.2 Hz, 1H), 7.64 (dd, J = 2.7, 1.1 Hz, 1H), 7.41 (t, J = 7.8 Hz, 1H), 7.23 (ddd, J = 8.3, 2.7, 1.0 Hz, 1H), 4.80 (d, J = 2.5 Hz, 2H), 2.98 (t, J = 2.4 Hz, 1H).

^{13}C NMR (126 MHz, MeOD) δ 161.65, 133.07, 126.34, 123.51, 119.30, 81.99, 79.62, 59.31, 33.23, 26.76.

MS [+ scan]: calculated m/z $C_{10}H_8O_3$ 176.05; observed 177.05 (M+H).

3-(prop-2-yn-1-yloxy)benzoyl chloride (30)

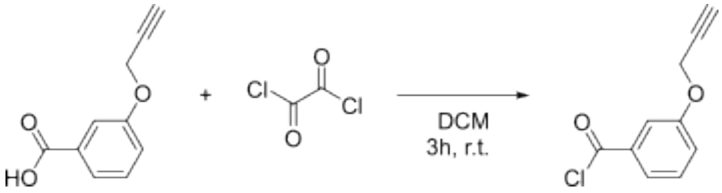

29 (100 mg, 0.64 mmol) was dissolved in dichloromethane (5 mL) in a dry round bottom flask and cooled to 0°C under inert atmosphere. Oxalyl chloride (61 µL, 073 mmol, 1.1 eqv) and a single drop of dimethylformamide were added dropwise to the mixture. The solution was stirred at 0°C for one hour and then it was allowed to warm up to room temperature and the reaction was continued for two more hours. Once the reaction was complete, the crude mixture was evaporated under reduced pressure to remove the excess of oxalyl chloride and obtain **30** as a pure brownish liquid (118.3 mg ,0.61 mmol, 95% yield).

^{1}H NMR (400 MHz, $CDCl_3$) δ 7.80 (dt, J = 7.7, 1.3 Hz, 1H), 7.74 (m, 1H), 7.44 (t, J = 8.0 Hz, 1H), 7.29 (ddd, J = 8.3, 2.7, 1.0 Hz, 1H), 4.79 (d, J = 2.4 Hz, 2H), 2.58 (t, J = 2.4 Hz, 1H).

^{13}C NMR (101 MHz, $CDCl_3$) δ 171.27, 157.55, 130.58, 129.65, 123.55, 121.23, 115.69, 76.37, 76.00, 56.02.

N-(5-(4-methoxyphenyl)-1-methyl-1H-imidazol-2-yl)-3-(prop-2-yn-1 yloxy)benzamide (31)

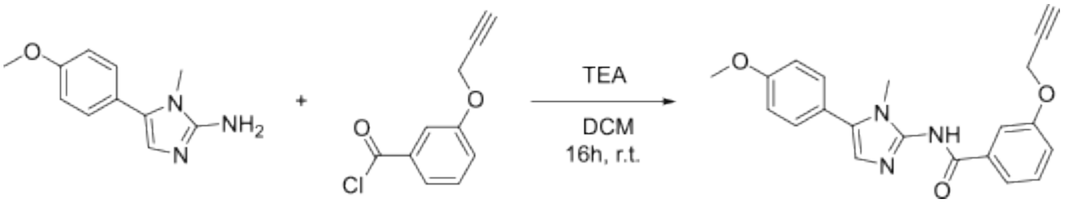

25 (40 mg, 0.2 mmol) was dissolved in dichloromethane (2 mL) with the addition of triethylamine (56 µL, 0.4 mmol, 2 eqv) at 0°C. **30** (39 mg, 0.2 mmol, 1 eqv) was added to the solution and the mixture was left stirring at room temperature overnight. Once completed, the reaction mixture was quenched with the addition of methanol and then evaporated under reduced pressure. The crude residues were taken up with dichloromethane before purification by column chromatography (dichloromethane/methanol 95:5) to obtain the pure product **31** as an off-white solid (46.5 mg, 0.13 mmol, 63% yield).

^{1}H NMR (500 MHz, $CDCl_3$) δ 7.78 (d, J = 7.1 Hz, 1H), 7.73 (s, 1H), 7.41 (m, 2H), 7.35 (d, J = 7.3 Hz, 1H), 7.12 (ddd, J = 8.1, 2.4, 1.0 Hz, 1H), 7.02 (d, J = 8.4 Hz, 2H), 6.87 (d, J = 8.9 Hz, 1H), 4.77 (d, J = 2.3 Hz, 2H), 3.88 (s, 3H), 3.59 (s, 3H), 2.56 (t, J = 2.2 Hz, 1H).

MS [+ scan]: calculated m/z $C_{21}H_{19}N_3O_3$ 361.14; observed 362.14 (M+H).

5-(4-methoxyphenyl)-1-methyl-N-(3-(prop-2-yn-1-yloxy)benzyl)-1H-imidazol-2-amine (32)

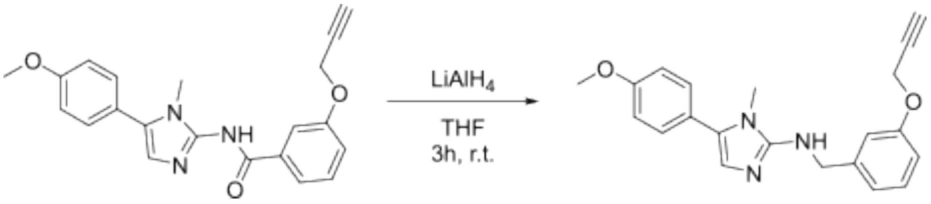

31 (50 mg, 0.14 mmol) was dissolved in tetrahydrofuran (2 mL) at 0°C before adding lithium aluminium hydride 4M in diethyl ether (40 µL, 0.75 mmol, 5.4 eqv). The mixture was stirred for three hours allowing it to warm up to room temperature. After the completion of the reaction, excess lithium aluminium hydride was quenched following the Fieser methodology, so the solution was cooled to 0°C and water was added dropwise (29 µL) followed by an aliquot of 15% NaOH aqueous solution (29 µL). The mixture was then stirred for 15 minutes before one more millilitre of water and magnesium sulphate were added. The suspension thus created was allowed to warm up to room temperature and was kept stirring until the complete formation of magnesium sulphate rock-like solids that were filtered off. The filtrate was then extracted with dichloromethane, dried over magnesium sulphate and filtered again. The crude filtrate was concentrated under reduced pressure and purified by column chromatography (dichloromethane/methanol 95:5) to obtain the pure product **32** (14 mg, 0.04 mmol, 29%)

^{1}H NMR (400 MHz, MeOD) δ 7.30 (dt, J = 8.9, 2.2 Hz, 2H), 7.24 (t, J = 8.1, 1H), 7.07 (d, J = 9.0 Hz, 1H), 6.99 (m, 3H), 6.82 (ddd, J = 8.2, 2.5, 1.1 Hz, 1H), 6.53 (s, 1H), 4.48 (s, 2H), 3.88 (d, J = 5.5 Hz, 2H), 3.83 (s, 3H), 3.56 (bs, 1H), 3.36 (s, 3H).

MS [+ scan]: calculated m/z $C_{21}H_{21}N_3O_2$ 347.16; observed 348.27 (M+H).

N-(3-((1-(17-(2-((dimethylamino)methyl)-4,5-dihydro-1H-imidazol-1-yl)-3,6,9,12,15-pentaoxaheptadecyl)-1H-1,2,3-triazol-4-yl)methoxy)benzyl)-5-(4-methoxyphenyl)-1-methyl-1H-imidazol-2-amine (PINAD-1.2)

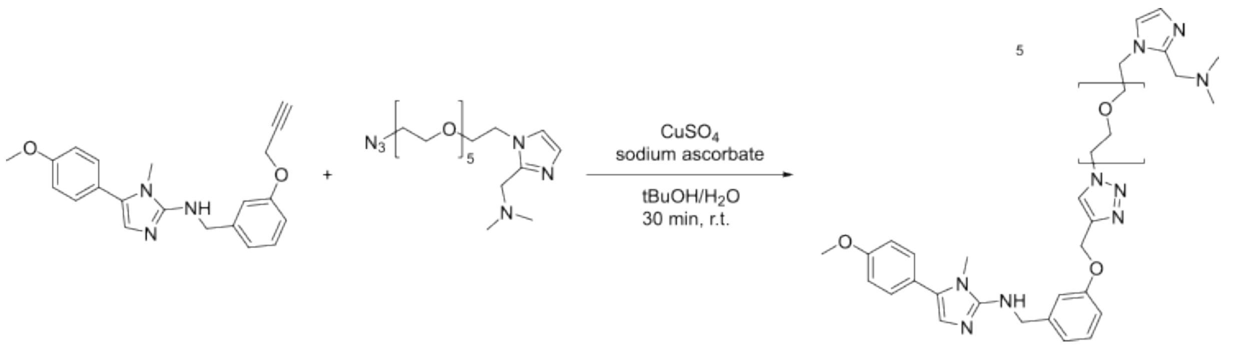

32 (10 mg, 0.029 mmol), **14A** (12.2 mg, 0.029 mmol, 1 eqv) and copper sulphate (1 mg, 0.005 mmol, 0.2 eqv) were dissolved in a 1:1 mixture of water and t-butyl alcohol (1 mL). Sodium ascorbate (30 mg, 0.15 mmol, 5.2 eqv) was added and the mixture was stirred at room temperature for 30 minutes. The resulting solution was then filtered and purified by HPLC to obtain a pale yellow oil (0.9 mg, 1.18 μmol, 6.2% yield).

^{1}H NMR (400 MHz, MeOD) δ 7.80 (s, 1H), 7.45 (d, J = 1.5 Hz, 1H), 7.35 (d, J = 8.8 Hz, 2H), 7.29 - 7.22 (m, 3H), 7.20 (bs, 1H), 7.07 (d, J = 8.7, 2H), 6.91 (d, J = 8.2 Hz, 1H), 6.85 (d, J = 7.7 Hz, 1H), 6.80 (bs, 1H), 5.51 (s, 2H), 4.53 (t, J = 4.7 Hz, 2H), 4.44 – 4.40 (m, 4H), 3.87 (m, 5H), 3.81 (t, J = 4.9 Hz, 2H), 3.77 (s, 3H), 3.66 – 3.57 (m, 18H), 2.89 (s, 6H).

HR-MS [+ scan]: calculated m/z $C_{39}H_{55}N_9O_7$ 761.4231; observed 761.4226 and 762.4255 (M+H).

3',6'-dihydroxy-N-(3-(4-((3-(((5-(4-methoxyphenyl)-1-methyl-1H-imidazol-2-yl)amino)methyl)phenoxy)methyl)-1H-1,2,3-triazol-1-yl)propyl)-3-oxo-3H-spiro[isobenzofuran-1,9'-xanthene]-6-carboxamide (PINAD-1.2 FAM)

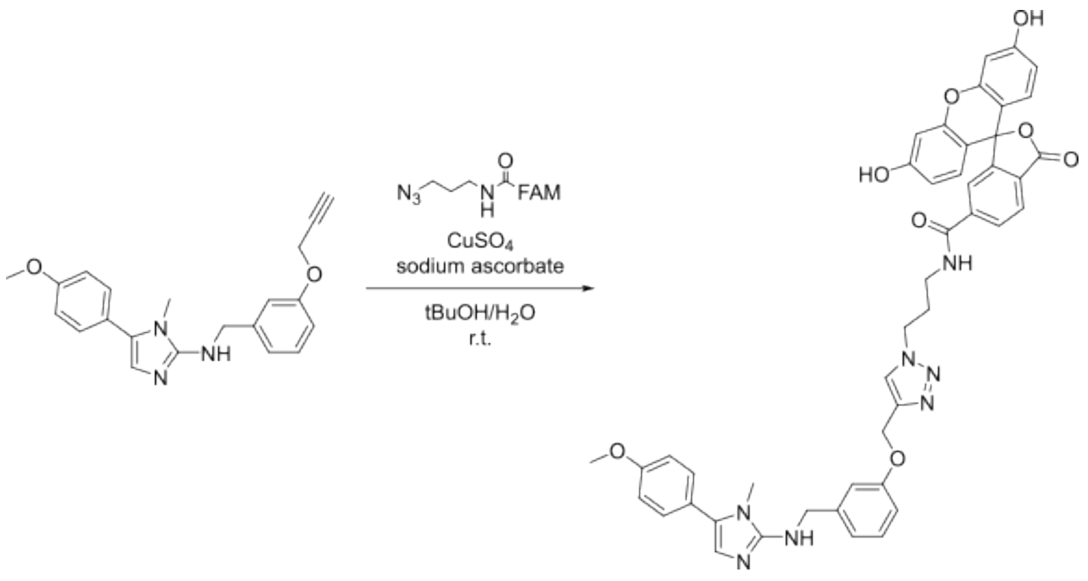

32 (10 mg, 0.029 mmol), N-(3-azidopropyl)-3',6'-dihydroxy-3-oxo-3H-spiro[isobenzofuran-1,9'-xanthene]-6-carboxamide (13.3 mg, 0.029 mmol, 1 eqv) and copper sulfate (1 mg, 0.005 mmol, 1.2 eqv) were dissolved in a 1:1 mixture of water and t-butyl alcohol (1 mL). Sodium ascorbate (6 mg, 0.03 mmol, 5.2 eqv) was added and the mixture was stirred at room temperature for 30 minutes. The resulting solution was then filtered and purified by HPLC to obtain a bright yellow oil (1.2 mg, 1.49 µmol, 5.1% yield).

^{1}H NMR (500 MHz, MeOD) δ 8.02 (s, 1H), 8.0 (m, 1H), 7.94 (s, 1H), 7.51 (s, 1H), 7.26 (d, J = 8.9 Hz, 2H), 7.18 (t, J = 8.1 Hz, 1H), 7.15 (bs, 1H), 7.00 (bs, 1H), 6.93 (d, J = 8.3 Hz, 2H), 6.88 (d, J = 8.3 Hz, 2H), 6.83 (dd, J = 8.0, 2.7 Hz, 1H), 6.66 (s, 1H), 6.57 (d, J = 2.3 Hz, 2H), 6.46 (dd, J = 8.9, 2.3 Hz, 2H), 5.39 (s, 2H), 4.43 (s, 2H), 4.36 (t, J = 6.7 Hz, 2H), 3.74 (s, 3H), 3.54 (s, 3H), 3.50 (bs, 2H), 3.07 (t, J = 1.7 Hz, 2H).

HR-MS [+ scan]: calculated m/z $C_{45}H_{39}N_7O_8$ 805.2899; observed 402.6438 (M+2) and 805.2842 (M+H).

^{1}H and ^{13}C NMR characterization spectra

Compound 1

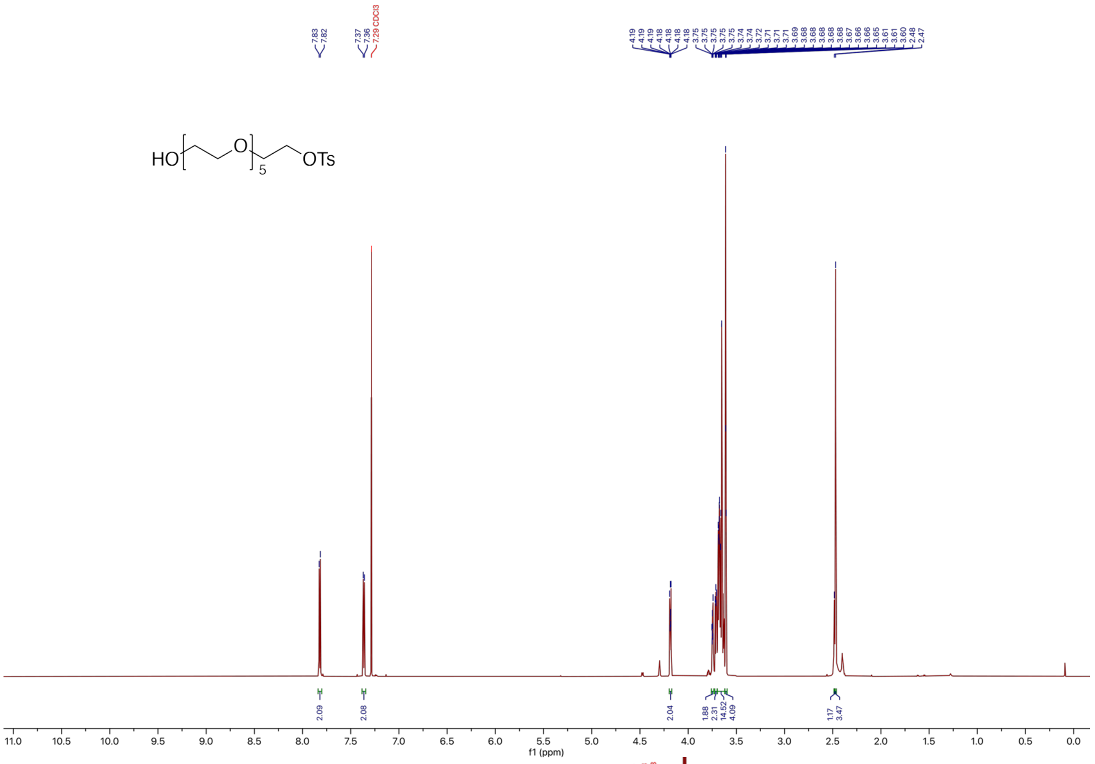

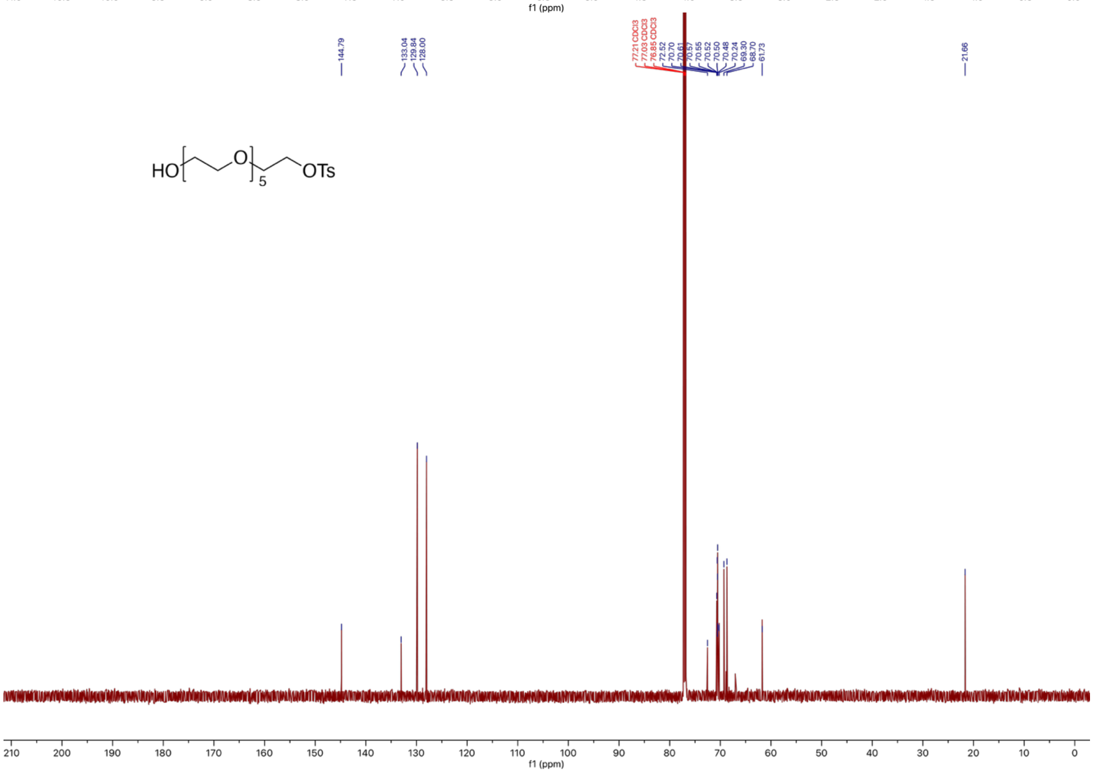

Compound 2

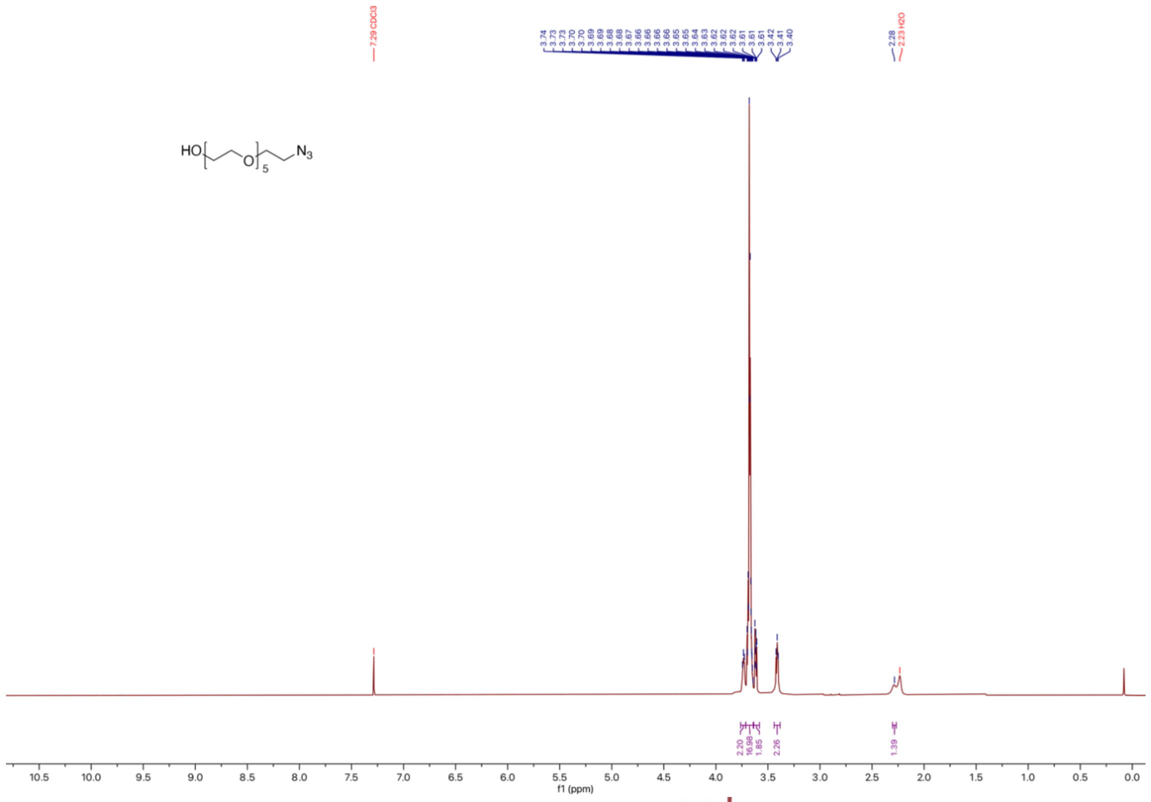

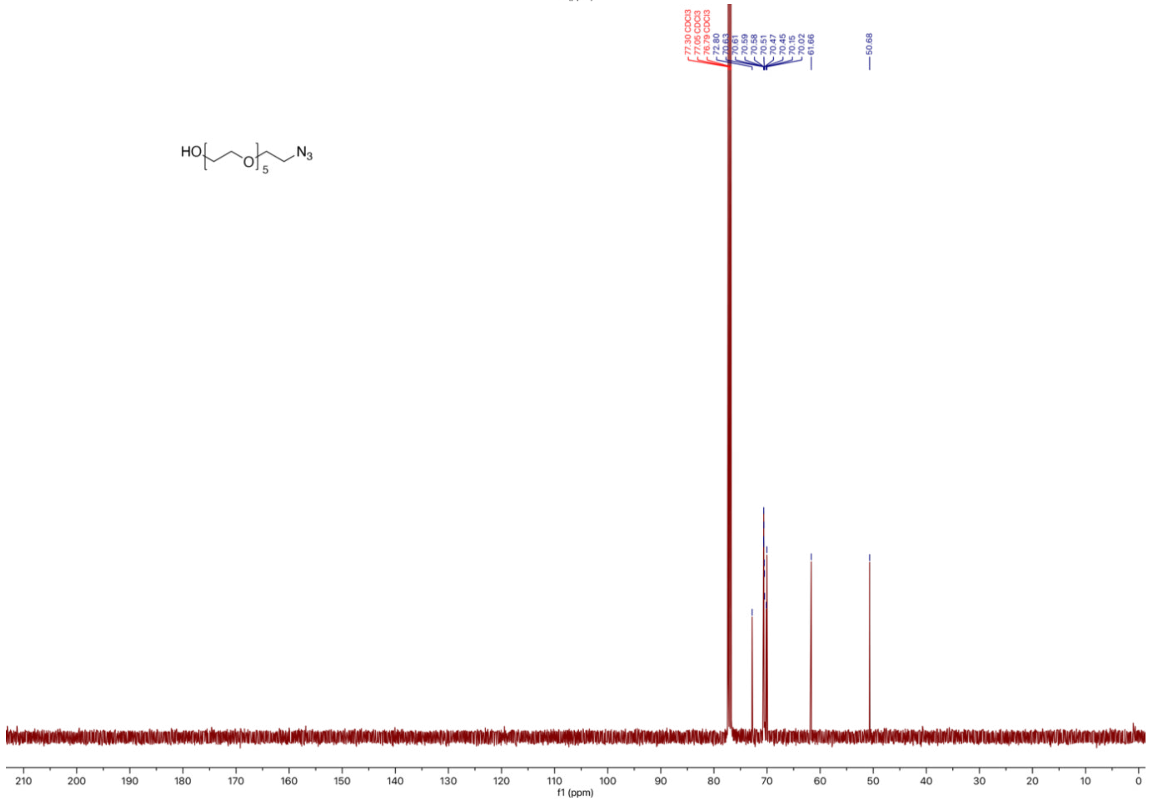

Compound 3

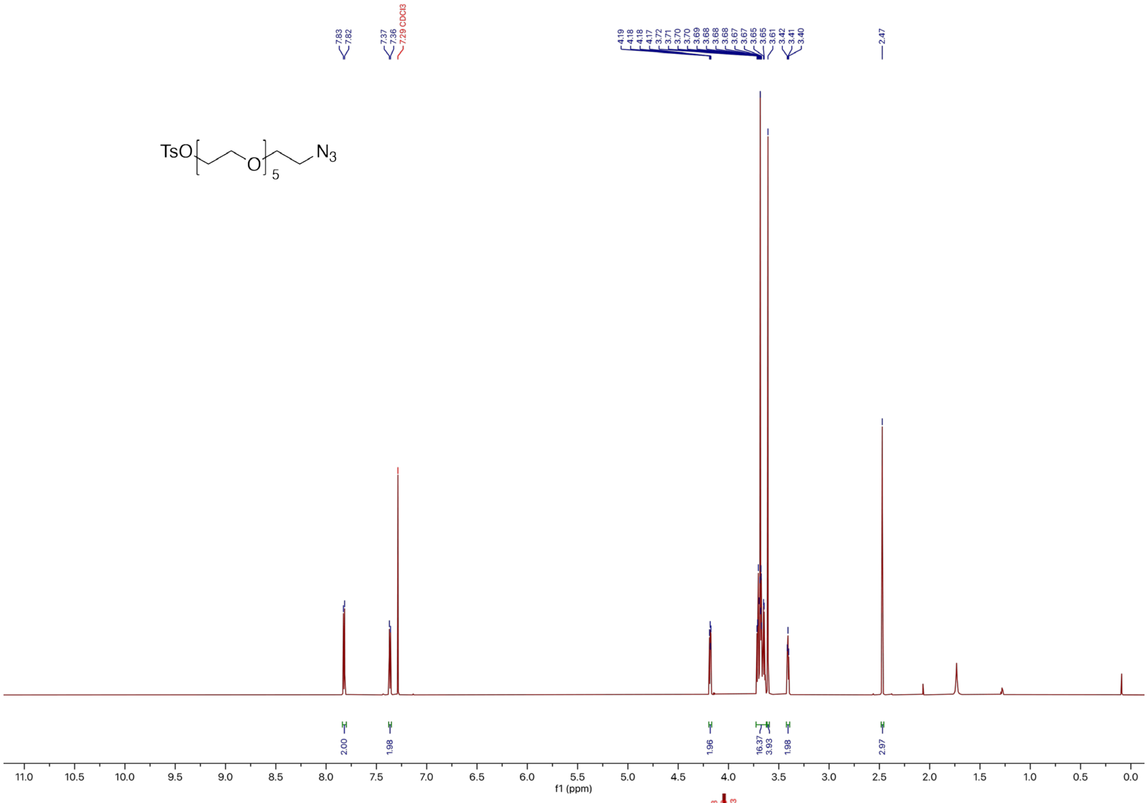

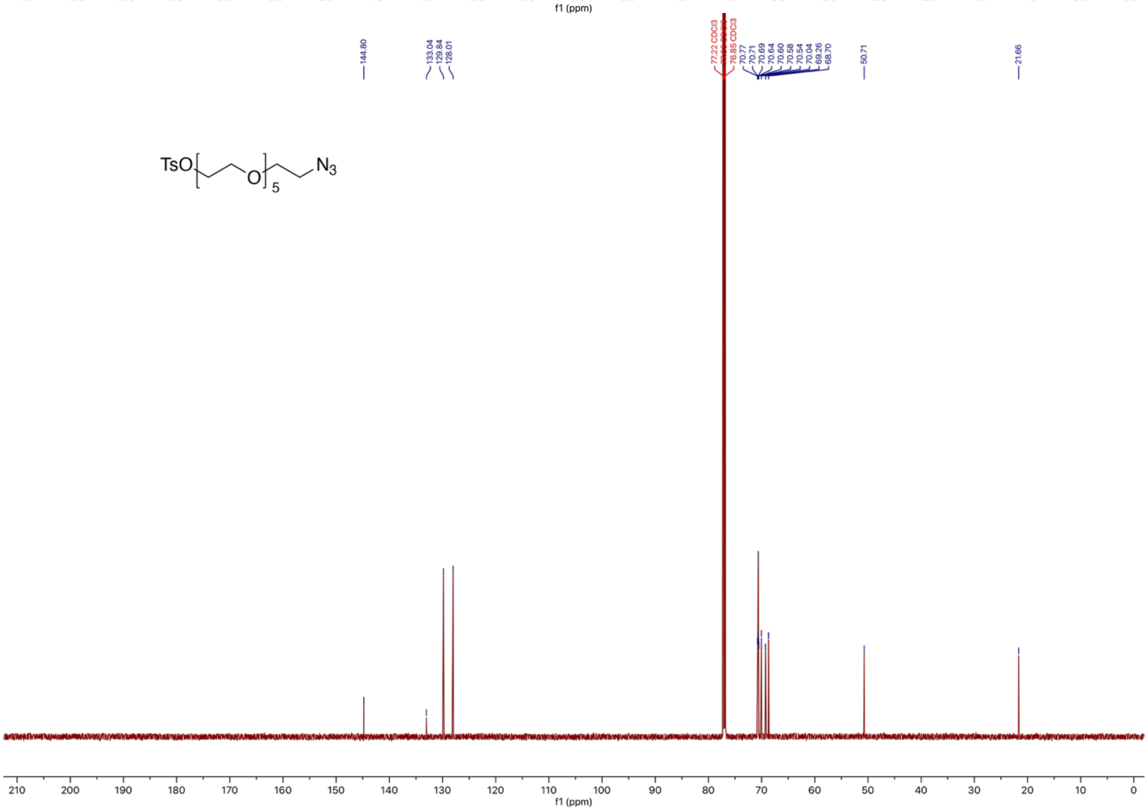

Compound 4

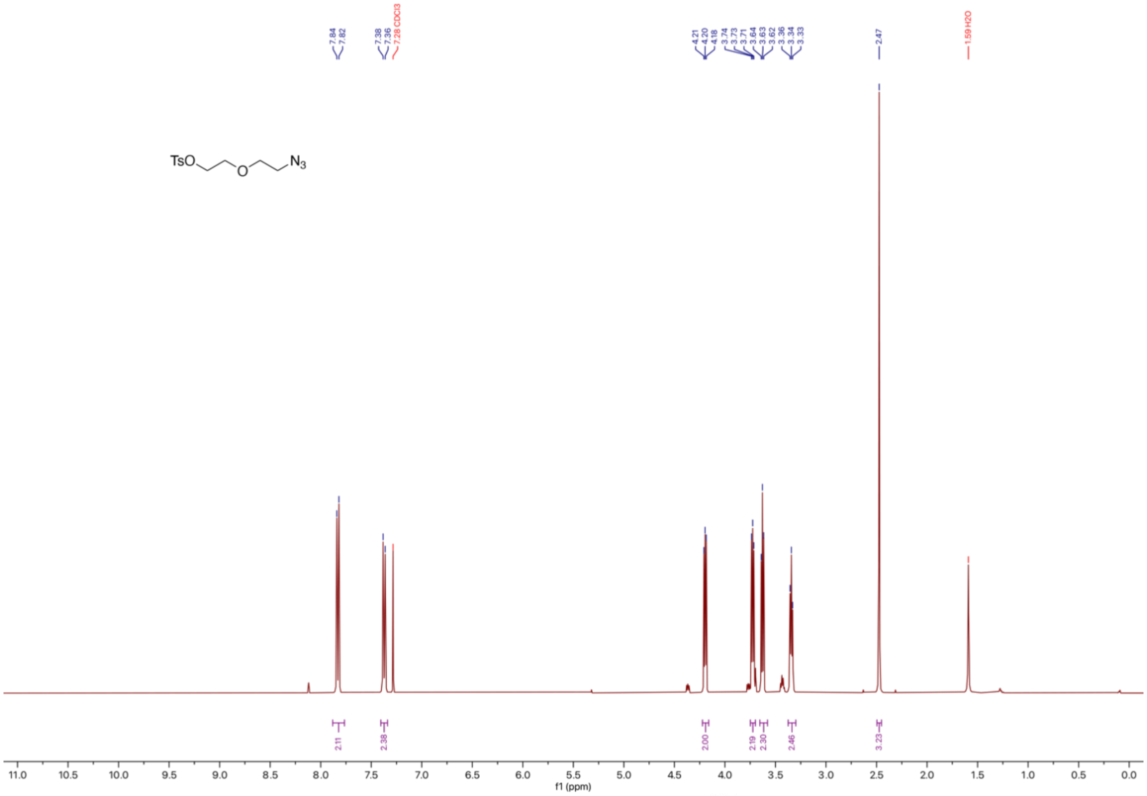

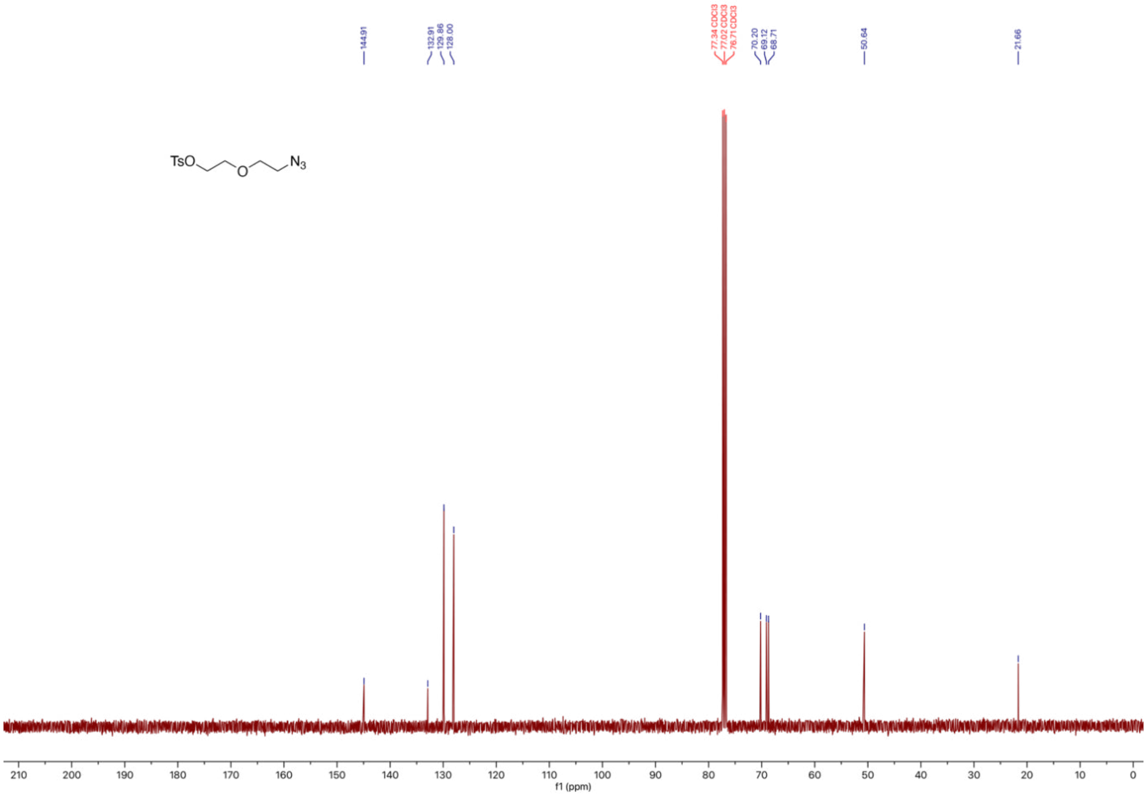

Compound 5

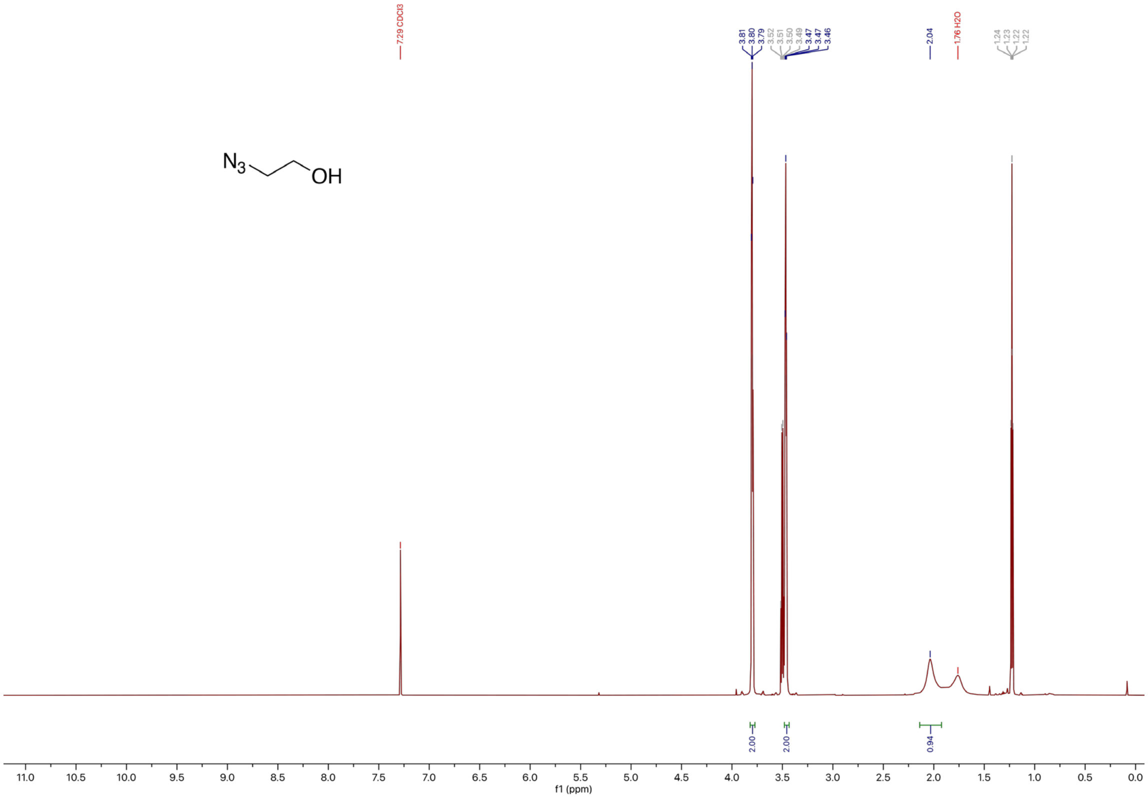

7.29 CDCl3
3.81
3.80
3.79
3.52
3.51
3.50
3.49
3.47
3.47
3.46
2.04
1.76 H2O
1.24
1.23
1.22
1.22
N3
OH
2.00
2.00
0.94
11.0
10.5
10.0
9.5
9.0
8.5
8.0
7.5
7.0
6.5
6.0
5.5
5.0
4.5
4.0
3.5
3.0
2.5
2.0
1.5
1.0
0.5
0.0
f1 (ppm)

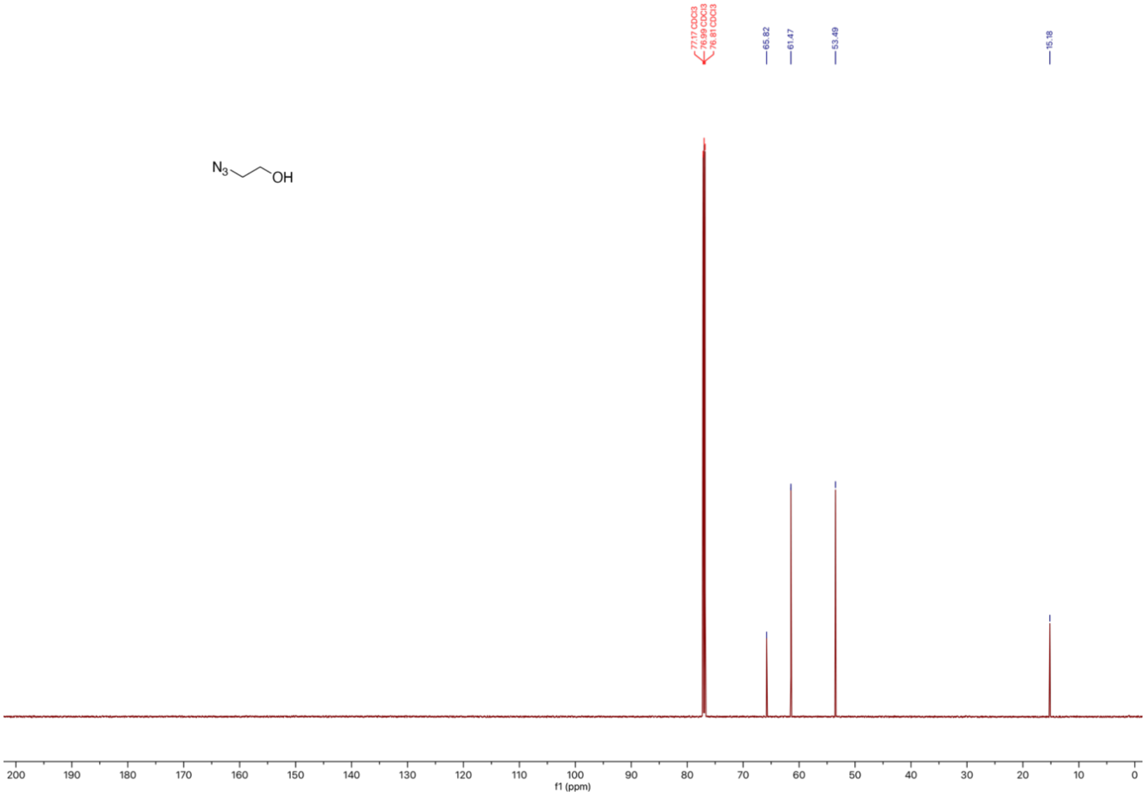

77.17 CDCl3
76.99 CDCl3
76.81 CDCl3
65.82
61.47
53.49
15.18
N3
OH
200
190
180
170
160
150
140
130
120
110
100
90
80
70
60
50
40
30
20
10
0
f1 (ppm)

Compound 6

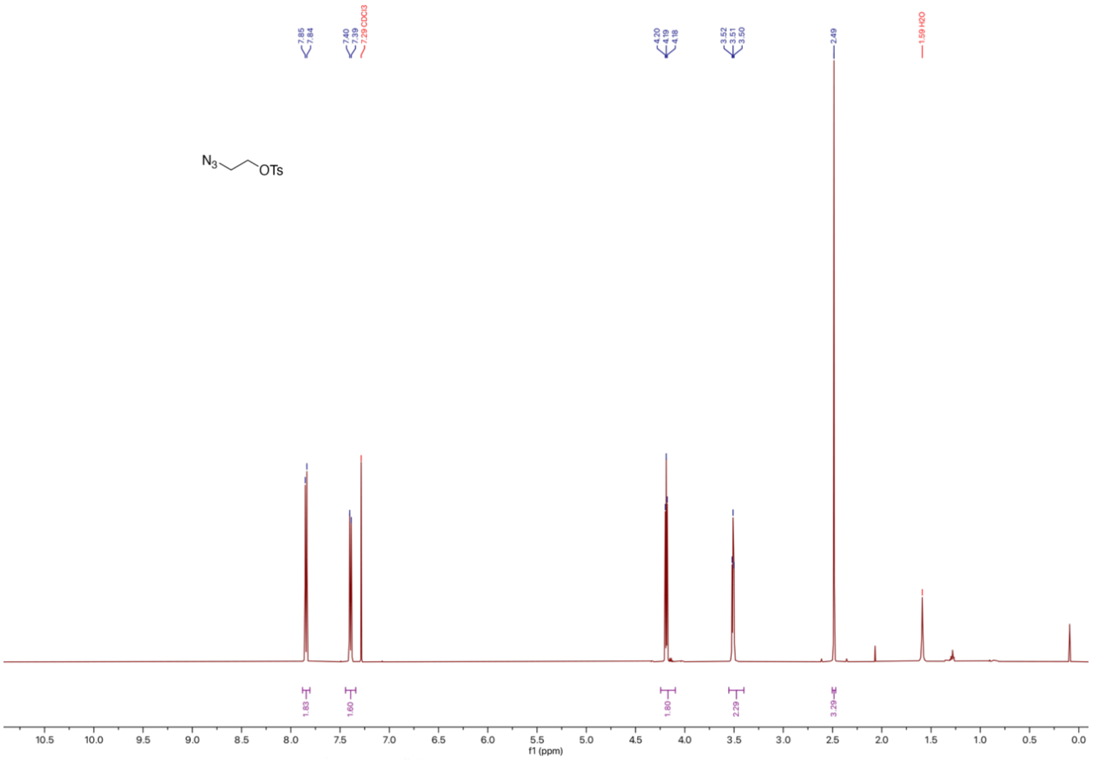

7.85
7.84
7.40
7.39
7.29 CDCl3
4.20
4.19
4.18
3.52
3.51
3.50
2.49
1.59 H2O
N3 OTs
1.83
1.60
1.80
2.29
3.29
f1 (ppm)

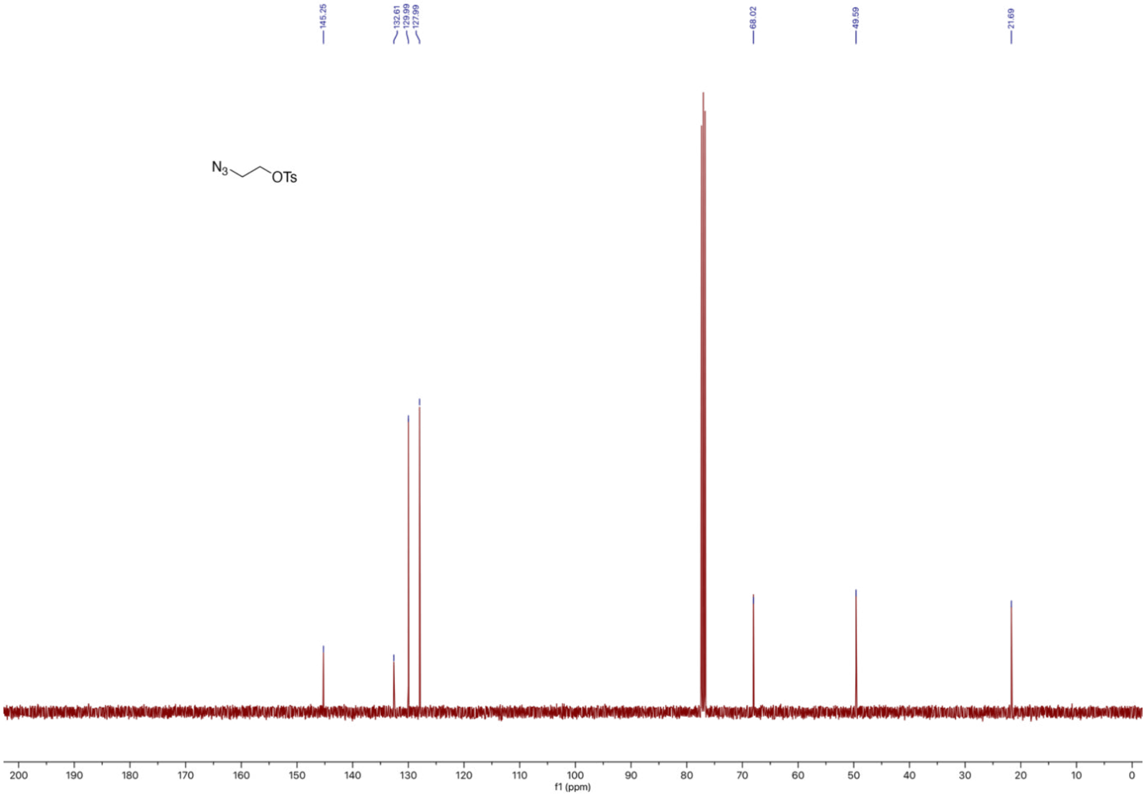

145.25
132.61
129.99
127.99
68.02
49.59
21.69
N3 OTs
f1 (ppm)

Compound 7

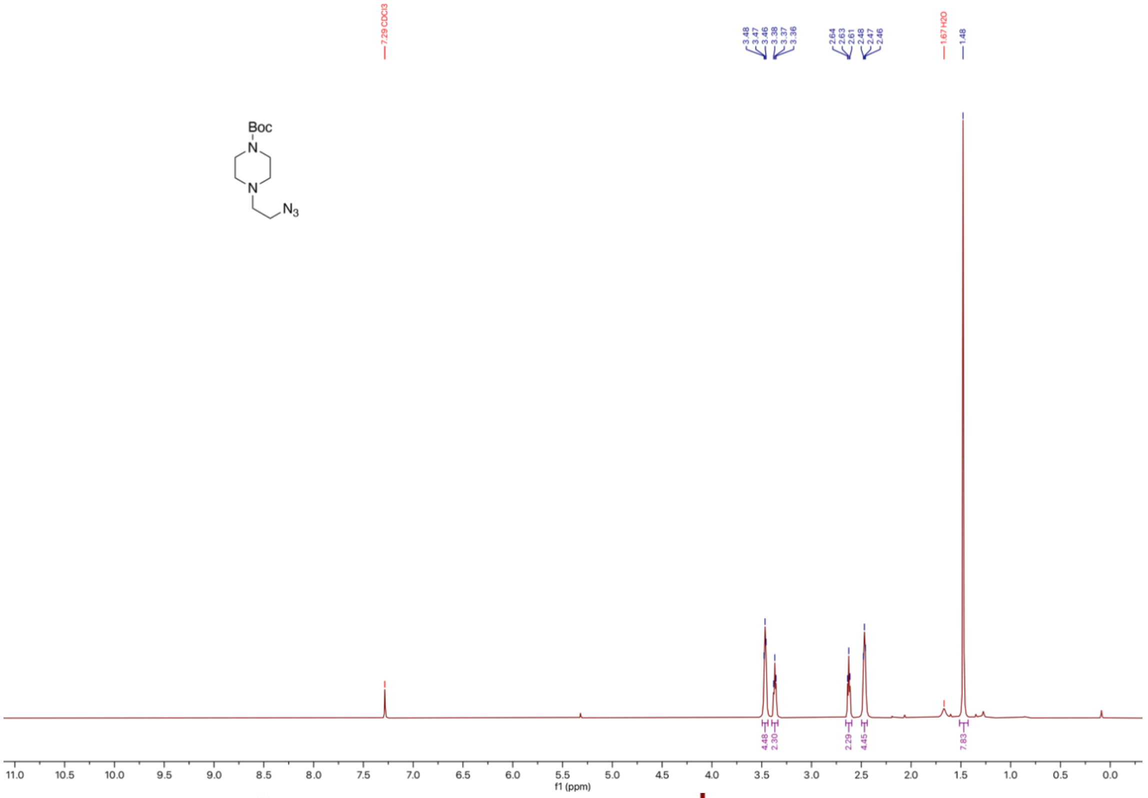

7.29 CDCl3
3.48
3.47
3.46
3.38
3.37
3.36
2.64
2.63
2.61
2.48
2.47
2.46
1.67 H2O
1.48
Boc
N
N
N3
4.48
2.30
2.29
4.45
7.83
11.0 10.5 10.0 9.5 9.0 8.5 8.0 7.5 7.0 6.5 6.0 5.5 5.0 4.5 4.0 3.5 3.0 2.5 2.0 1.5 1.0 0.5 0.0
f1 (ppm)

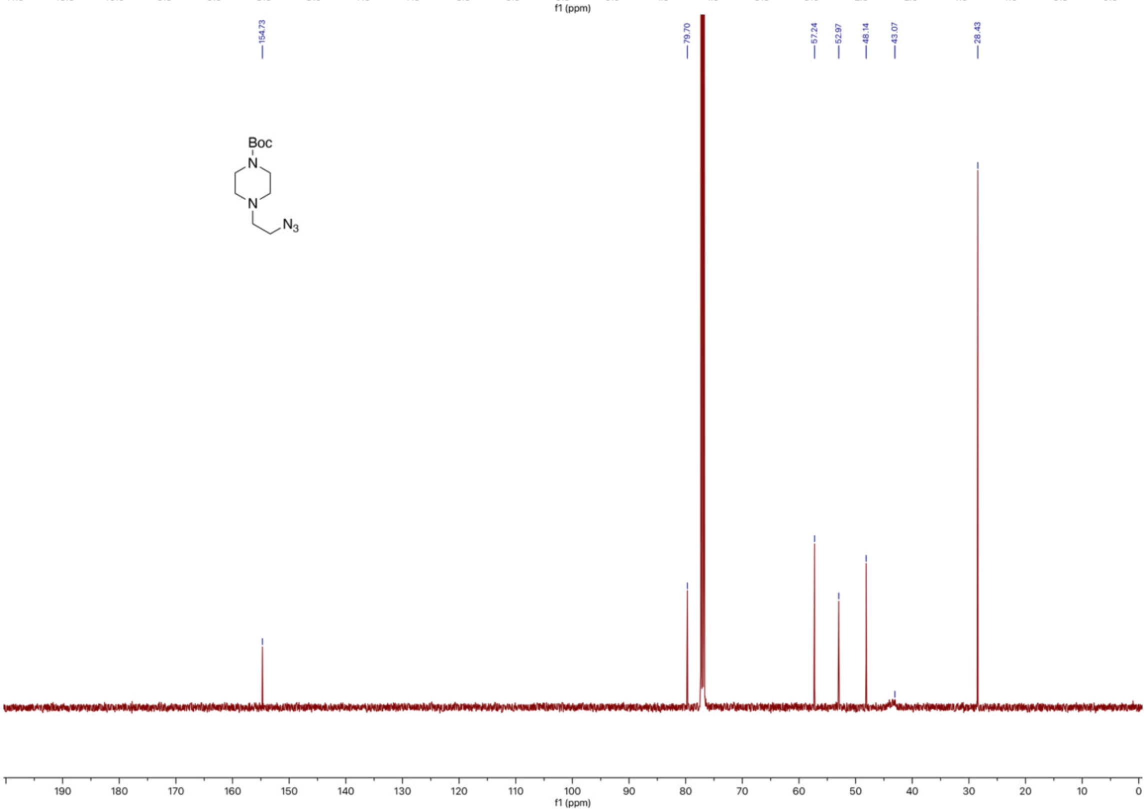

154.73
79.70
57.24
52.97
48.14
43.07
28.43
Boc
N
N
N3
190 180 170 160 150 140 130 120 110 100 90 80 70 60 50 40 30 20 10 0
f1 (ppm)

Compound 8

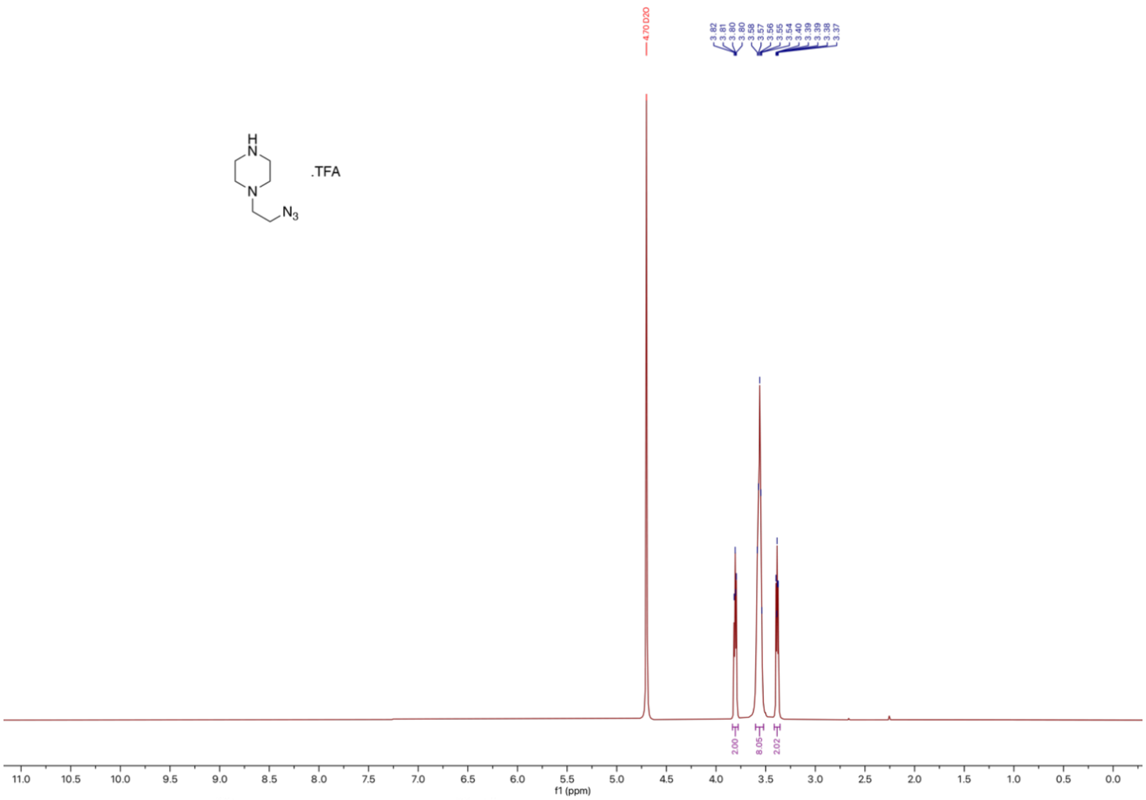
4.70 D2O
3.82
3.81
3.80
3.80
3.58
3.57
3.56
3.55
3.54
3.40
3.39
3.39
3.38
3.37
.TFA
N3
2.00
8.05
2.02
f1 (ppm)

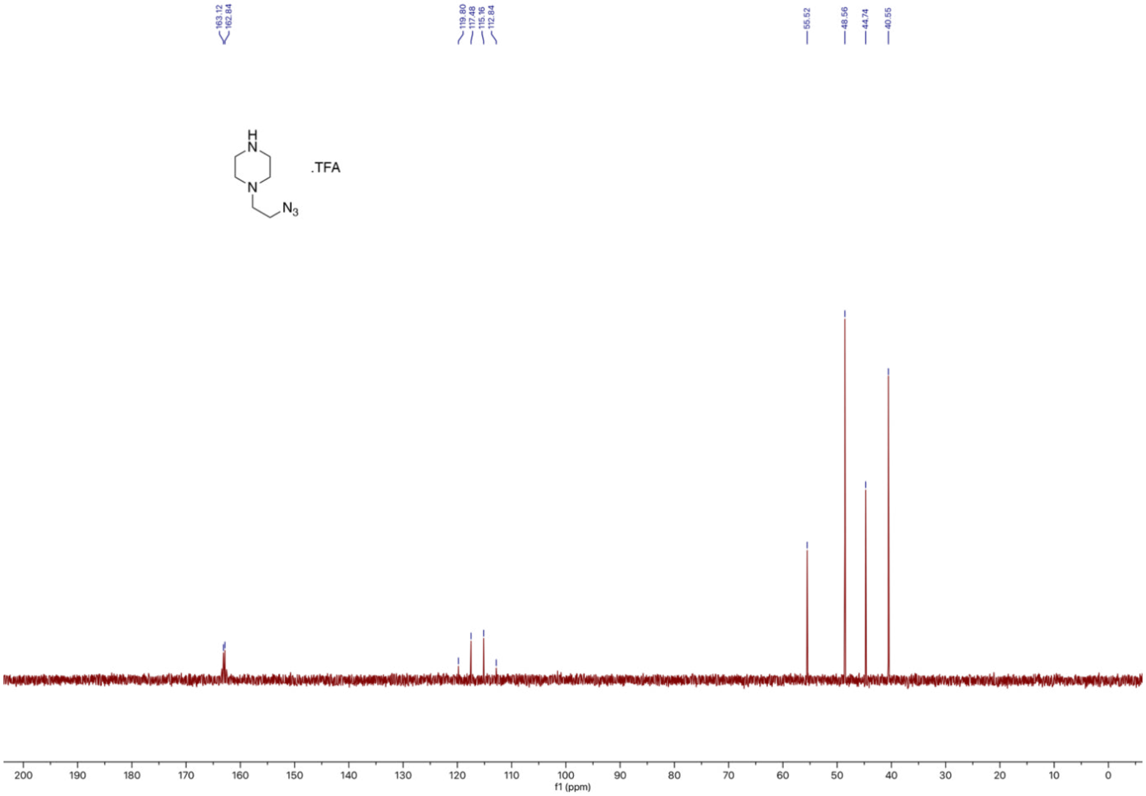
163.12
162.84
119.80
117.48
115.16
112.84
55.52
48.56
44.74
40.55
.TFA
N3
f1 (ppm)

Compound 9

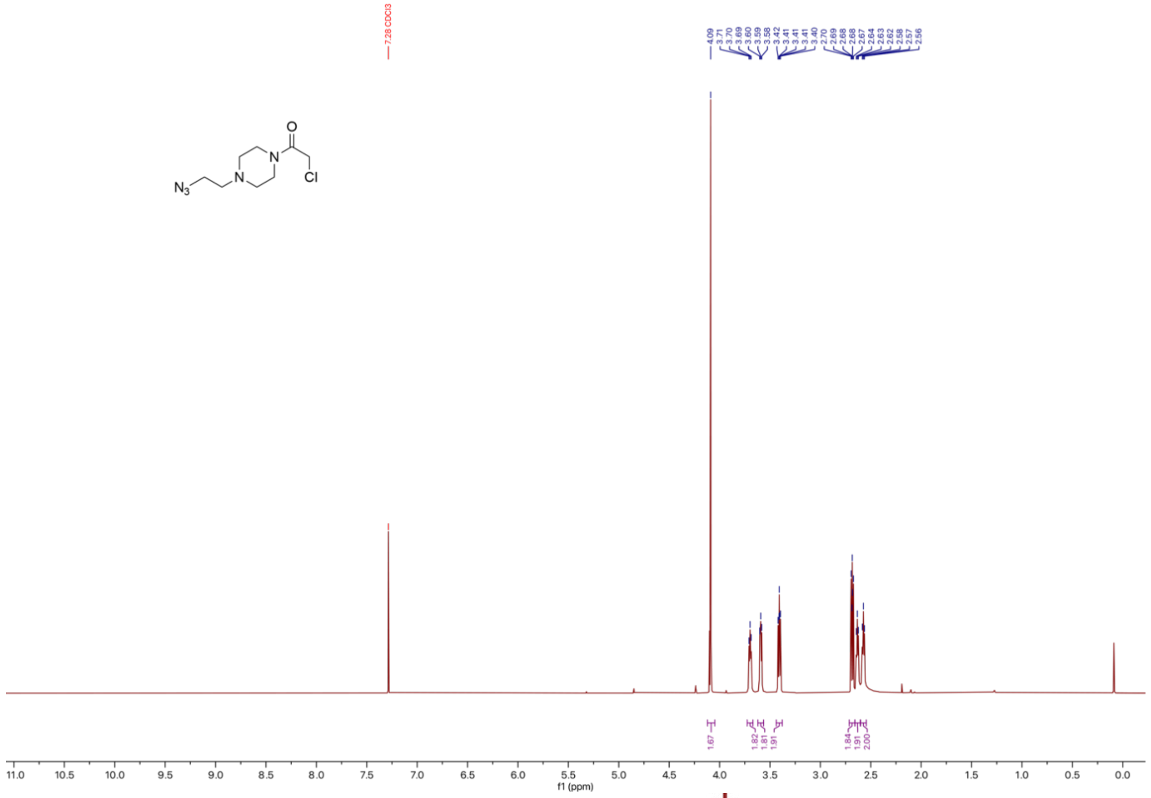
7.28 CDCl3
4.09
3.71
3.70
3.69
3.60
3.59
3.58
3.42
3.41
3.41
3.40
2.70
2.69
2.68
2.68
2.67
2.64
2.63
2.62
2.58
2.57
2.56
N3
N
N
O
Cl
1.67
1.82
1.81
1.91
1.84
1.91
2.00
f1 (ppm)

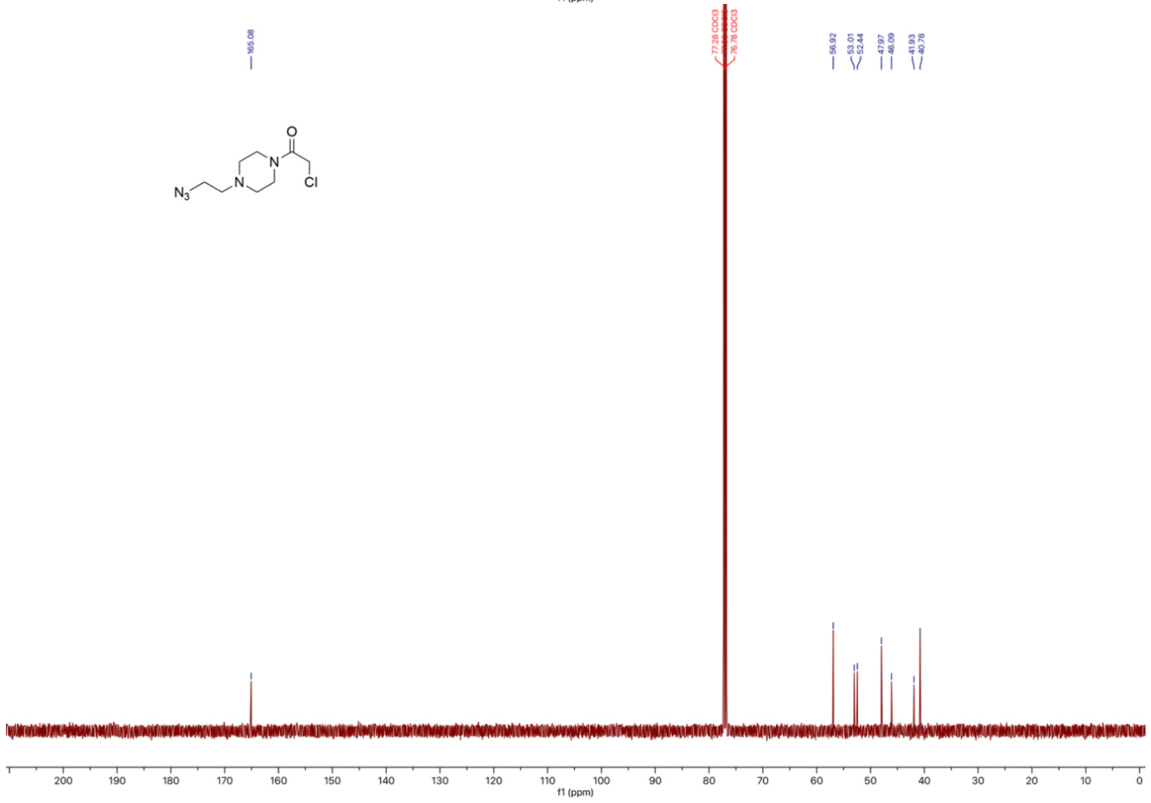
165.08
77.28 CDCl3
76.78 CDCl3
56.92
53.01
52.44
47.97
46.09
41.93
40.78
N3
N
N
O
Cl
f1 (ppm)

Compound 10

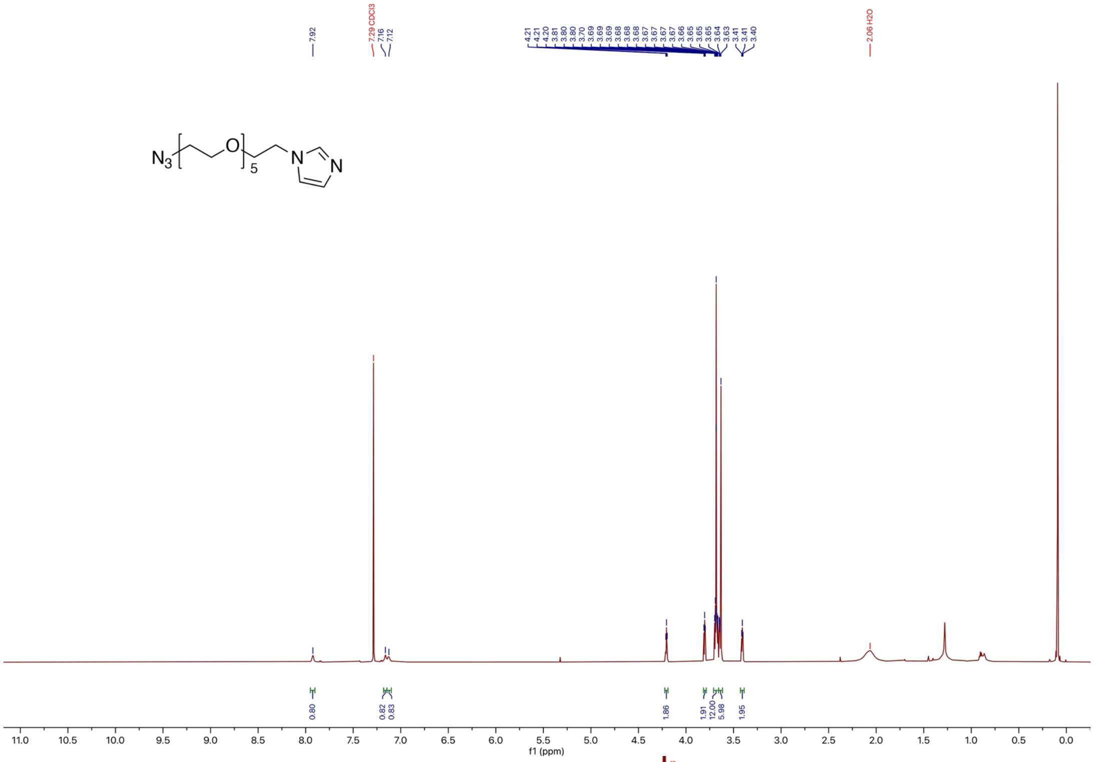

7.92
7.29 CDCl3
7.16
7.12
4.21
4.21
4.20
3.81
3.80
3.80
3.70
3.69
3.69
3.69
3.68
3.68
3.68
3.67
3.67
3.67
3.66
3.65
3.65
3.64
3.63
3.41
3.41
3.40
2.06 H2O
N3
O
5
N
N
0.80
0.82
0.83
1.86
1.91
12.00
5.98
1.95
11.0 10.5 10.0 9.5 9.0 8.5 8.0 7.5 7.0 6.5 6.0 5.5 5.0 4.5 4.0 3.5 3.0 2.5 2.0 1.5 1.0 0.5 0.0
f1 (ppm)

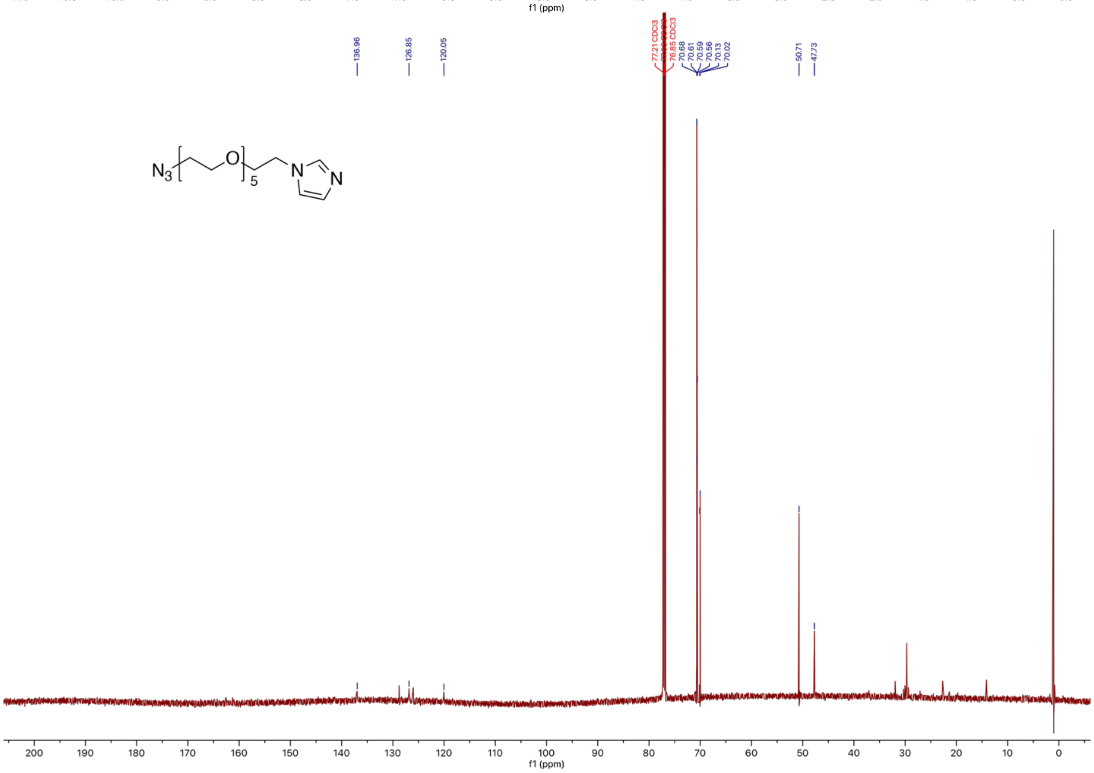

136.96
126.85
120.05
77.21 CDCl3
76.85 CDCl3
70.68
70.61
70.59
70.56
70.13
70.02
50.71
47.73
N3
O
5
N
N
200 190 180 170 160 150 140 130 120 110 100 90 80 70 60 50 40 30 20 10 0
f1 (ppm)

Compound 11

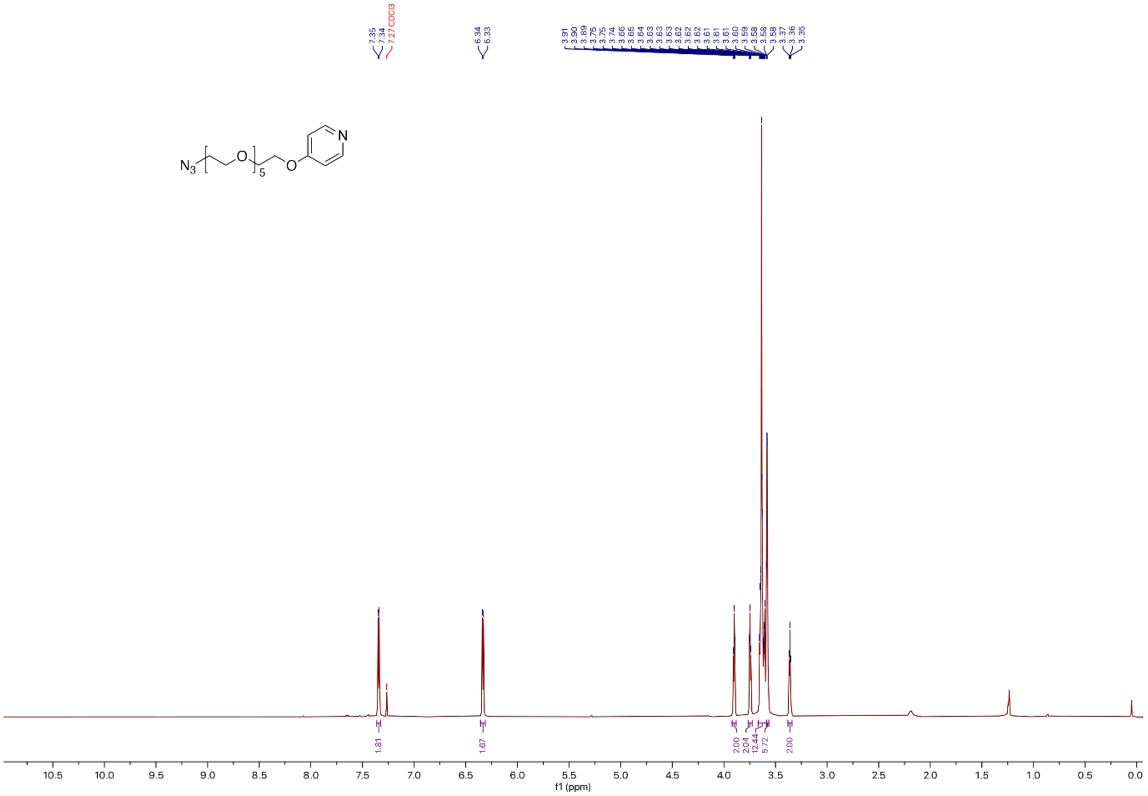

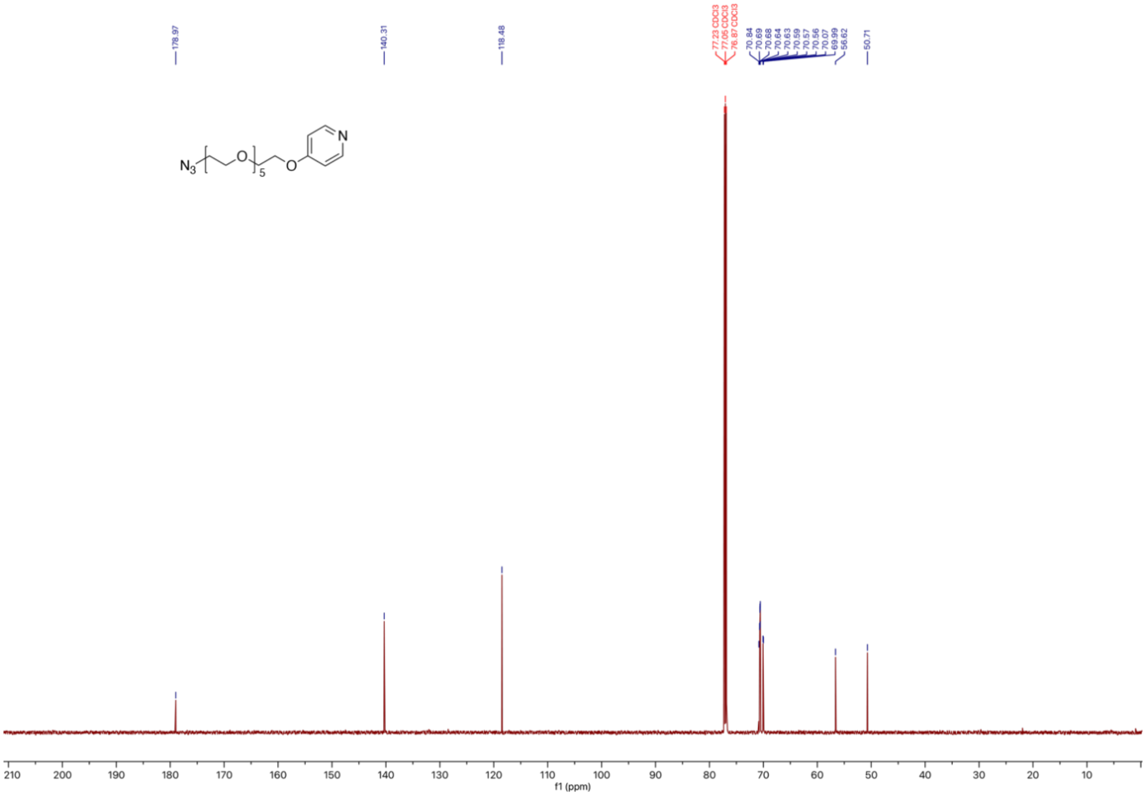

Compound 12

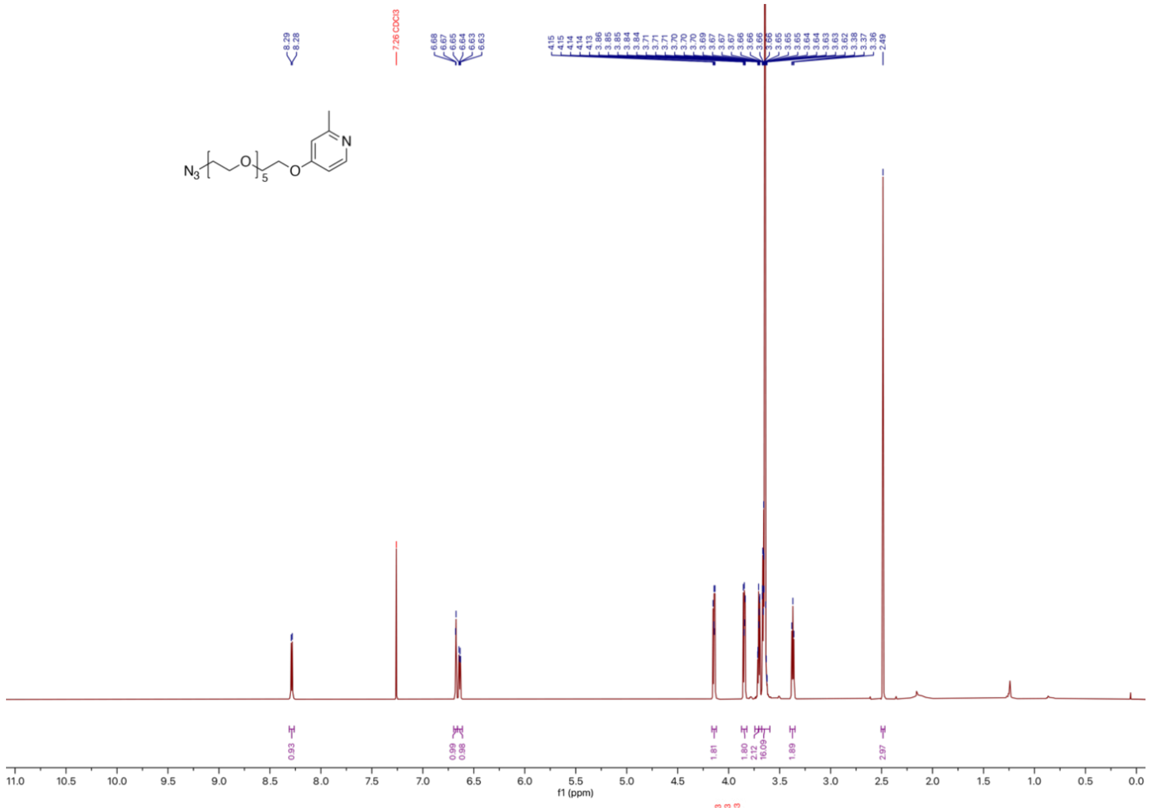

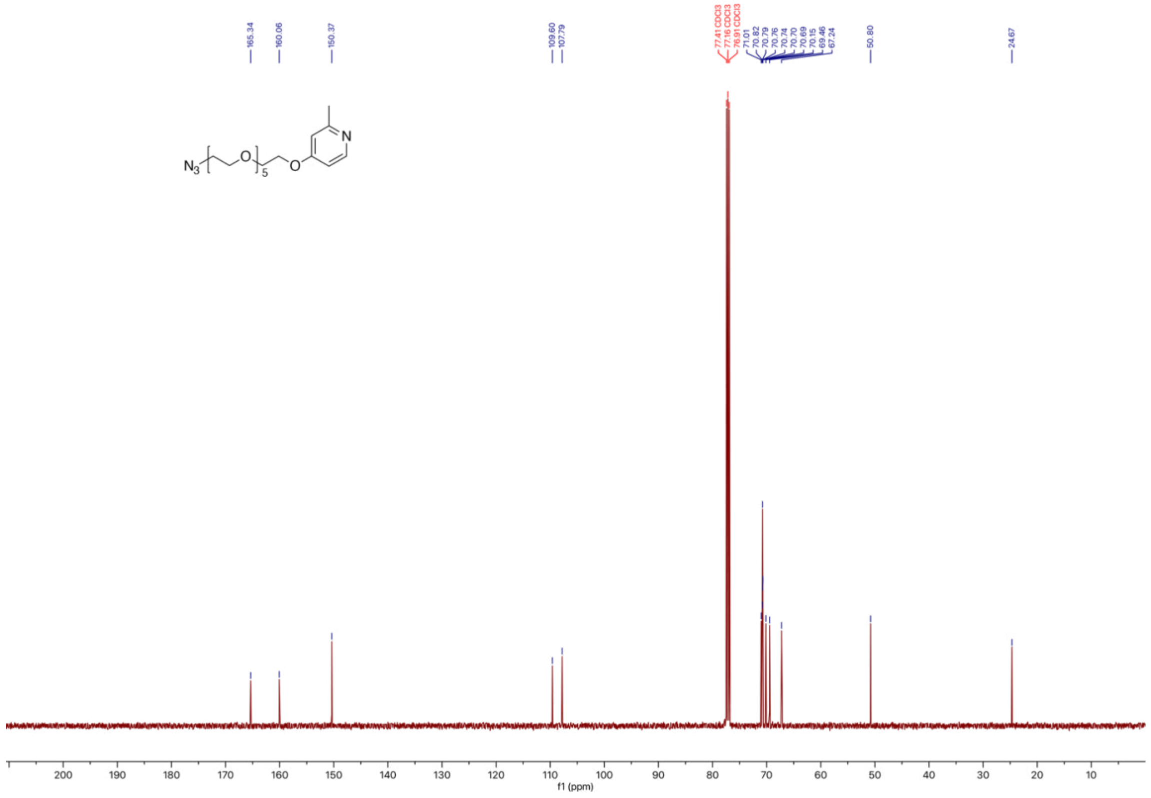

Compound 13

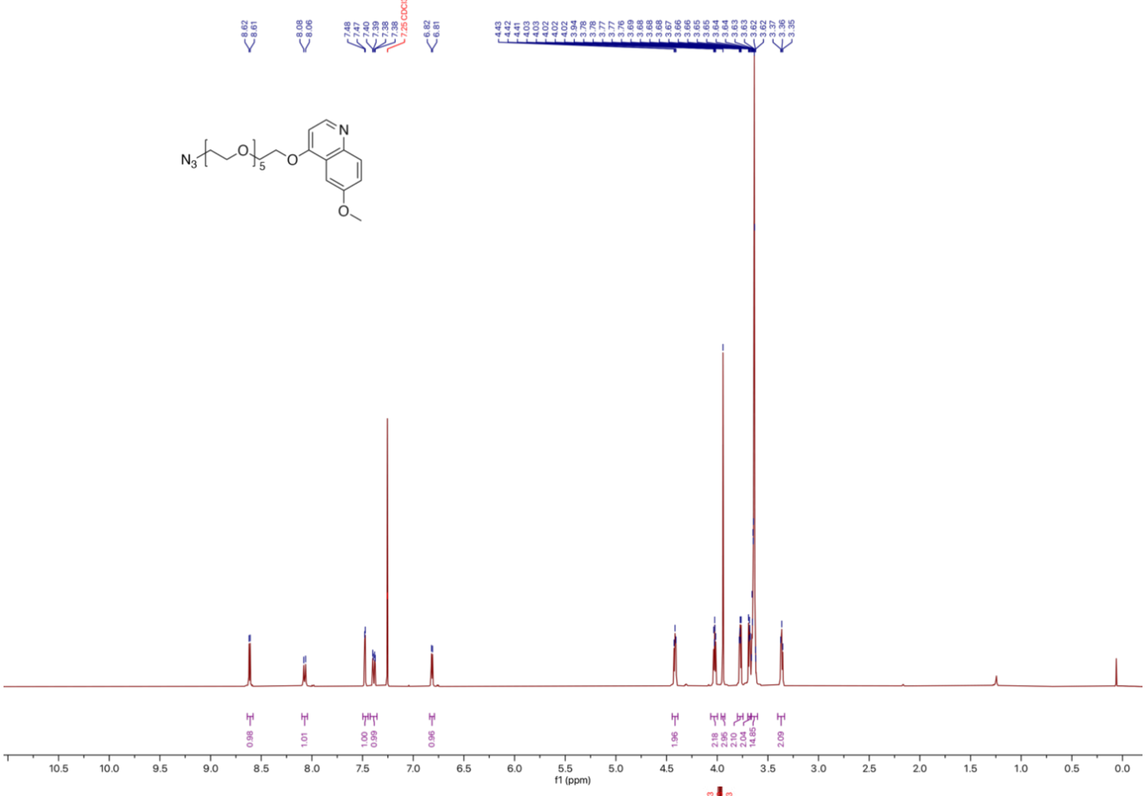

8.62
8.61
8.08
8.06
7.48
7.47
7.40
7.39
7.38
7.38
7.25 CDCl3
6.82
6.81
4.43
4.42
4.41
4.03
4.03
4.02
4.02
3.94
3.78
3.78
3.77
3.76
3.69
3.68
3.68
3.68
3.67
3.66
3.66
3.65
3.65
3.64
3.64
3.63
3.63
3.62
3.62
3.37
3.36
3.35
0.98
1.01
1.00
0.99
0.96
1.96
2.18
2.95
2.10
2.04
14.85
2.09
f1 (ppm)

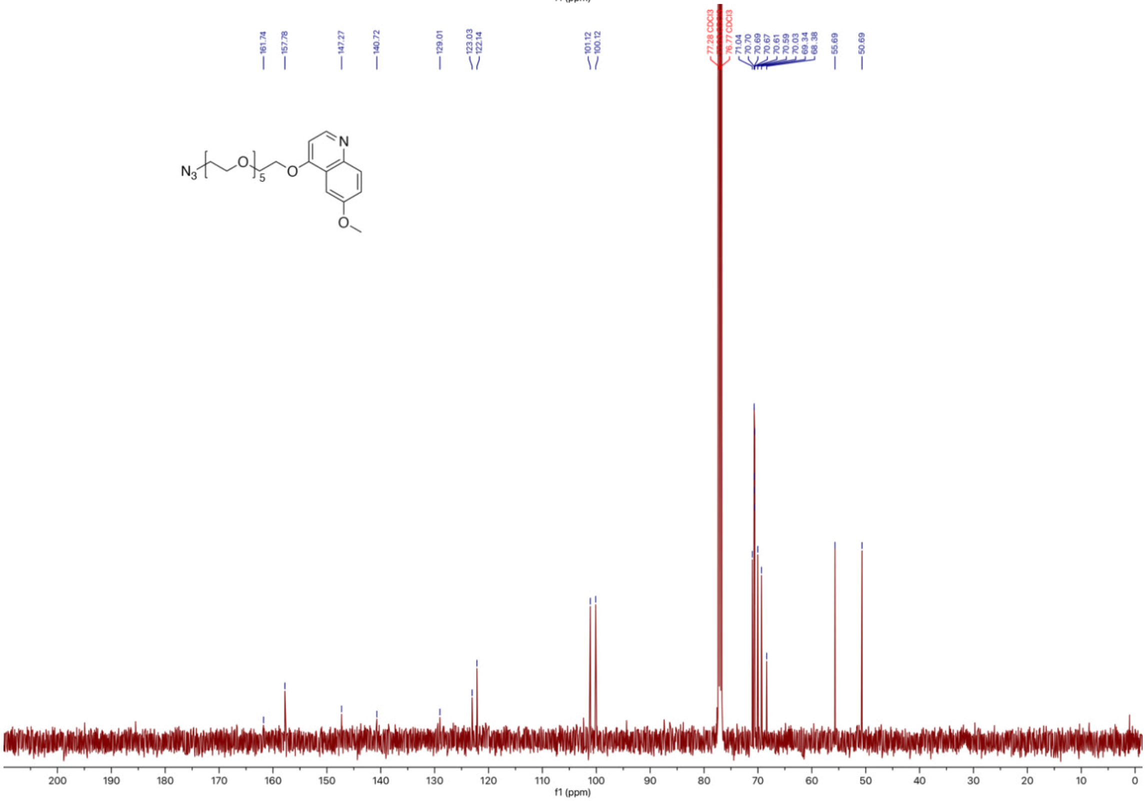

161.74
157.78
147.27
140.72
129.01
123.03
122.14
101.12
100.12
77.28 CDCl3
76.77 CDCl3
71.04
70.70
70.69
70.67
70.61
70.59
70.03
69.34
68.38
55.69
50.69
f1 (ppm)

Compound 14A

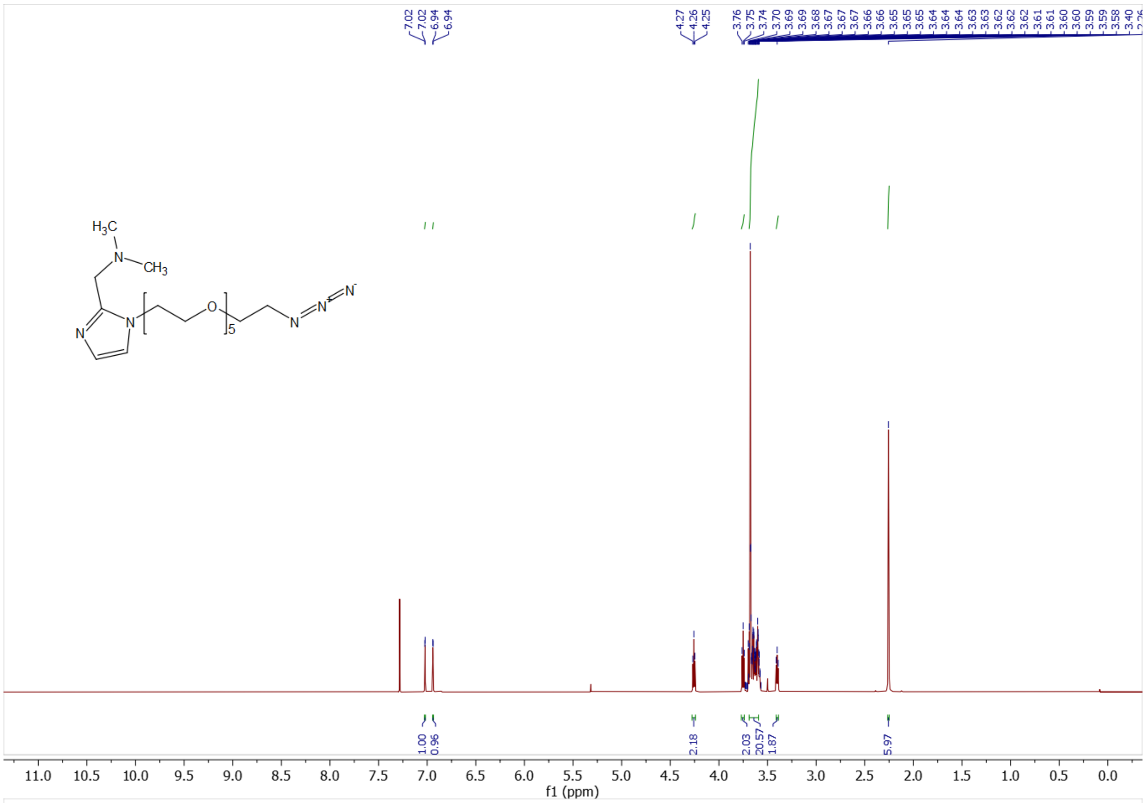

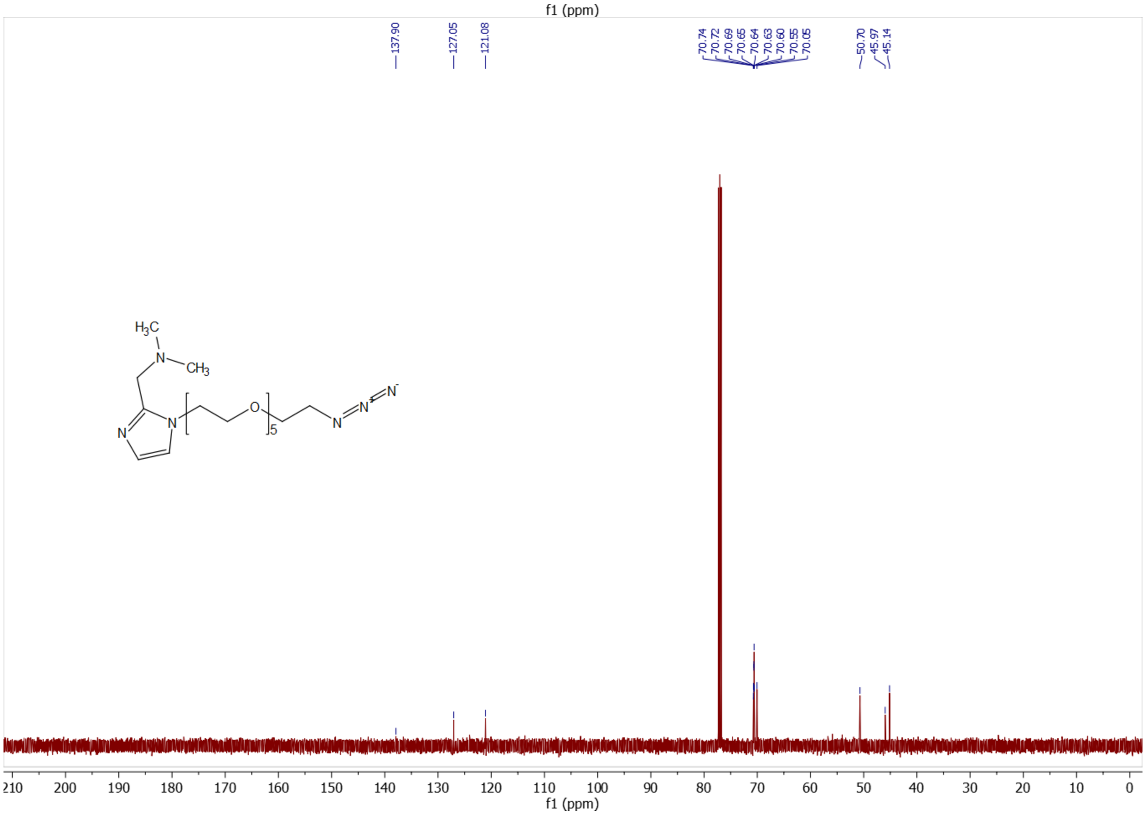

Compound 14B

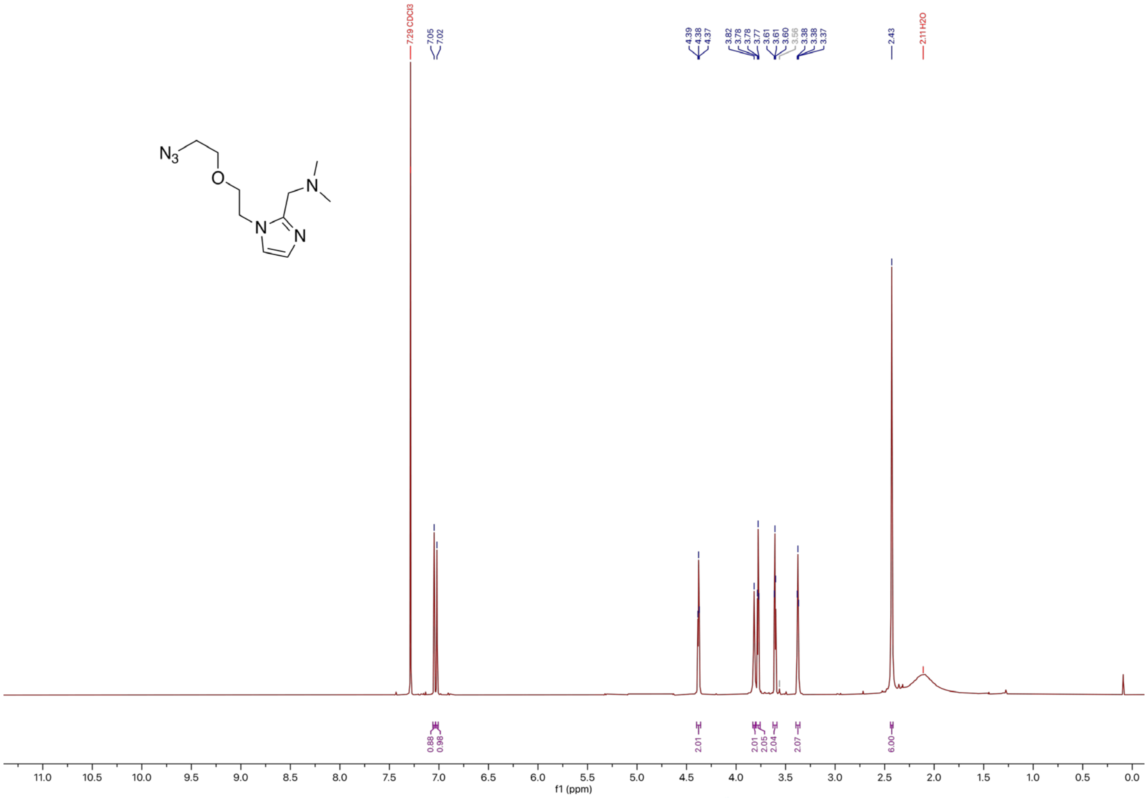

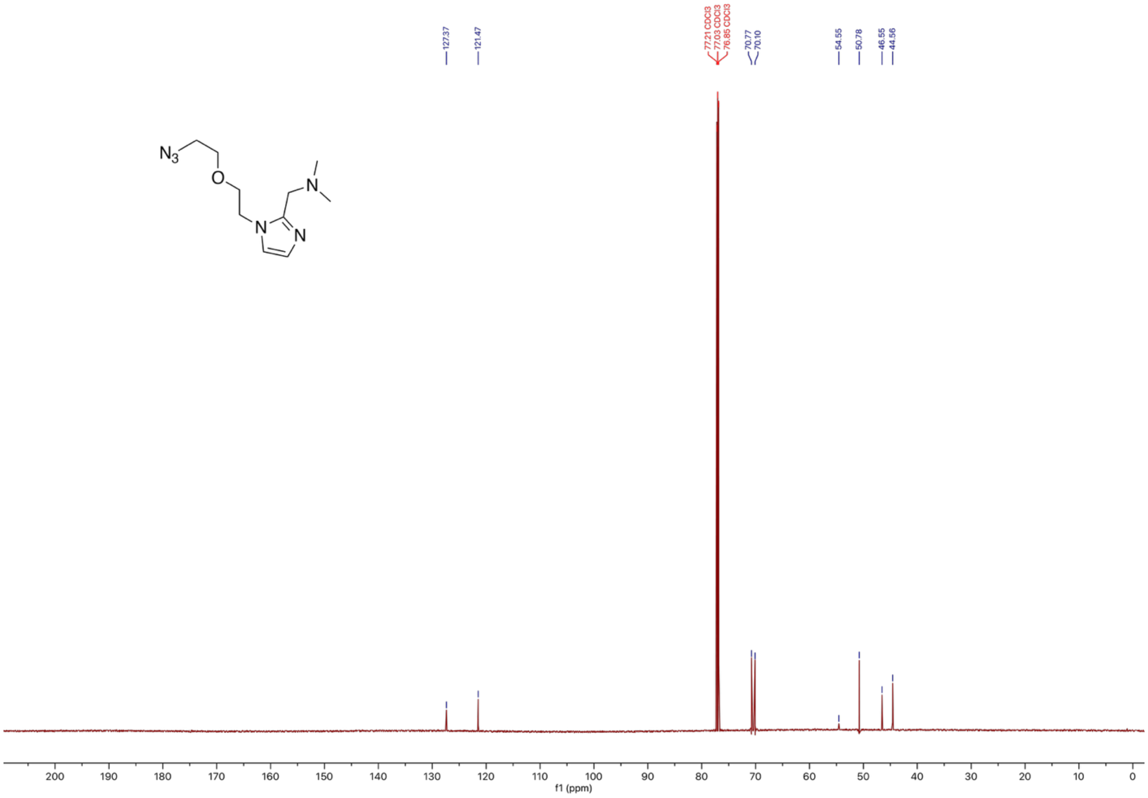

Compound 14C

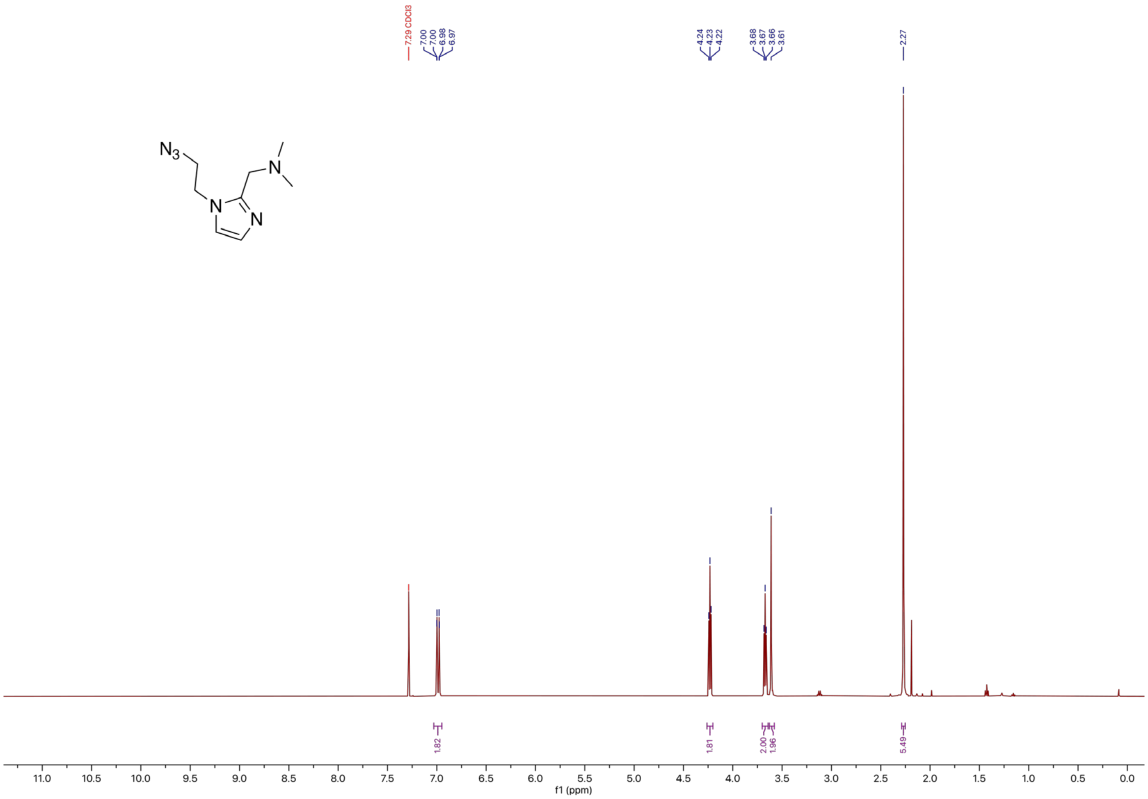

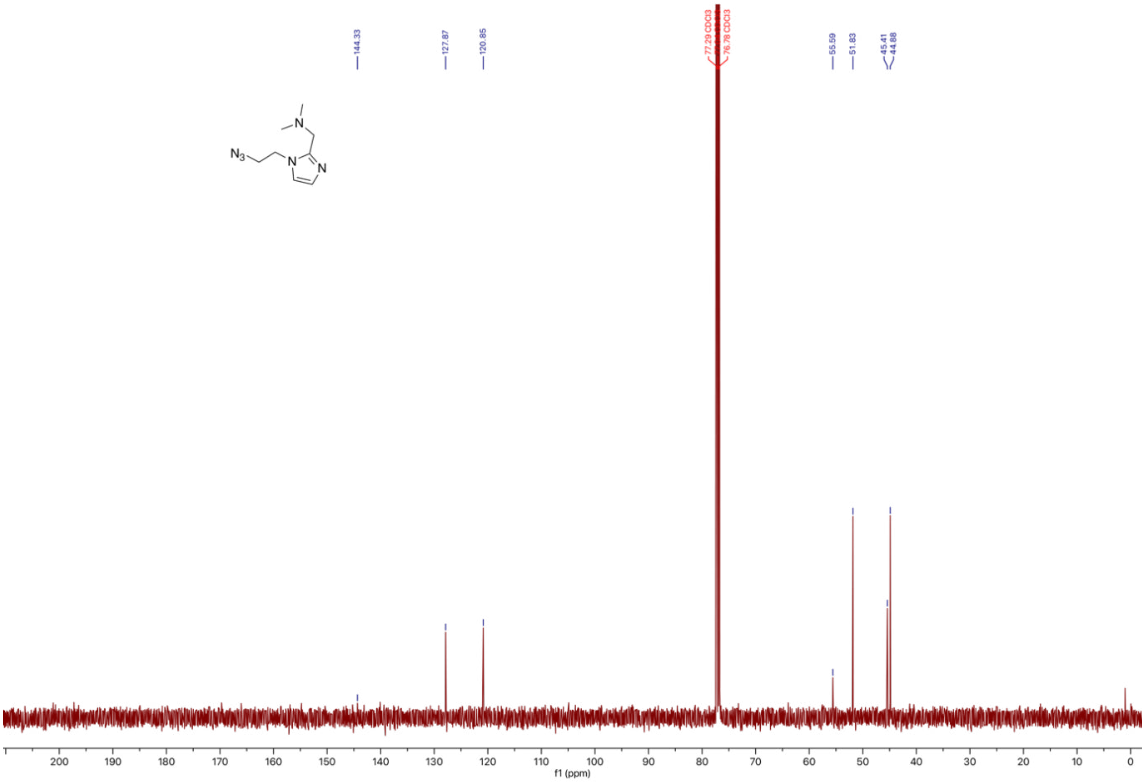

Compound 14D

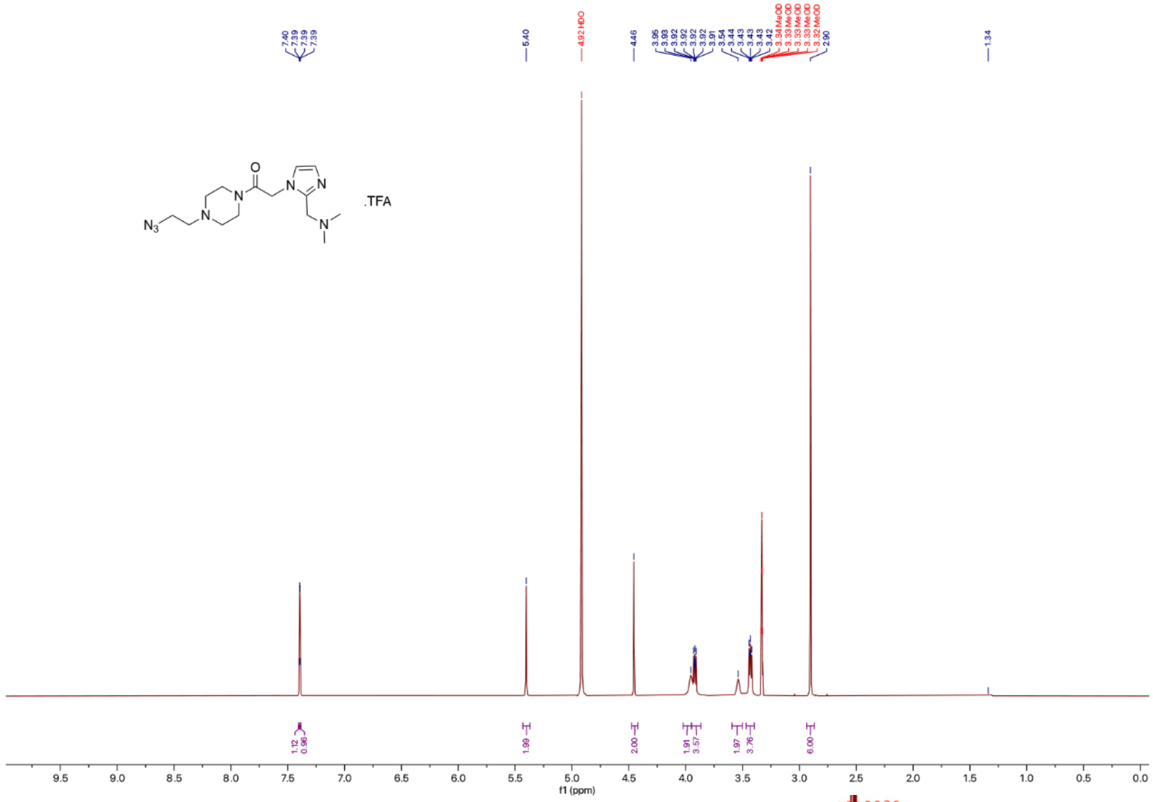

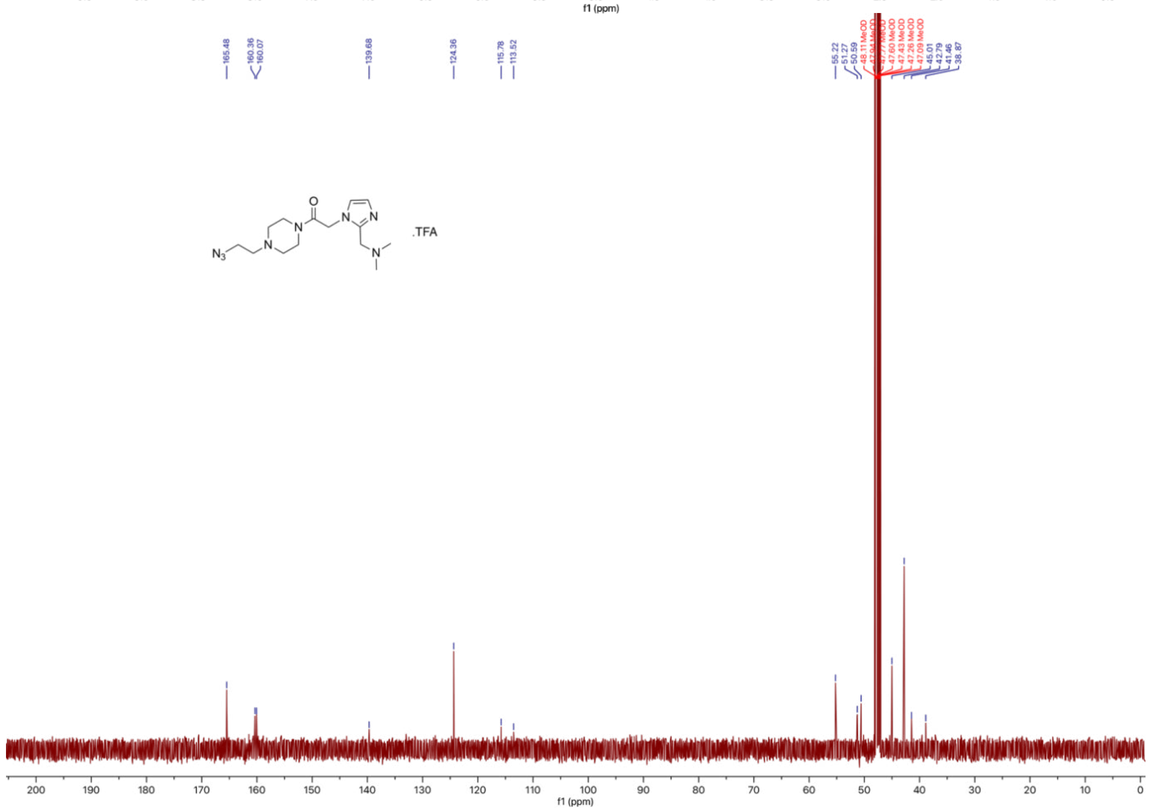

Compound 15

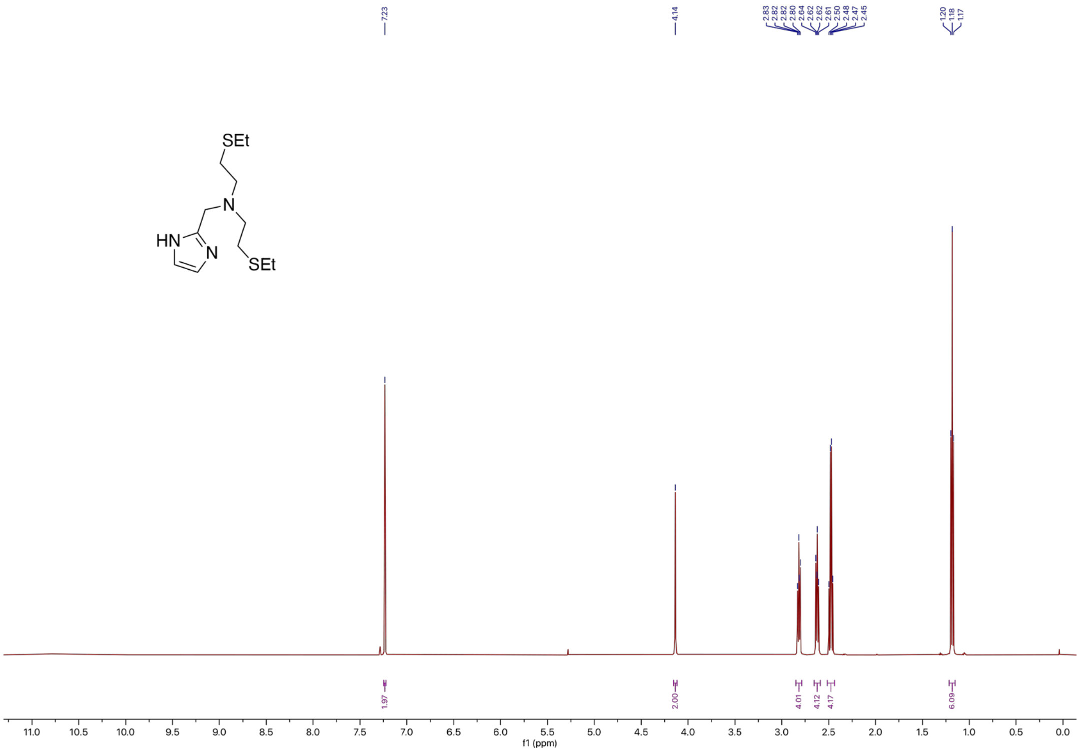

7.23
4.14
2.83
2.82
2.82
2.80
2.64
2.62
2.62
2.61
2.50
2.48
2.47
2.45
1.20
1.18
1.17
SEt
N
HN
N
SEt
1.97
2.00
4.01
4.12
4.17
6.09
f1 (ppm)

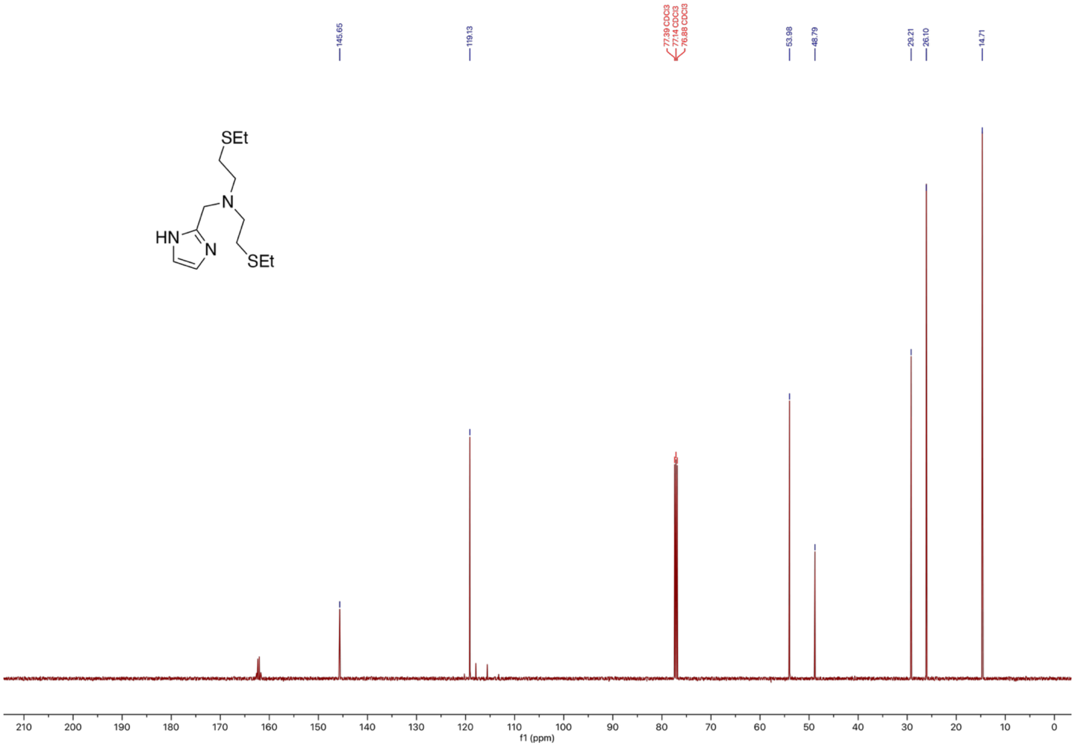

145.65
119.13
77.39 CDCl3
77.14 CDCl3
76.88 CDCl3
53.98
48.79
29.21
26.10
14.71
SEt
N
HN
N
SEt
f1 (ppm)

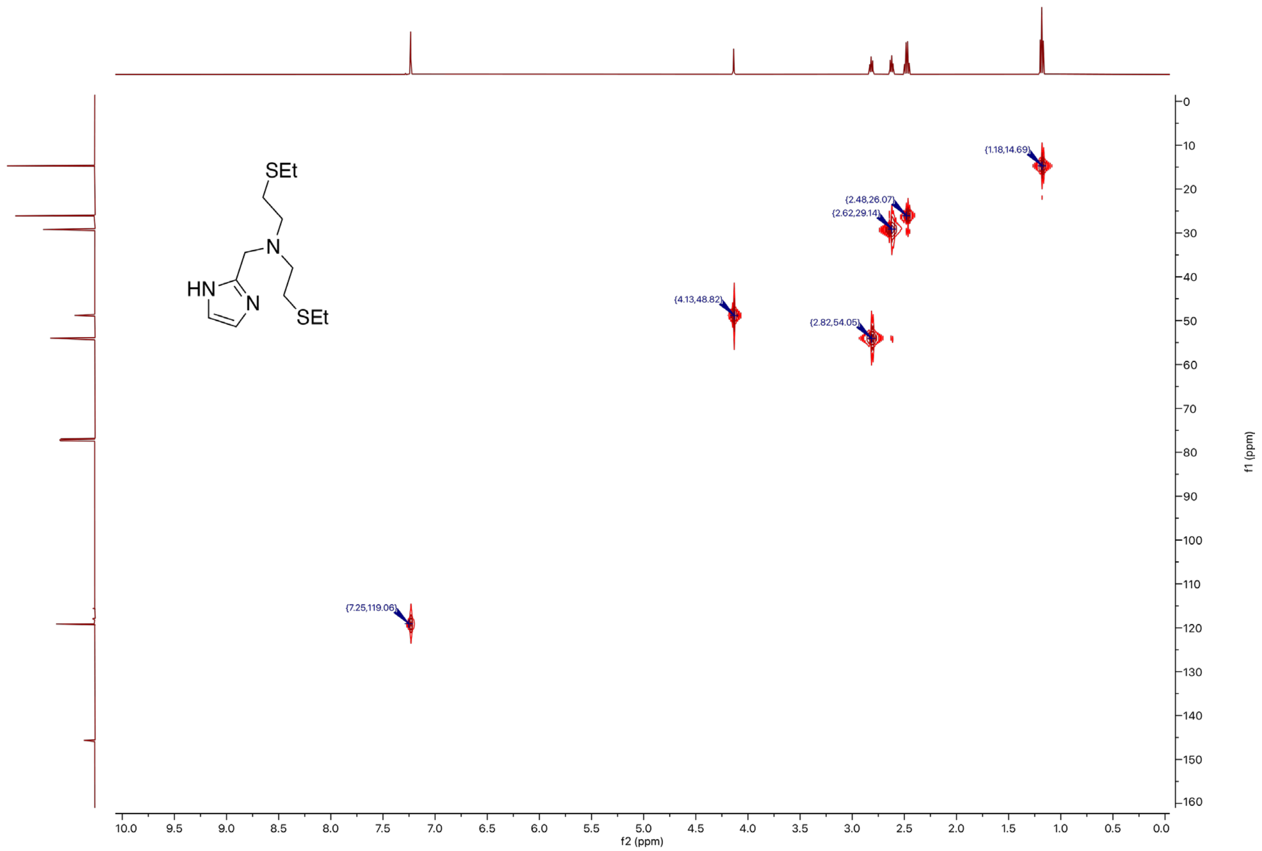
SEt
N
HN
N
SEt
{1.18,14.69}
{2.48,26.07}
{2.62,29.14}
{4.13,48.82}
{2.82,54.05}
{7.25,119.06}
f2 (ppm)
f1 (ppm)

Compound 16

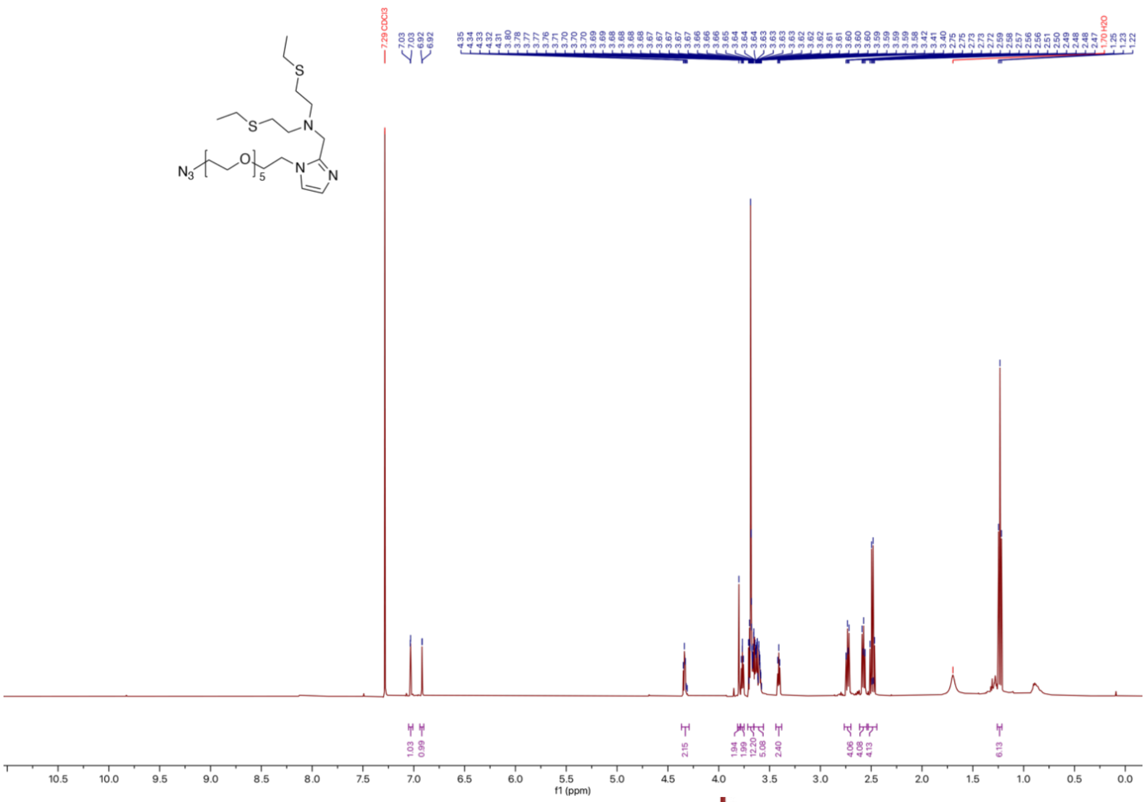

N3
O
5
S
N
N
N
7.29 CDCl3
1.70 H2O
1.03
0.99
2.15
1.94
1.99
12.20
5.08
2.40
4.06
4.08
4.13
6.13
f1 (ppm)

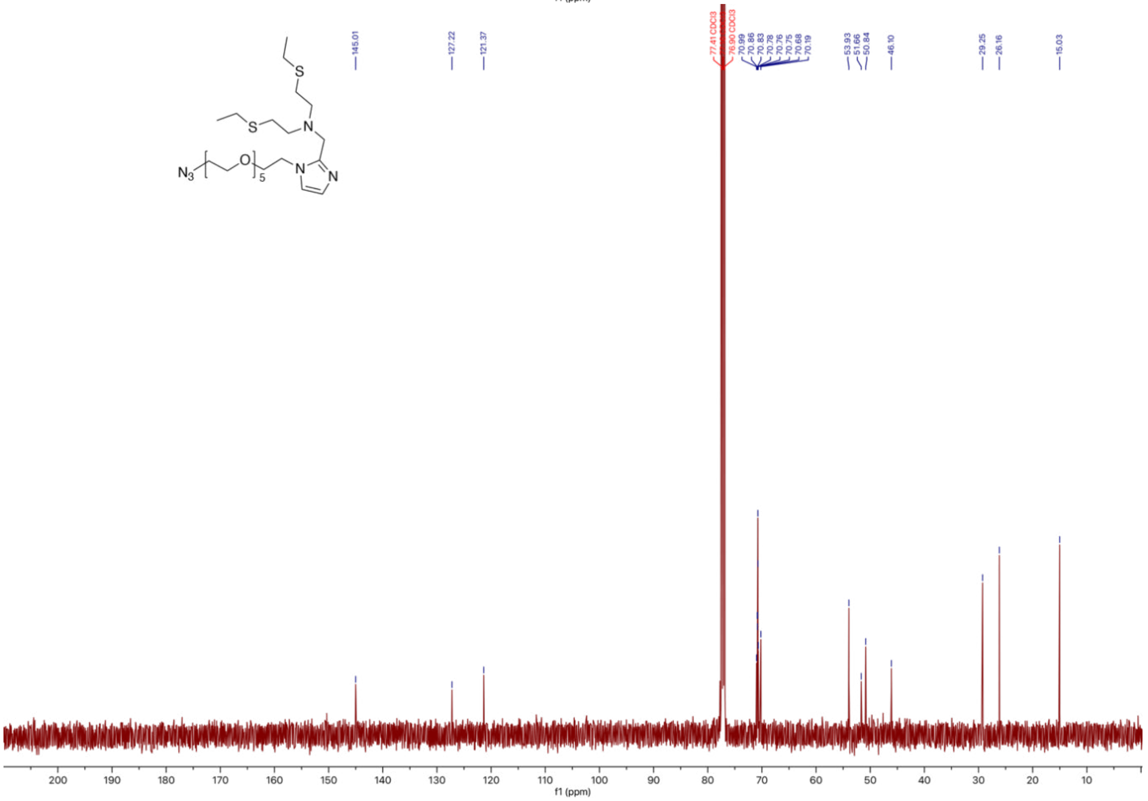

N3
O
5
S
N
N
N
145.01
127.22
121.37
77.41 CDCl3
76.90 CDCl3
70.99
70.86
70.83
70.78
70.76
70.75
70.68
70.19
53.93
51.66
50.84
46.10
29.25
26.16
15.03
f1 (ppm)

Compound 17

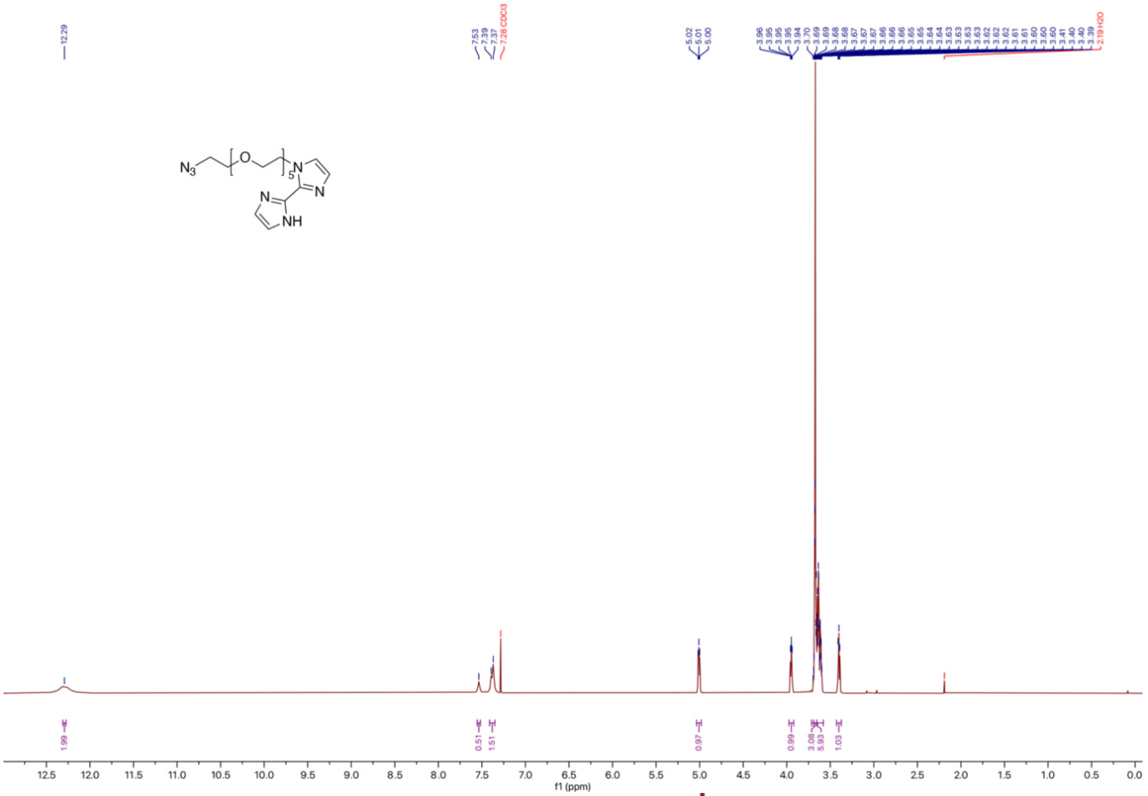

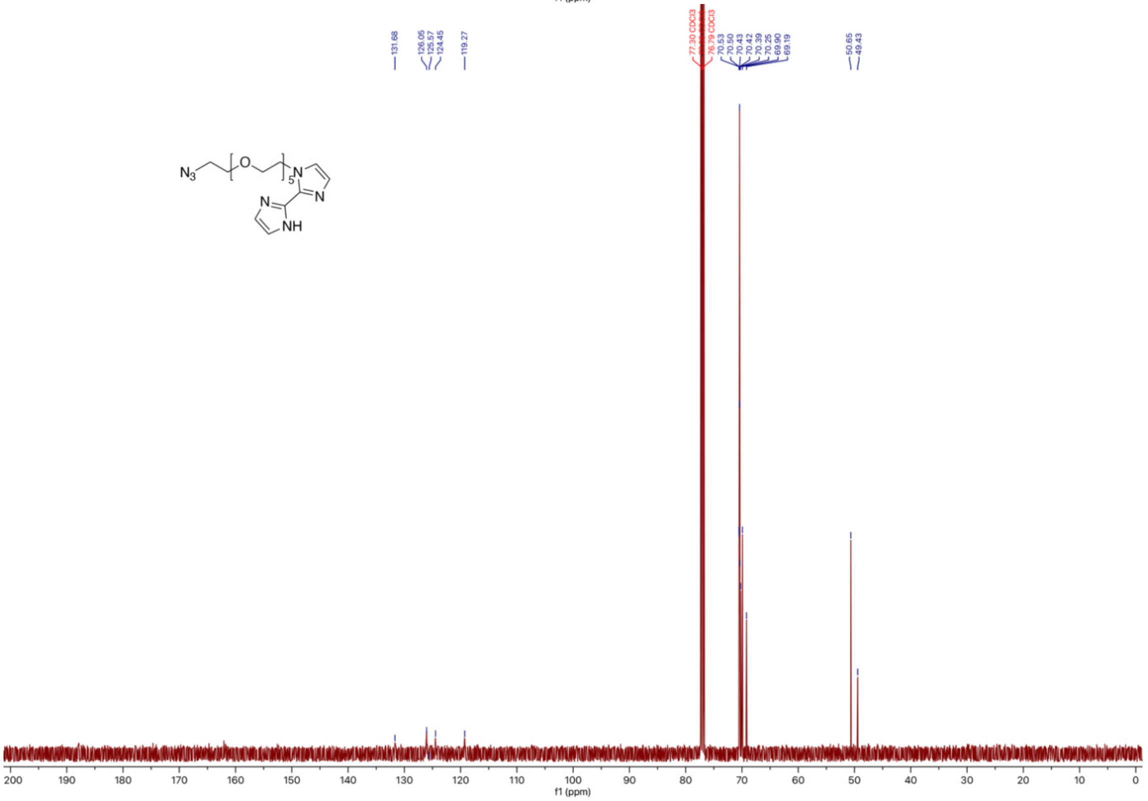

Compound 18

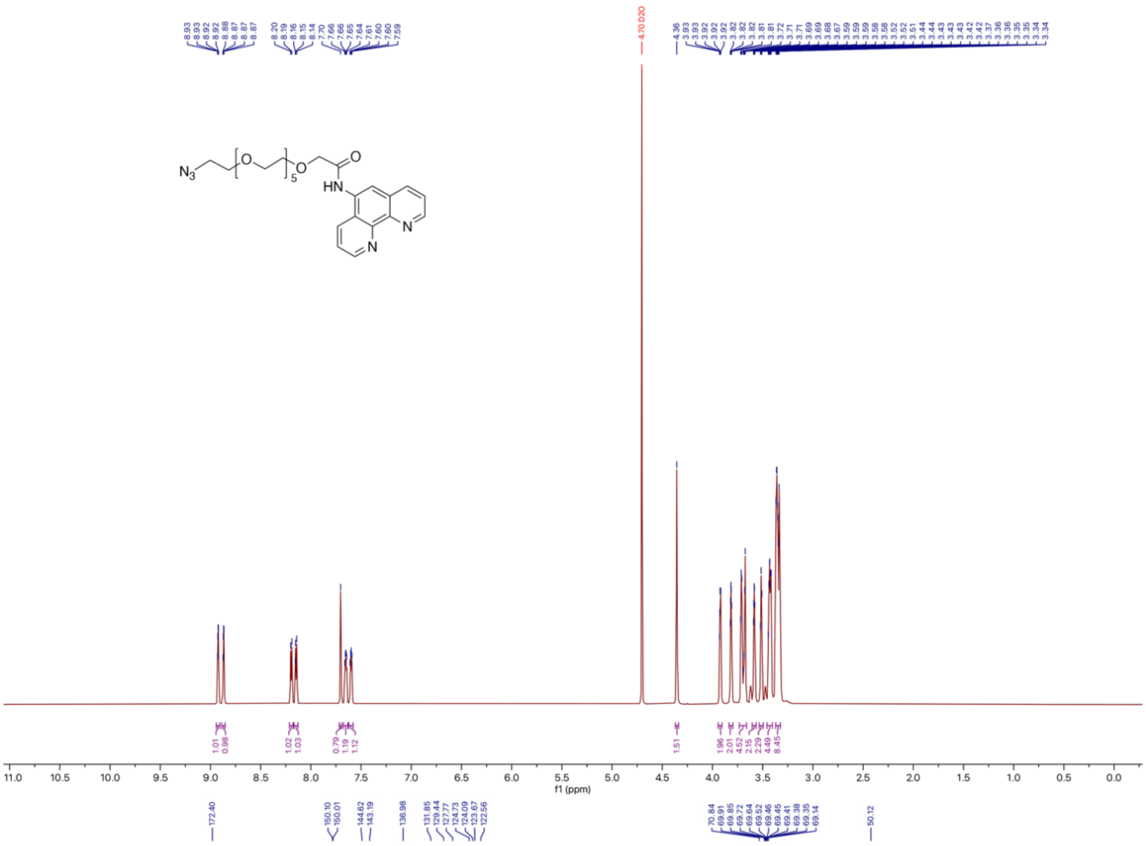

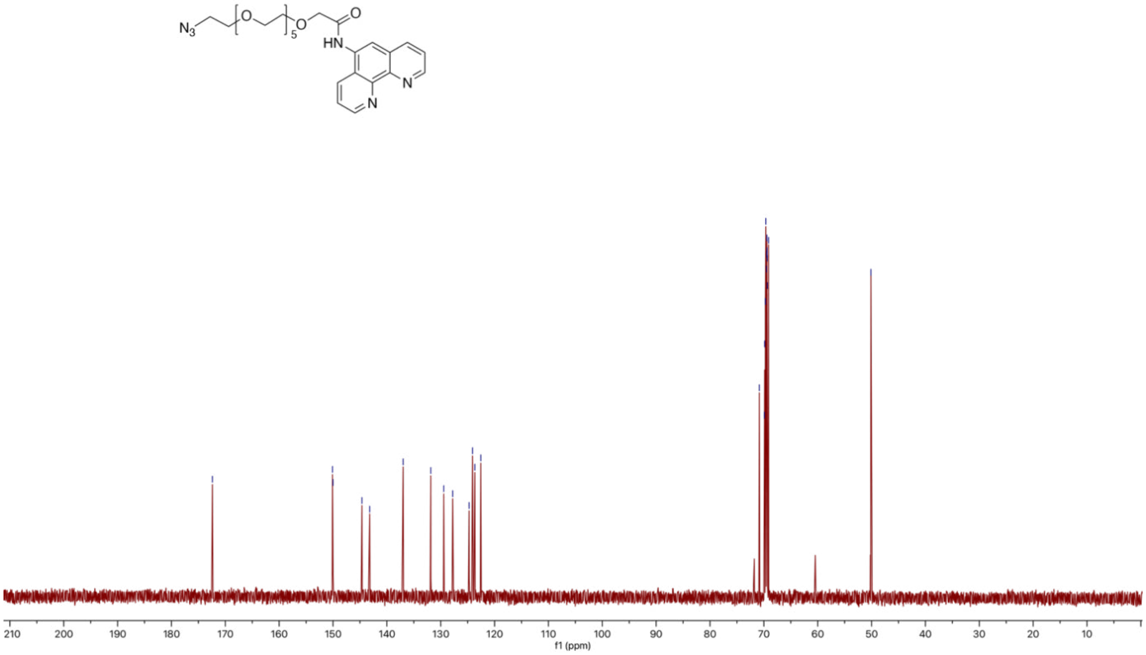

Compound 19

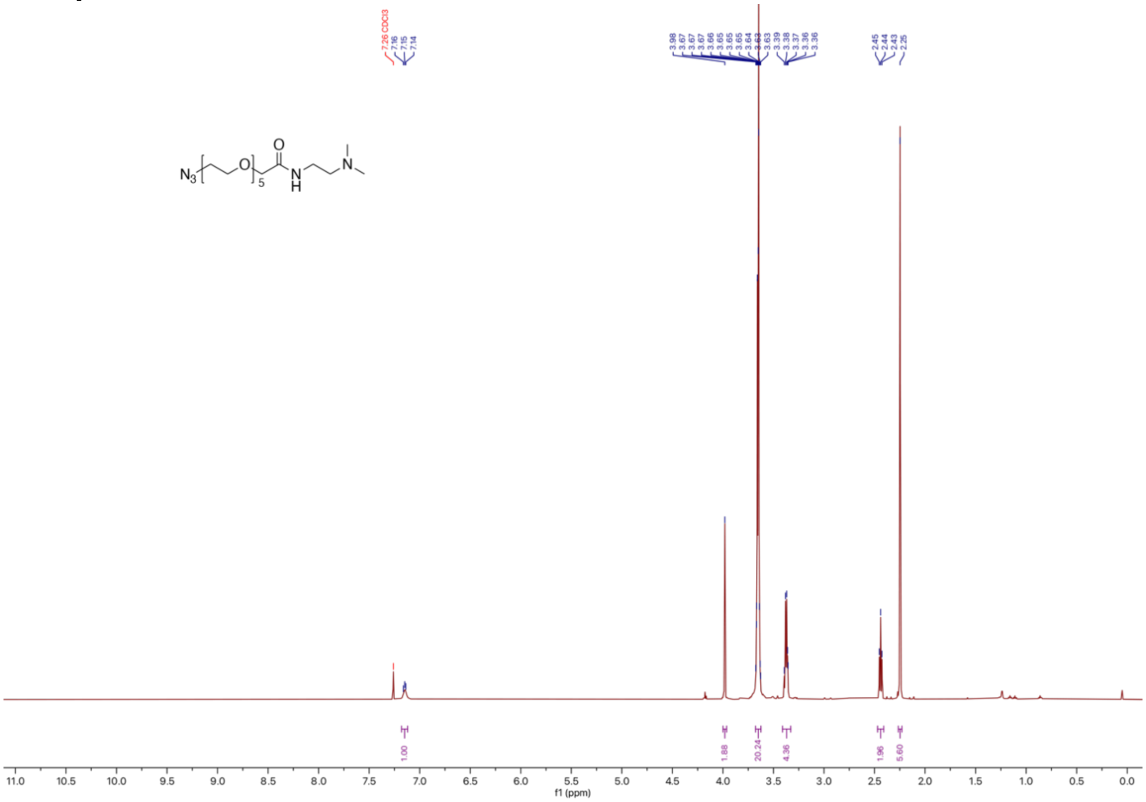

7.26 CDCl3
7.16
7.15
7.14
3.98
3.67
3.67
3.67
3.66
3.65
3.65
3.65
3.64
3.63
3.39
3.38
3.37
3.36
3.36
2.45
2.44
2.43
2.25
1.00
1.88
20.24
4.36
1.96
5.60
f1 (ppm)

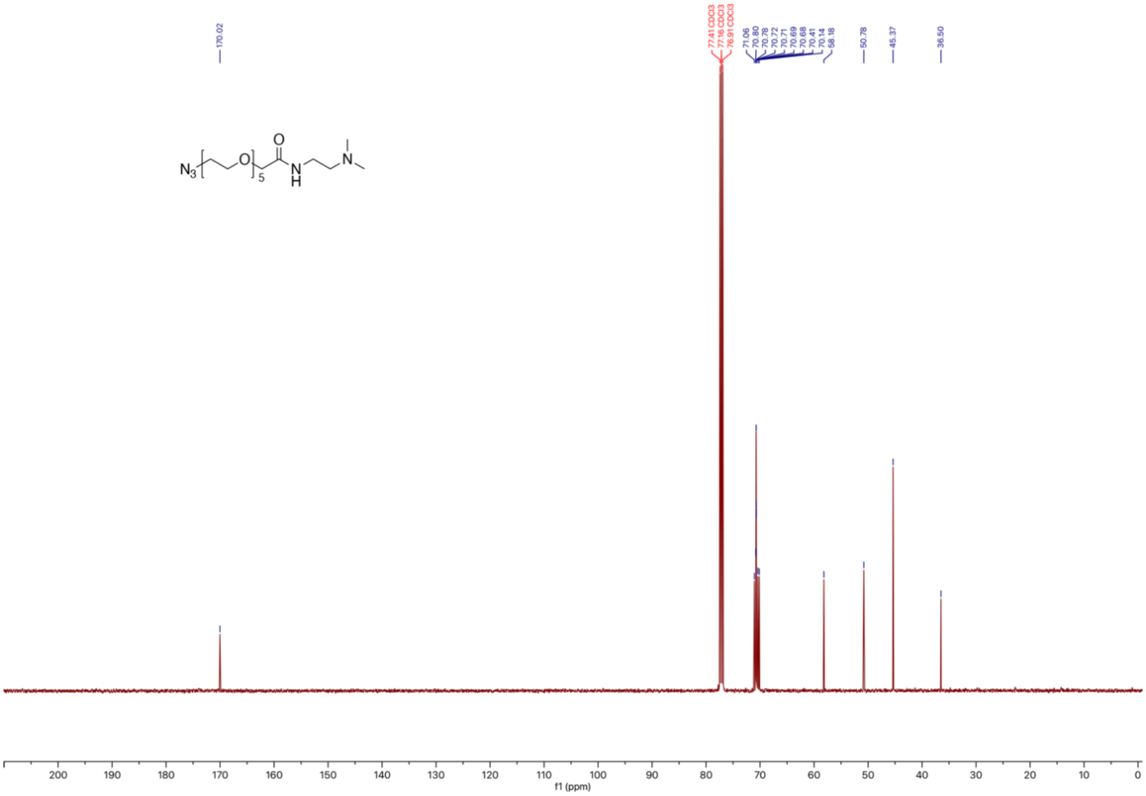

170.02
77.41 CDCl3
77.16 CDCl3
76.91 CDCl3
71.06
70.80
70.78
70.72
70.71
70.69
70.68
70.41
70.14
58.18
50.78
45.37
36.50
f1 (ppm)

Compound 20

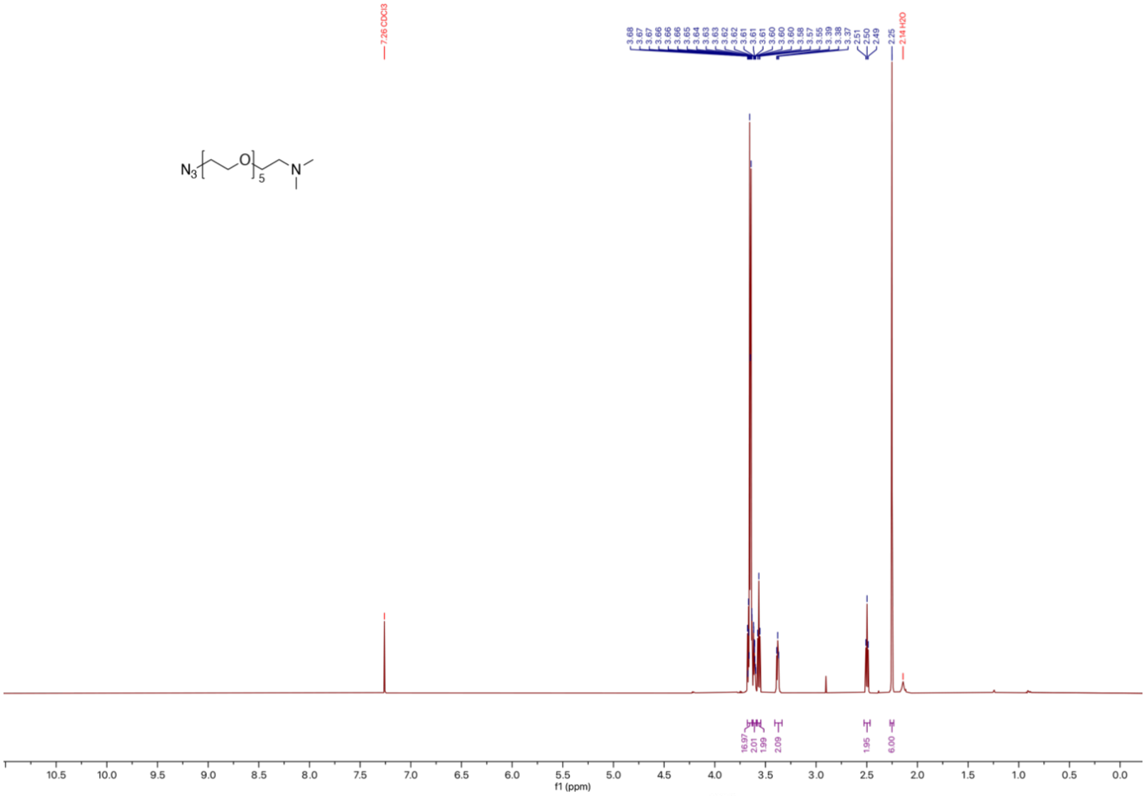

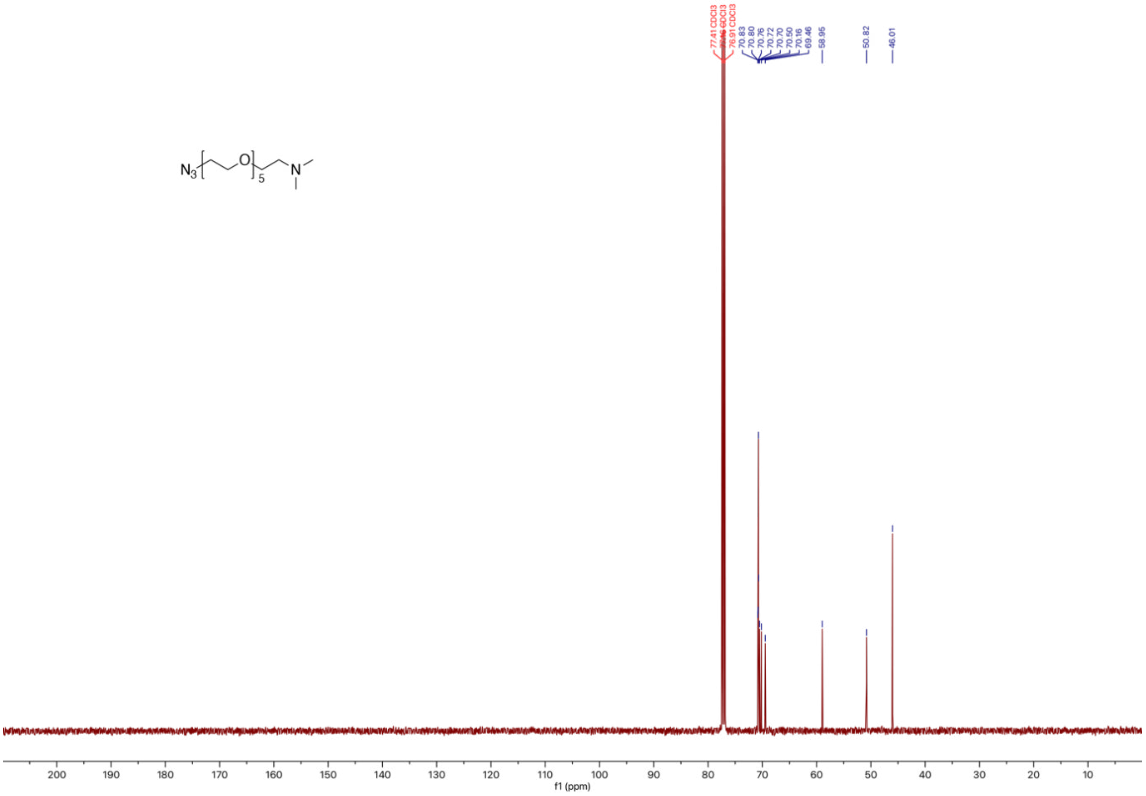

Compound 21

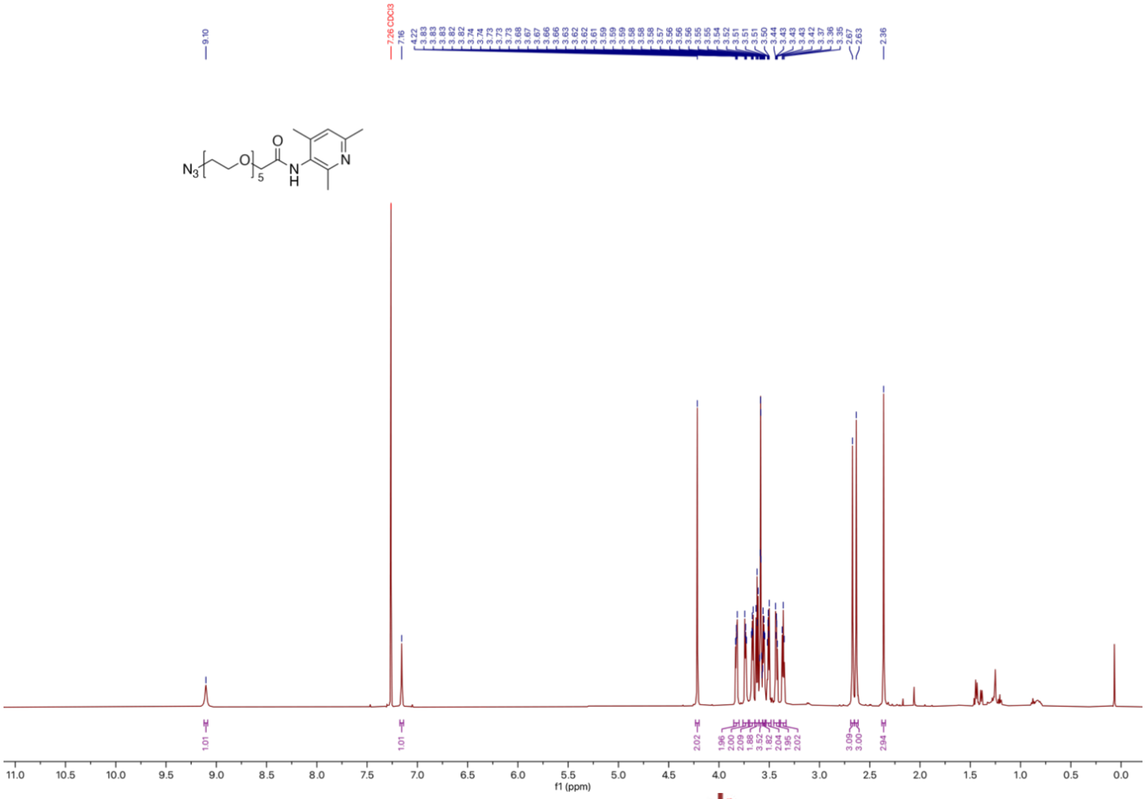

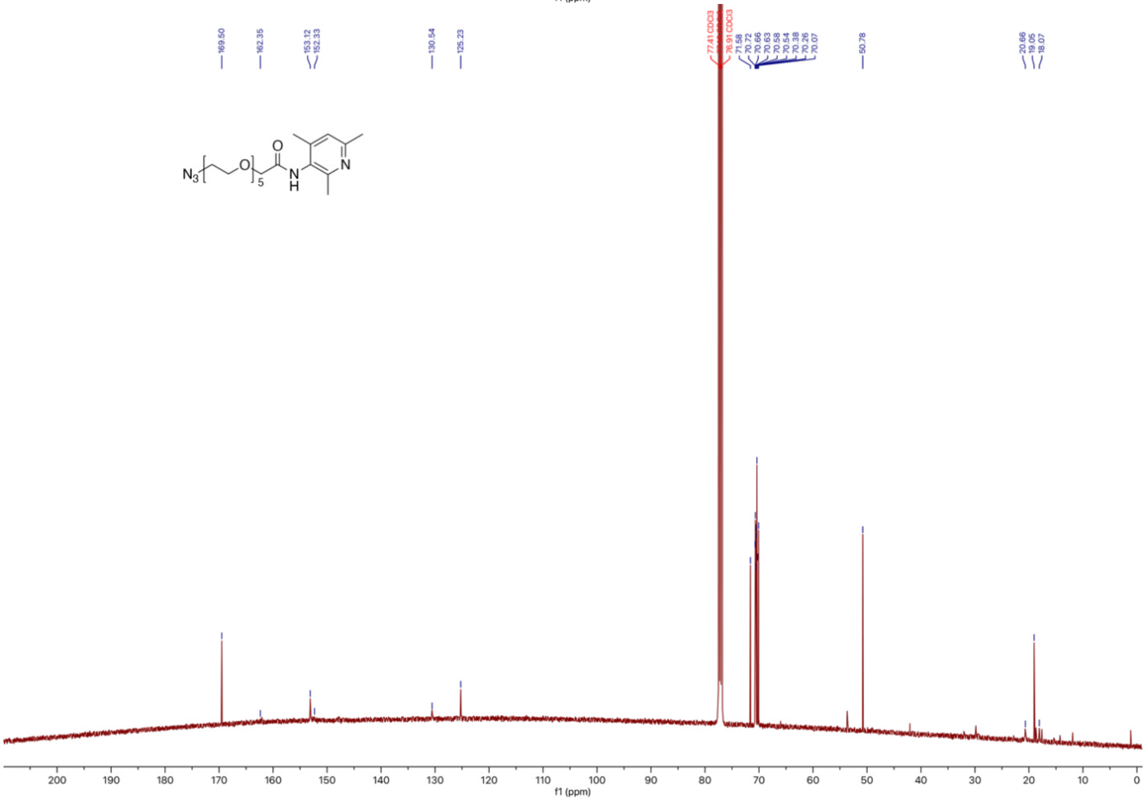

Compound 22

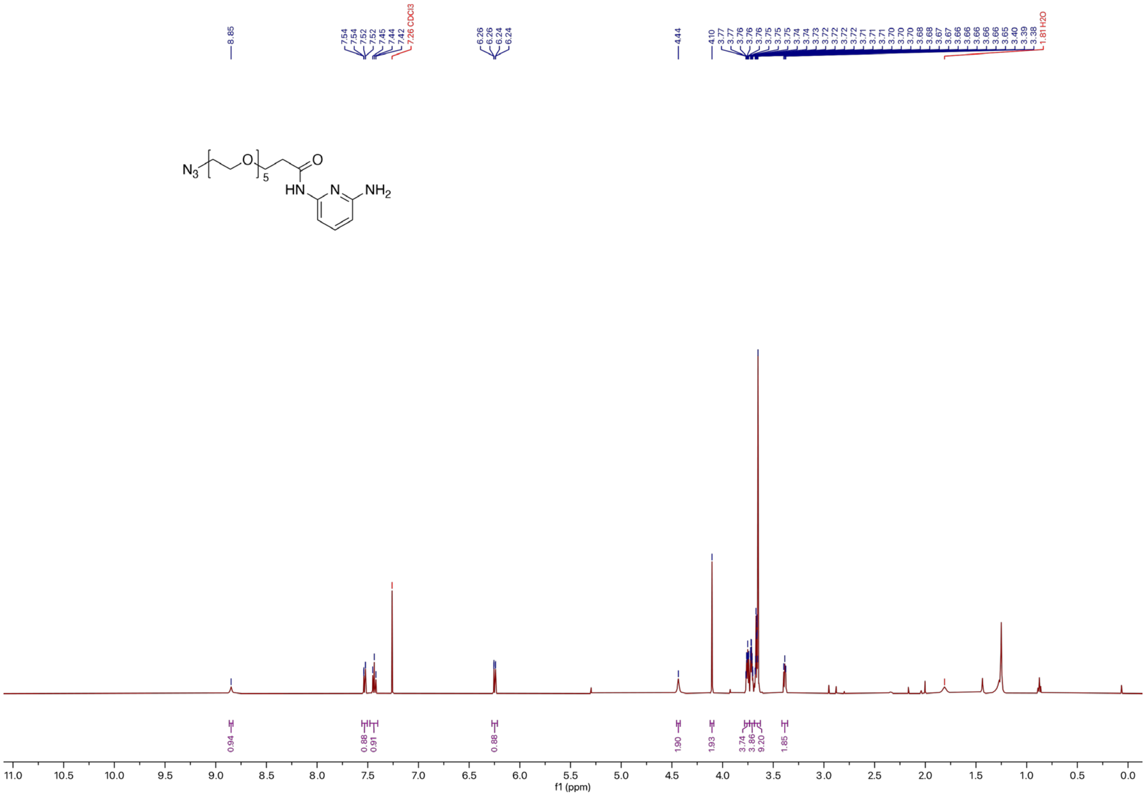

8.85
7.54
7.54
7.52
7.52
7.45
7.44
7.42
7.26 CDCl3
6.26
6.26
6.24
6.24
4.44
4.10
1.81 H2O
0.94
0.88
0.91
0.88
1.90
1.93
3.74
3.86
9.20
1.85
f1 (ppm)

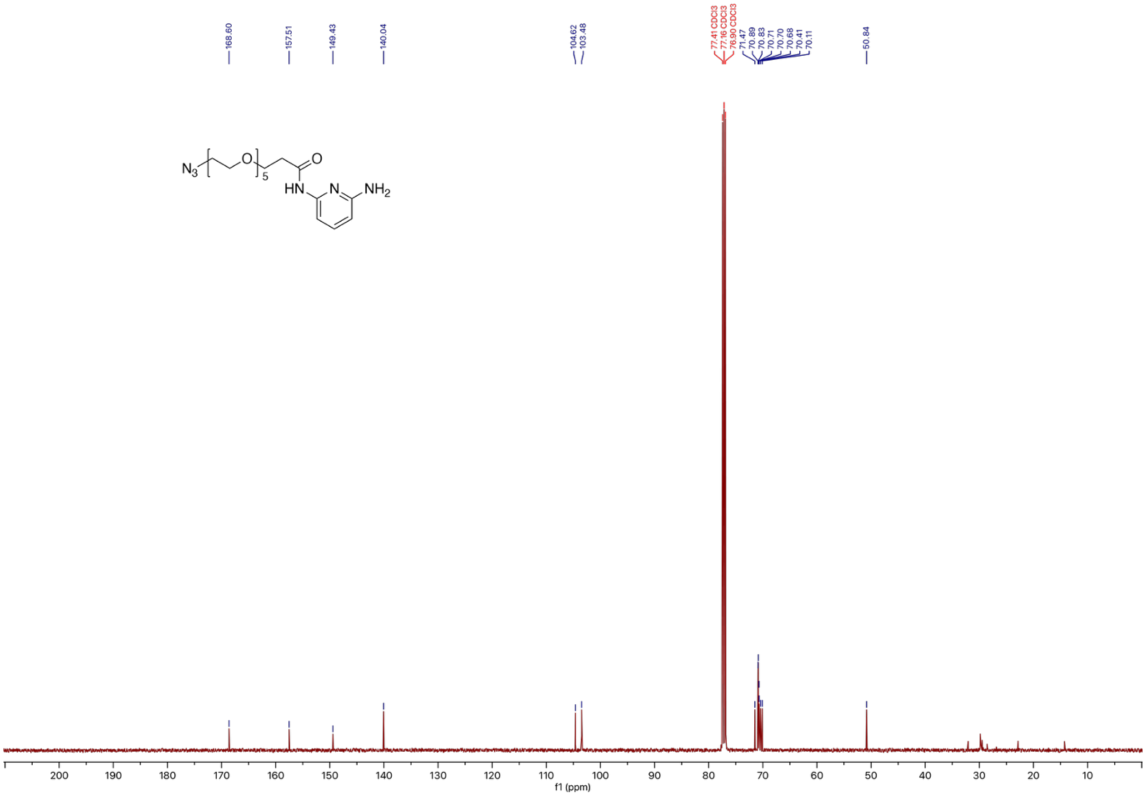

168.60
157.51
149.43
140.04
104.62
103.48
77.41 CDCl3
77.16 CDCl3
76.90 CDCl3
71.47
70.89
70.83
70.71
70.70
70.68
70.41
70.11
50.84
f1 (ppm)

Compound 23

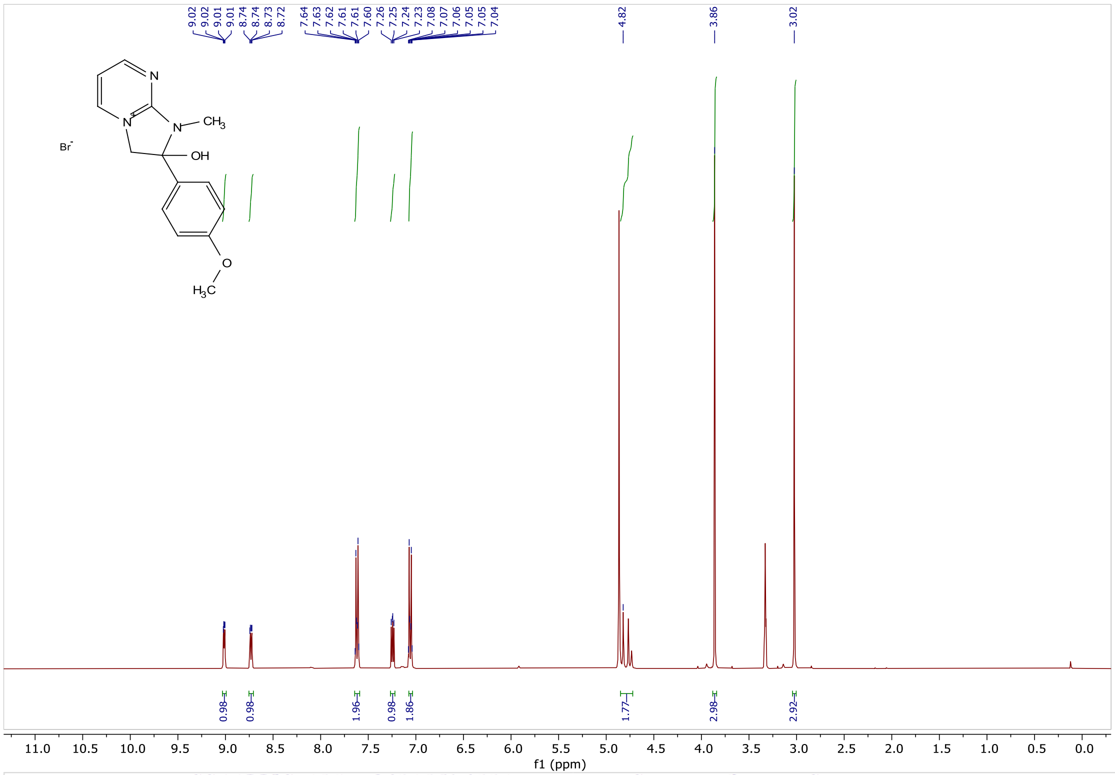

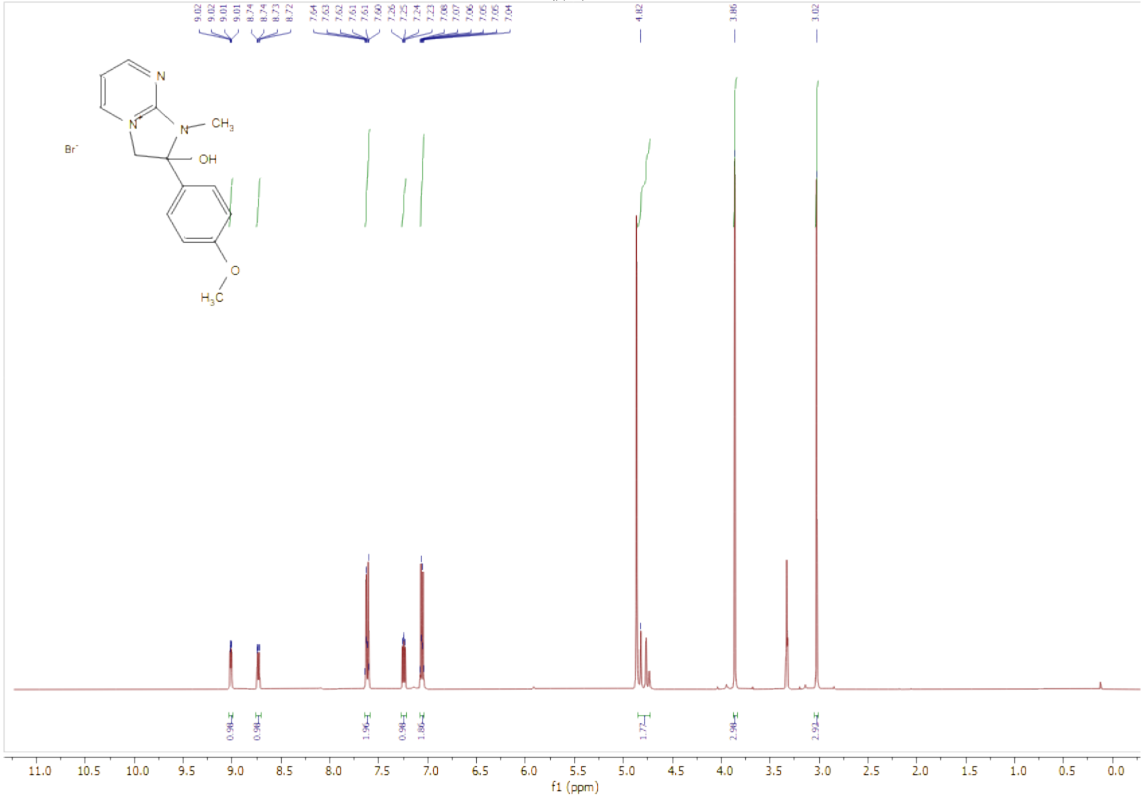

Compound 24

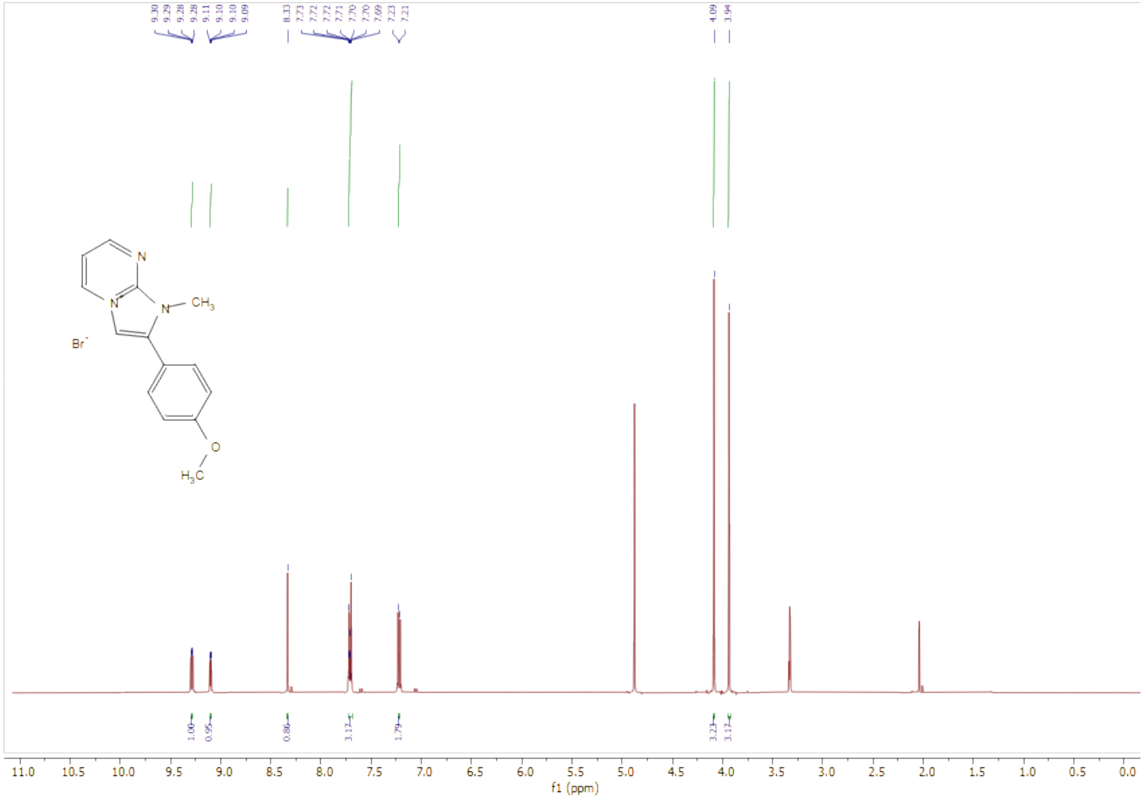

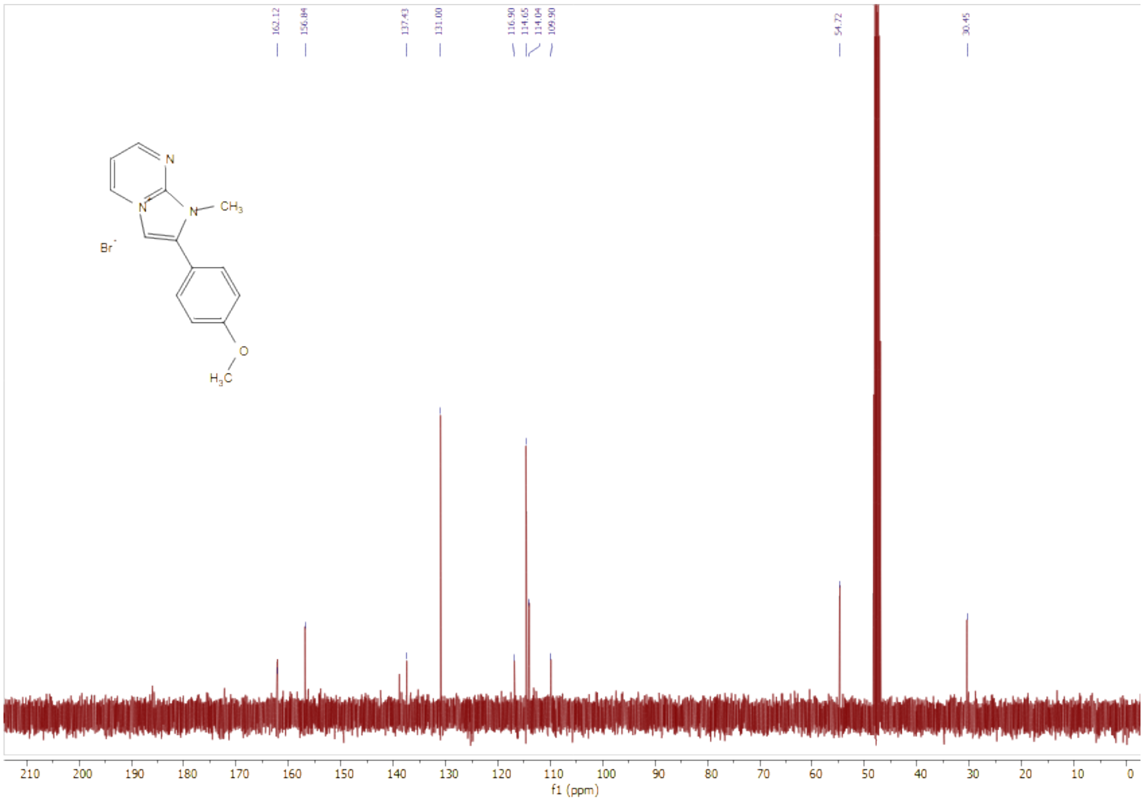

Compound 25

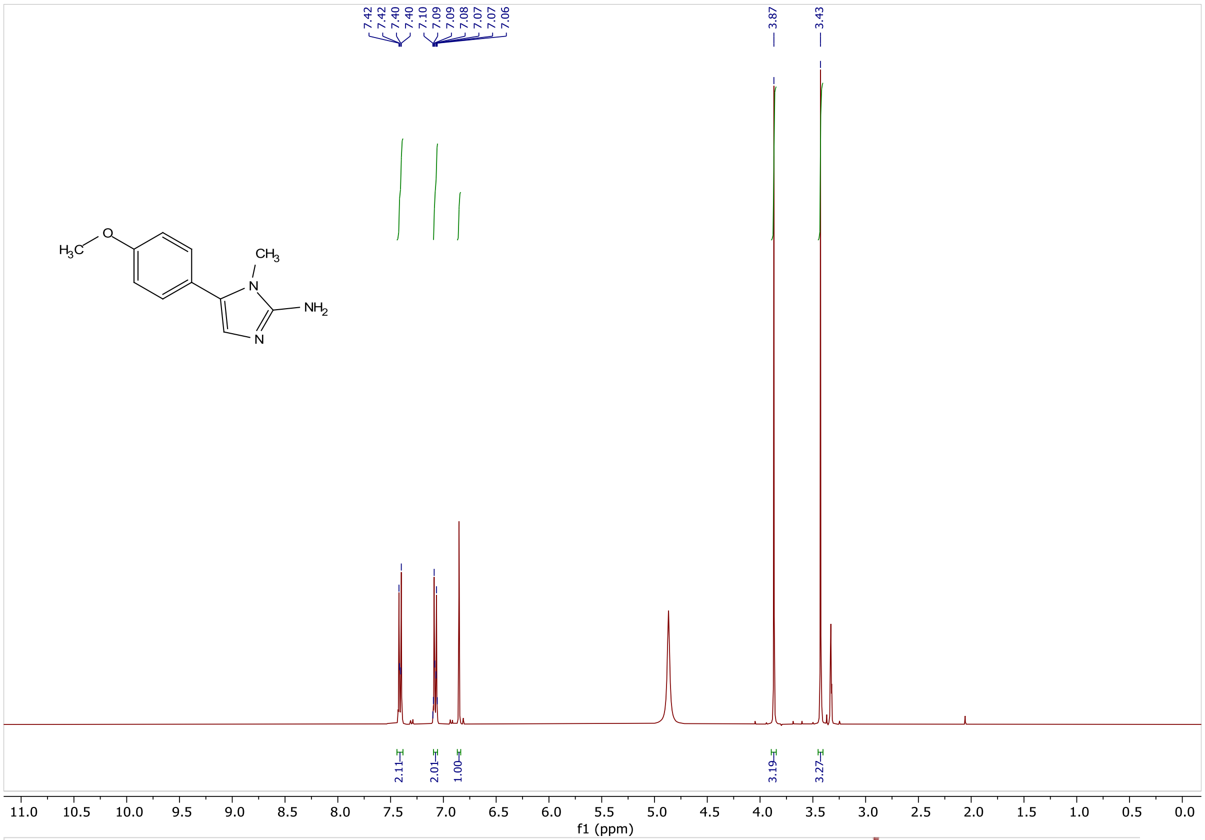
7.42
7.42
7.40
7.40
7.10
7.09
7.09
7.08
7.07
7.07
7.06
3.87
3.43
H3C
O
CH3
N
NH2
N
2.11
2.01
1.00
3.19
3.27
11.0
10.5
10.0
9.5
9.0
8.5
8.0
7.5
7.0
6.5
6.0
5.5
5.0
4.5
4.0
3.5
3.0
2.5
2.0
1.5
1.0
0.5
0.0
f1 (ppm)

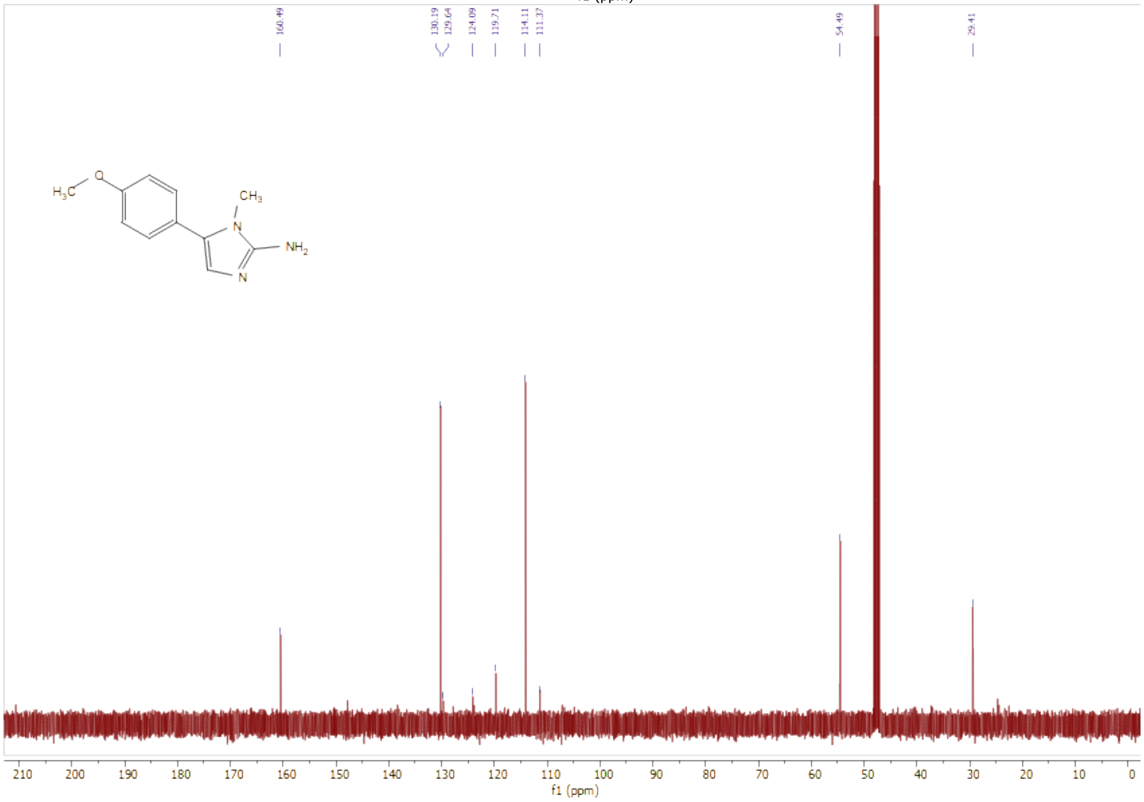
160.49
130.19
129.64
124.09
119.71
114.11
111.37
54.49
29.41
H3C
O
CH3
N
NH2
N
210
200
190
180
170
160
150
140
130
120
110
100
90
80
70
60
50
40
30
20
10
0
f1 (ppm)

Binder–1

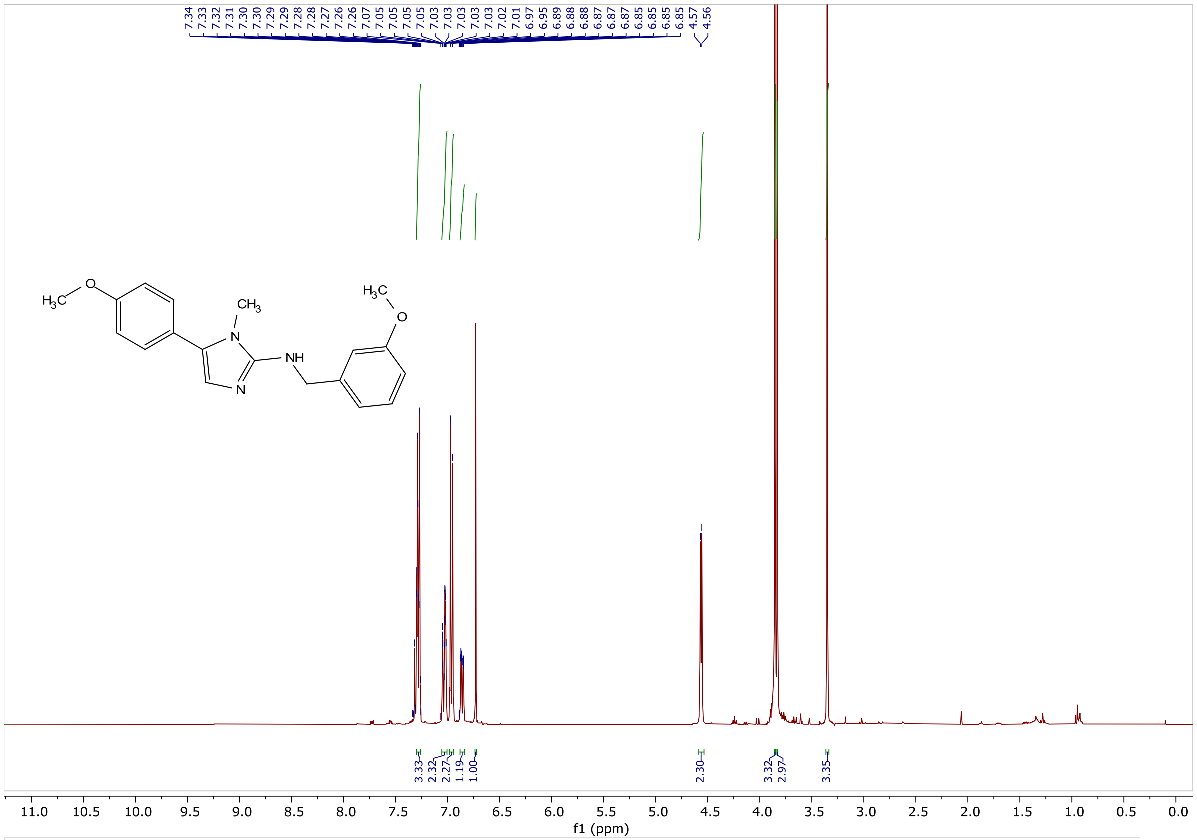
7.34 7.33 7.32 7.31 7.30 7.30 7.29 7.29 7.28 7.28 7.27 7.26 7.26 7.07 7.05 7.05 7.05 7.05 7.03 7.03 7.03 7.03 7.03 7.02 7.01 6.97 6.95 6.89 6.89 6.88 6.88 6.87 6.87 6.87 6.85 6.85 6.85 6.85 4.57 4.56
H3C O CH3 N NH N H3C O
3.33 2.32 2.27 1.19 1.00 2.30 3.32 2.97 3.35
11.0 10.5 10.0 9.5 9.0 8.5 8.0 7.5 7.0 6.5 6.0 5.5 5.0 4.5 4.0 3.5 3.0 2.5 2.0 1.5 1.0 0.5 0.0
f1 (ppm)

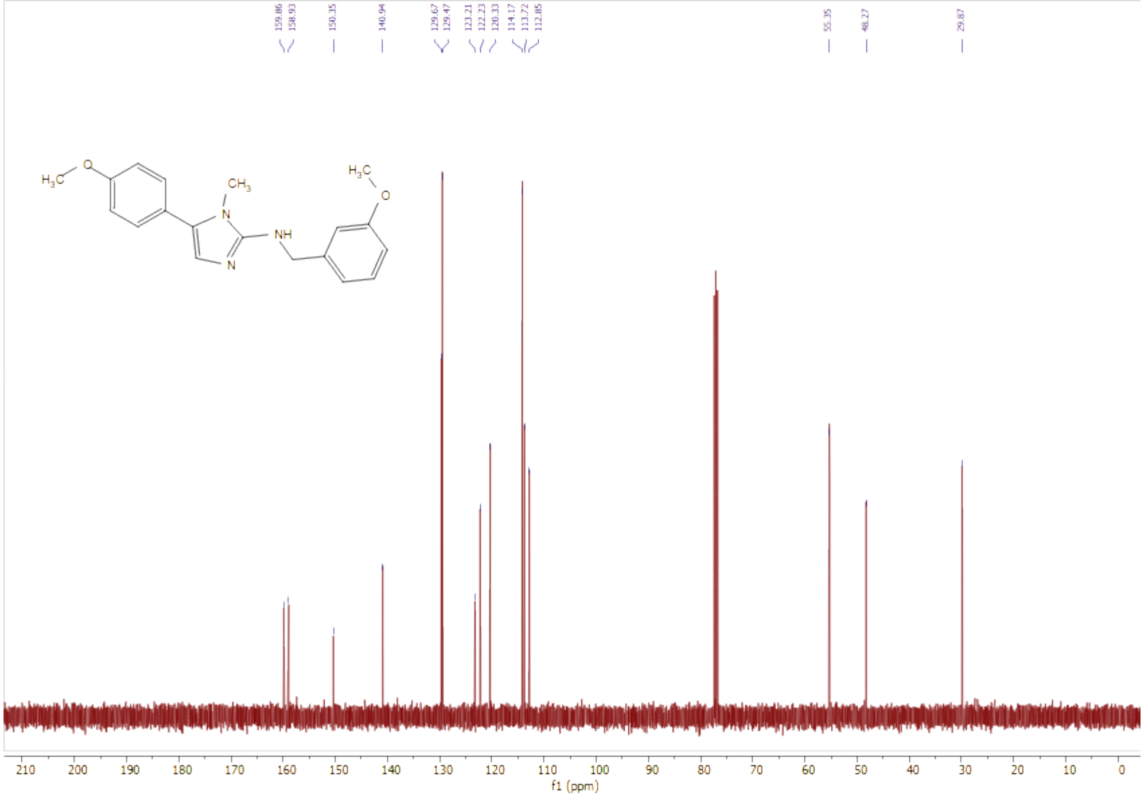
159.86 158.93 150.35 140.94 129.67 129.47 123.21 122.23 120.33 114.17 113.72 112.85 55.35 48.27 29.87
H3C O CH3 N NH N H3C O
210 200 190 180 170 160 150 140 130 120 110 100 90 80 70 60 50 40 30 20 10 0
f1 (ppm)

Compound 26

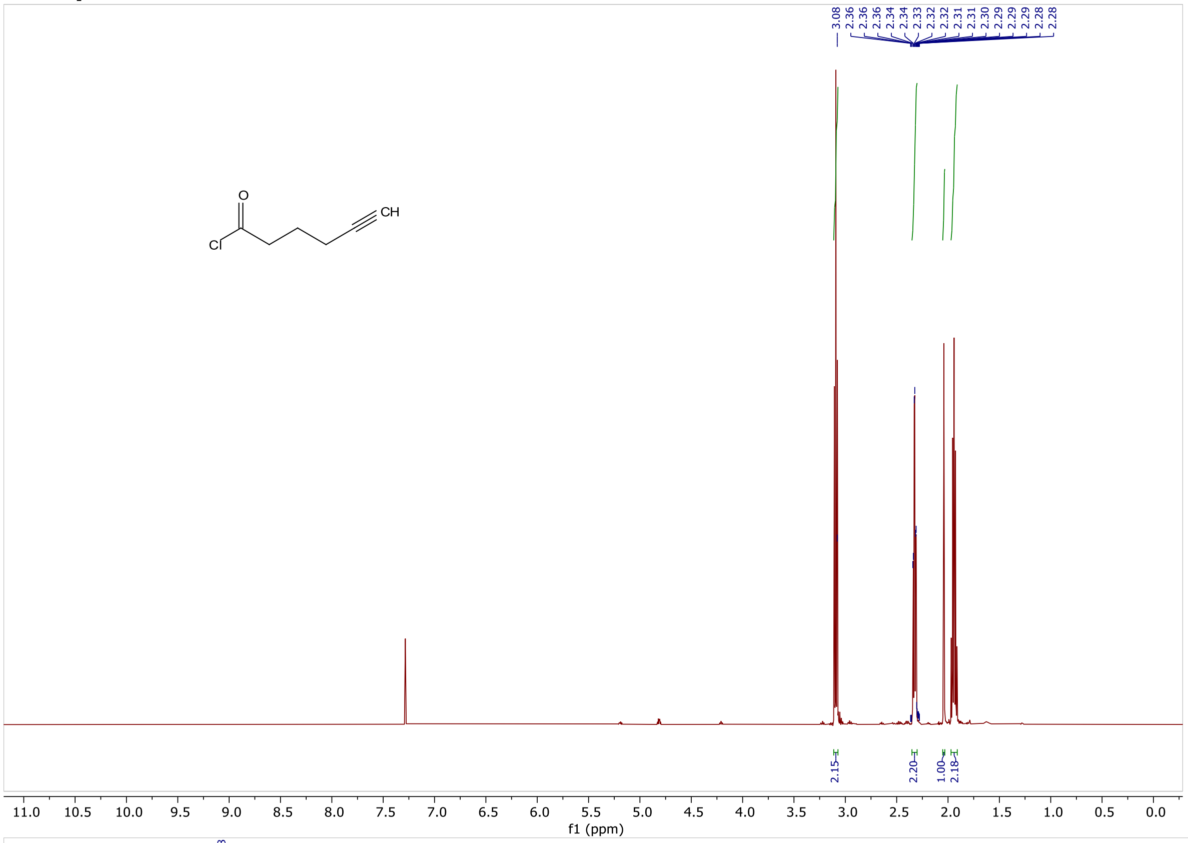

3.08
2.36
2.36
2.36
2.34
2.34
2.33
2.32
2.32
2.31
2.31
2.30
2.29
2.29
2.29
2.28
2.28
O
Cl
CH
2.15
2.20
1.00
2.18
11.0 10.5 10.0 9.5 9.0 8.5 8.0 7.5 7.0 6.5 6.0 5.5 5.0 4.5 4.0 3.5 3.0 2.5 2.0 1.5 1.0 0.5 0.0
f1 (ppm)

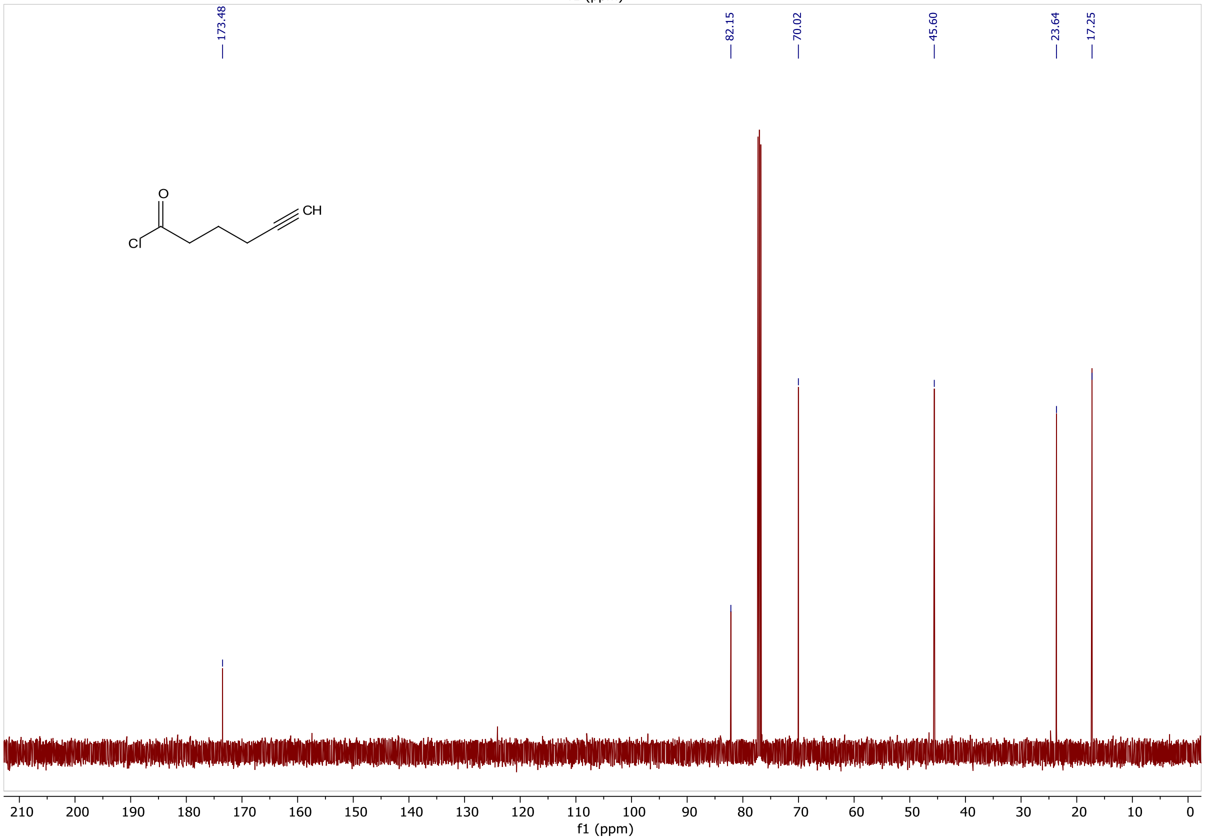

173.48
82.15
70.02
45.60
23.64
17.25
O
Cl
CH
210 200 190 180 170 160 150 140 130 120 110 100 90 80 70 60 50 40 30 20 10 0
f1 (ppm)

PINAD-1

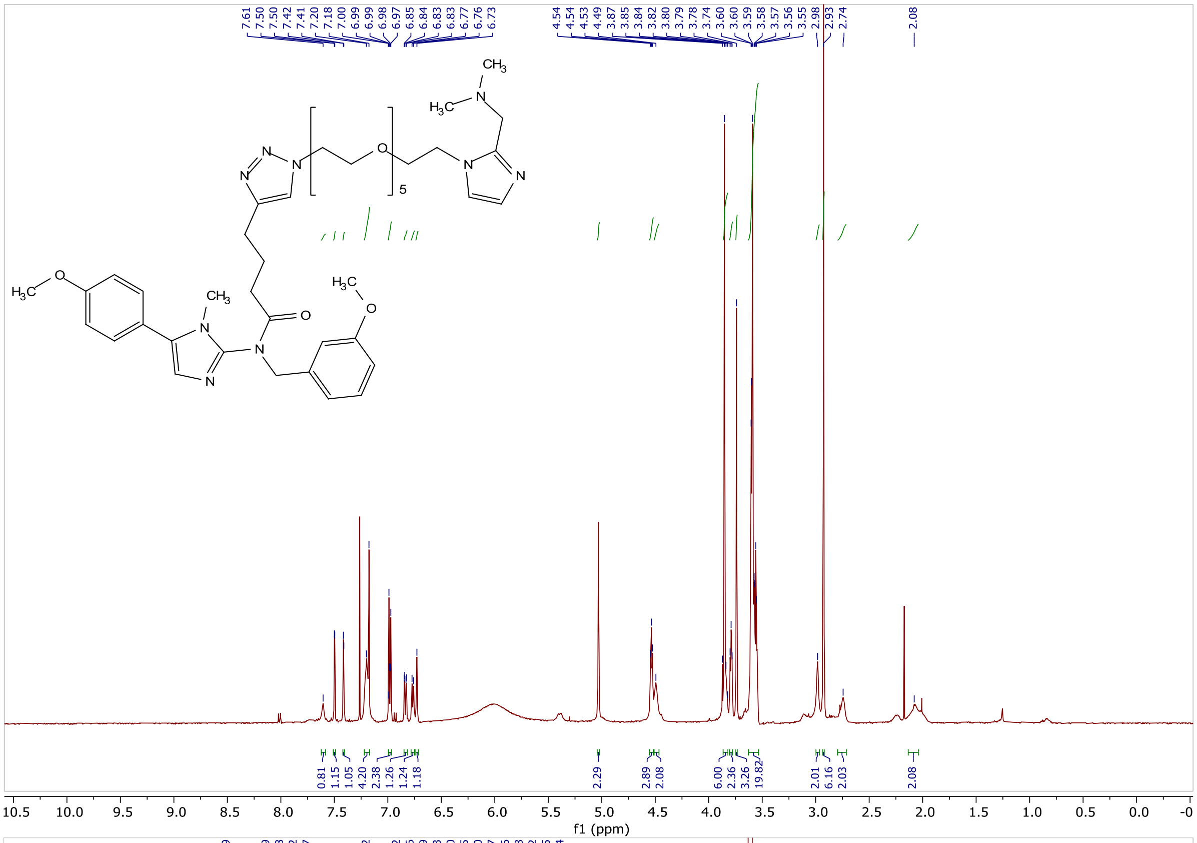
7.61
7.50
7.50
7.42
7.41
7.20
7.18
7.00
6.99
6.99
6.98
6.97
6.85
6.84
6.83
6.83
6.77
6.76
6.73
4.54
4.54
4.53
4.49
3.87
3.85
3.84
3.82
3.80
3.79
3.78
3.74
3.60
3.60
3.59
3.58
3.57
3.56
3.55
2.98
2.93
2.74
2.08
CH3
H3C
N
N
N
N
O
5
N
N
H3C
O
CH3
N
O
N
N
H3C
O
0.81
1.15
1.05
4.20
2.38
1.26
1.24
1.18
2.29
2.89
2.08
6.00
2.36
3.26
19.82
2.01
6.16
2.03
2.08
f1 (ppm)

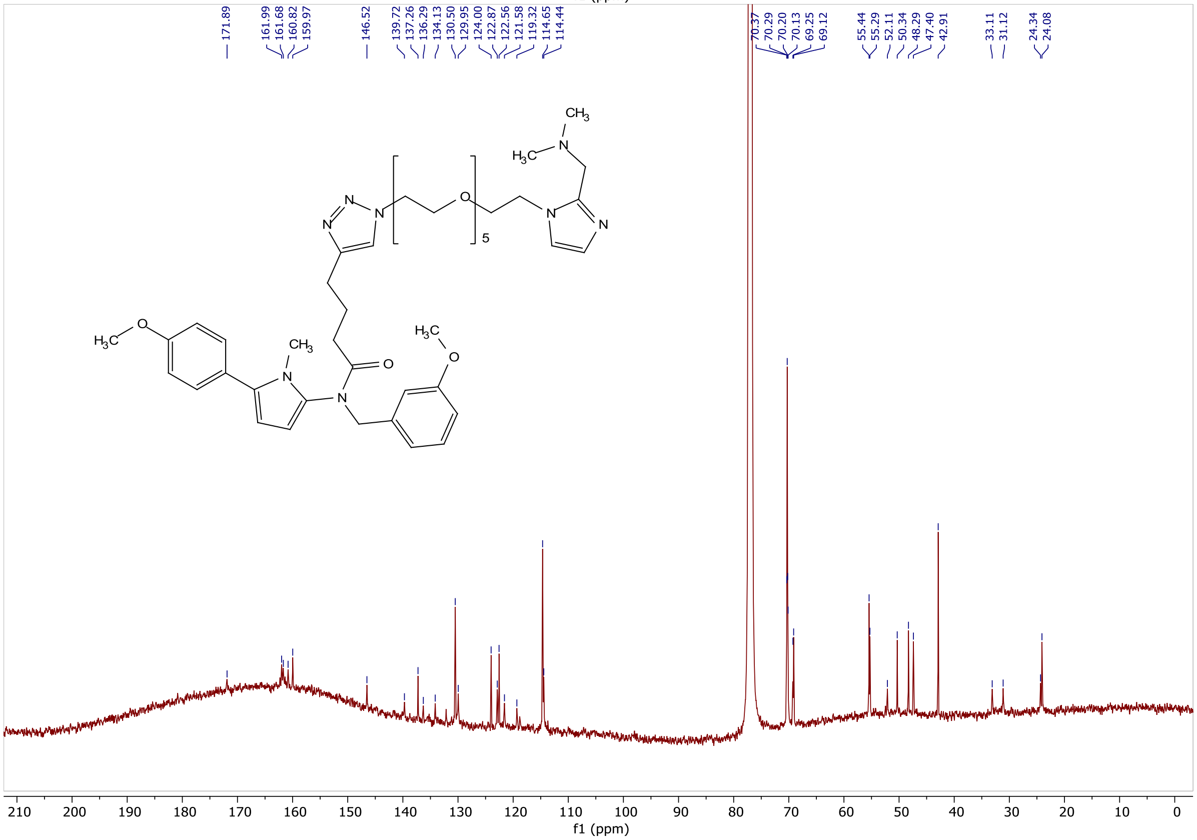
171.89
161.99
161.68
160.82
159.97
146.52
139.72
137.26
136.29
134.13
130.50
129.95
124.00
122.87
122.56
121.58
119.32
114.65
114.44
70.37
70.29
70.20
70.13
69.25
69.12
55.44
55.29
52.11
50.34
48.29
47.40
42.91
33.11
31.12
24.34
24.08
CH3
H3C
N
N
N
N
O
5
N
N
H3C
O
CH3
N
O
N
H3C
O
f1 (ppm)

Binder-2

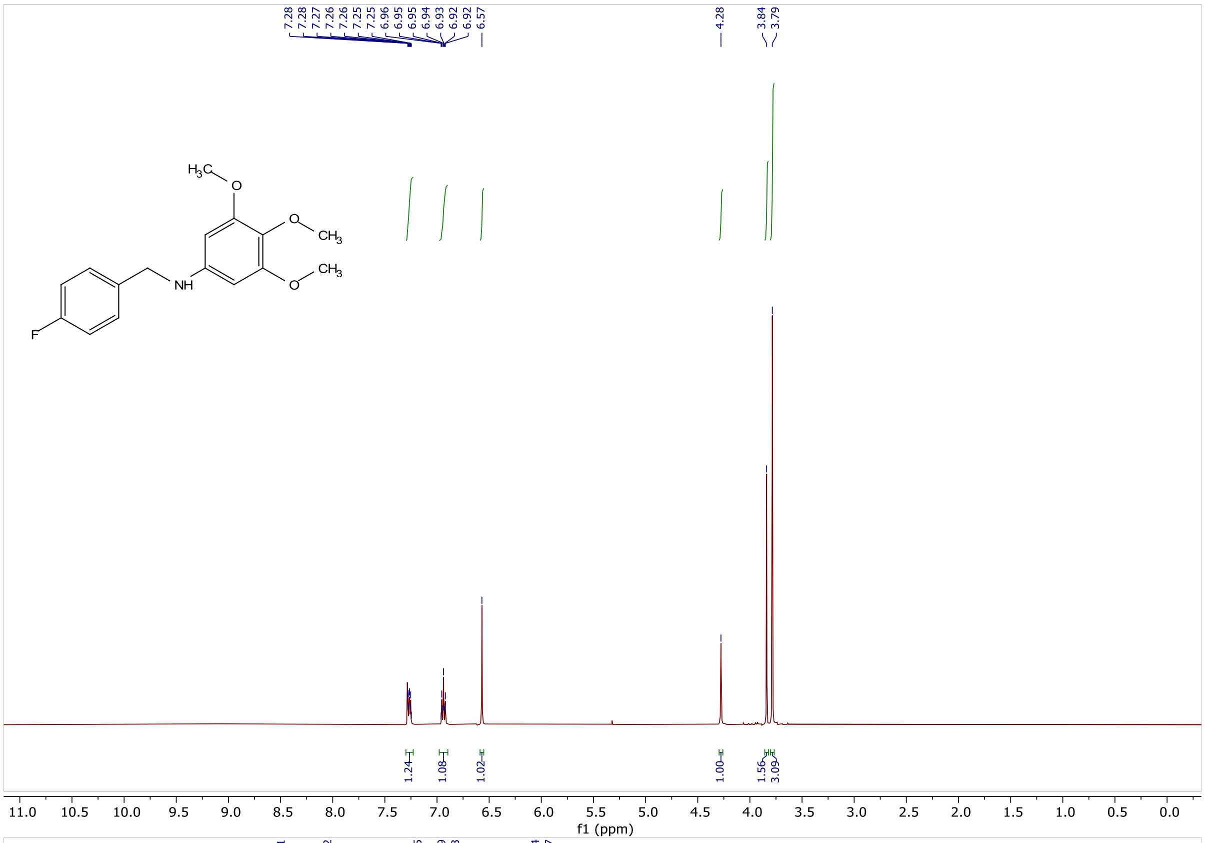
7.28
7.28
7.27
7.26
7.26
7.25
7.25
6.96
6.95
6.95
6.94
6.93
6.92
6.92
6.57
4.28
3.84
3.79
1.24
1.08
1.02
1.00
1.56
3.09
f1 (ppm)

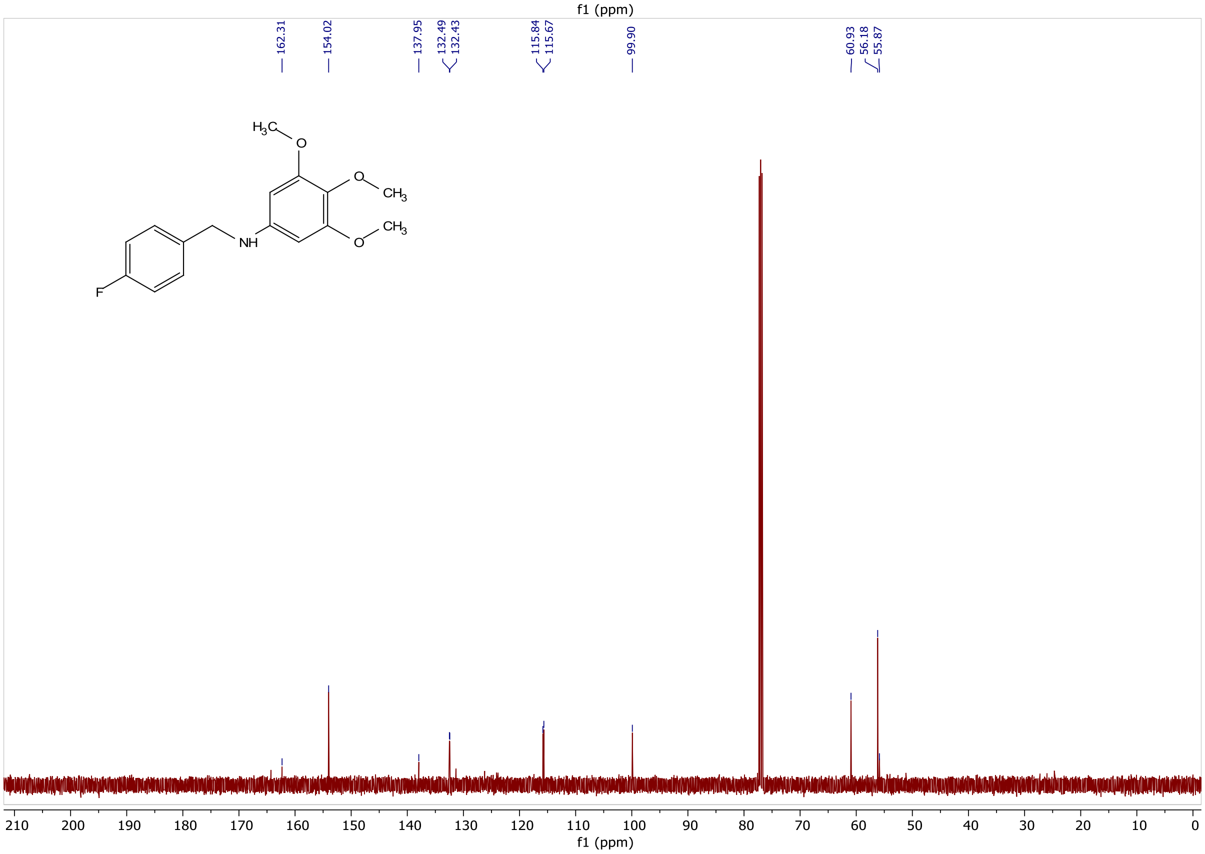
162.31
154.02
137.95
132.49
132.43
115.84
115.67
99.90
60.93
56.18
55.87
f1 (ppm)

Compound 27

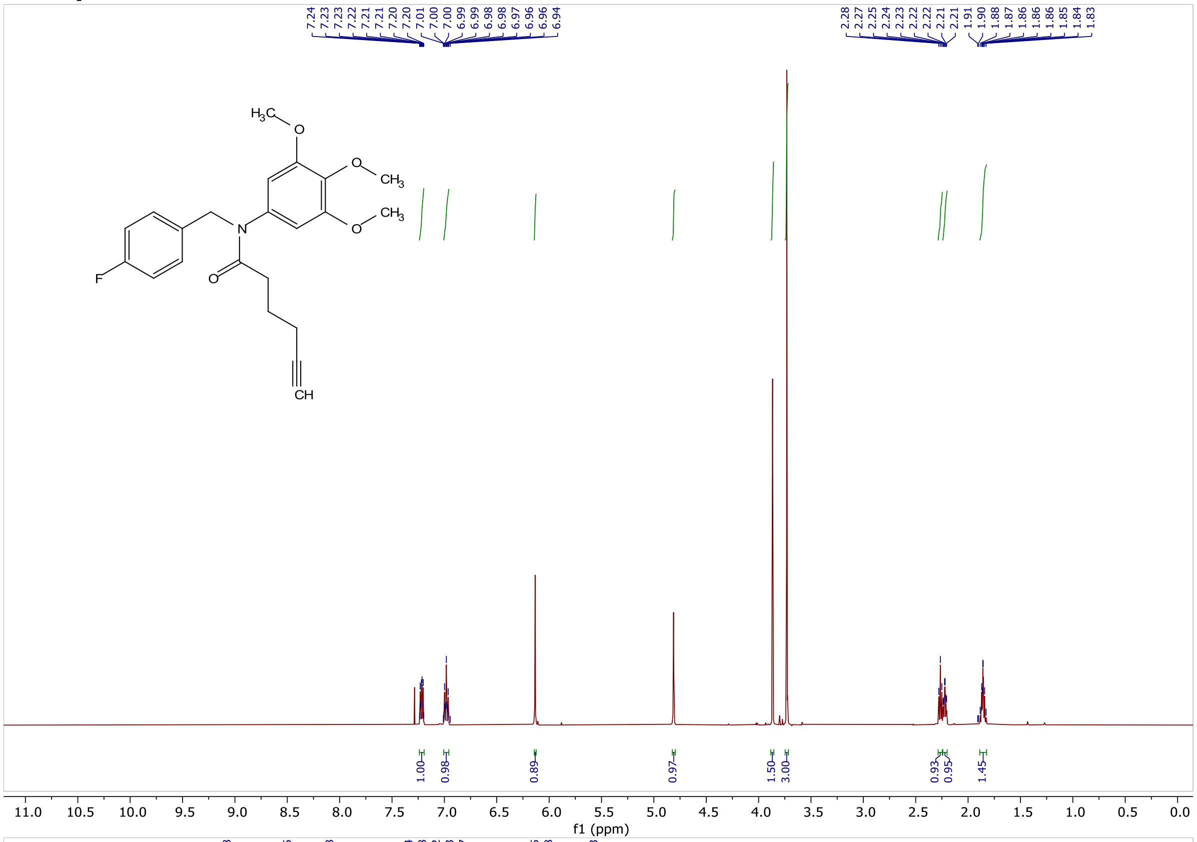

7.24 7.23 7.23 7.22 7.21 7.21 7.20 7.20 7.01 7.00 7.00 6.99 6.99 6.98 6.98 6.97 6.96 6.96 6.94
2.28 2.27 2.25 2.24 2.23 2.22 2.22 2.21 2.21 1.91 1.90 1.88 1.87 1.86 1.86 1.86 1.85 1.84 1.83
H3C O O CH3 CH3 O N F O CH
1.00 0.98 0.89 0.97 1.50 3.00 0.93 0.95 1.45
11.0 10.5 10.0 9.5 9.0 8.5 8.0 7.5 7.0 6.5 6.0 5.5 5.0 4.5 4.0 3.5 3.0 2.5 2.0 1.5 1.0 0.5 0.0
f1 (ppm)

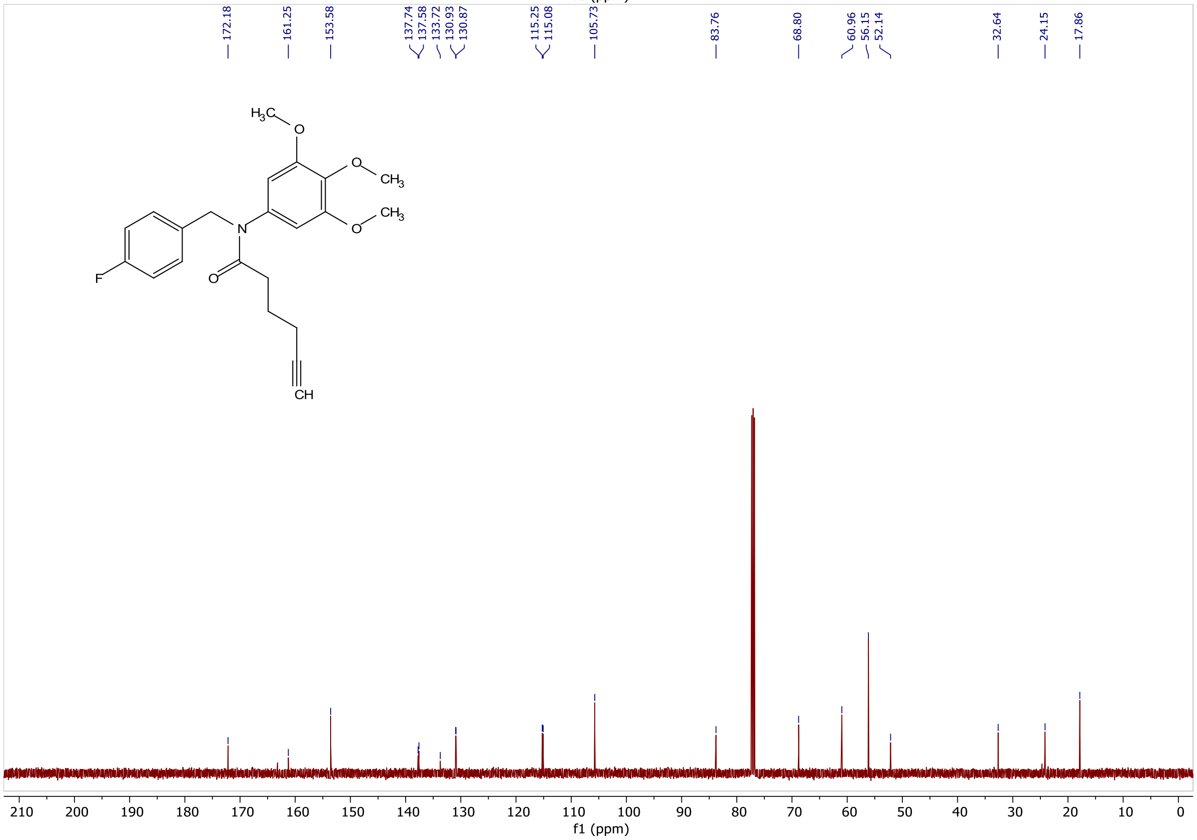

172.18 161.25 153.58 137.74 137.58 133.72 130.93 130.87 115.25 115.08 105.73 83.76 68.80 60.96 56.15 52.14 32.64 24.15 17.86
H3C O O CH3 CH3 O N F O CH
210 200 190 180 170 160 150 140 130 120 110 100 90 80 70 60 50 40 30 20 10 0
f1 (ppm)

PINAD-2

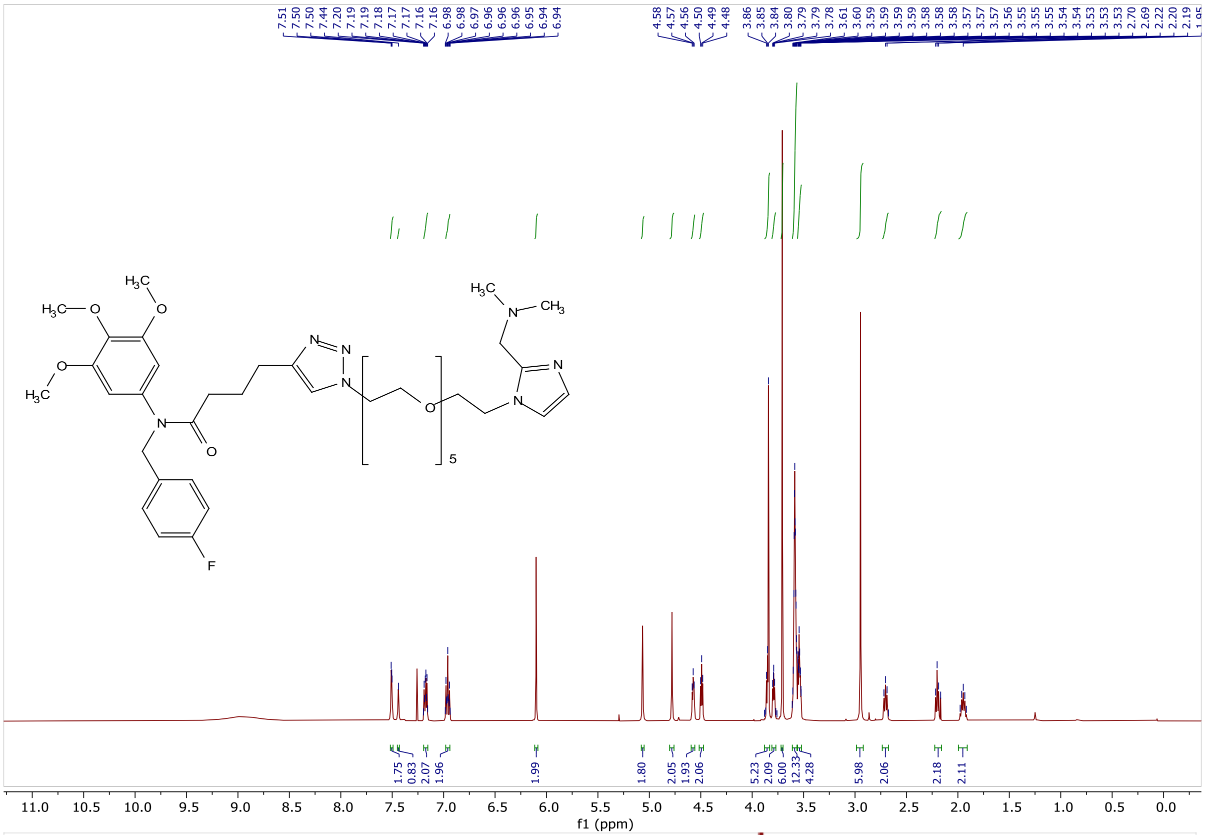

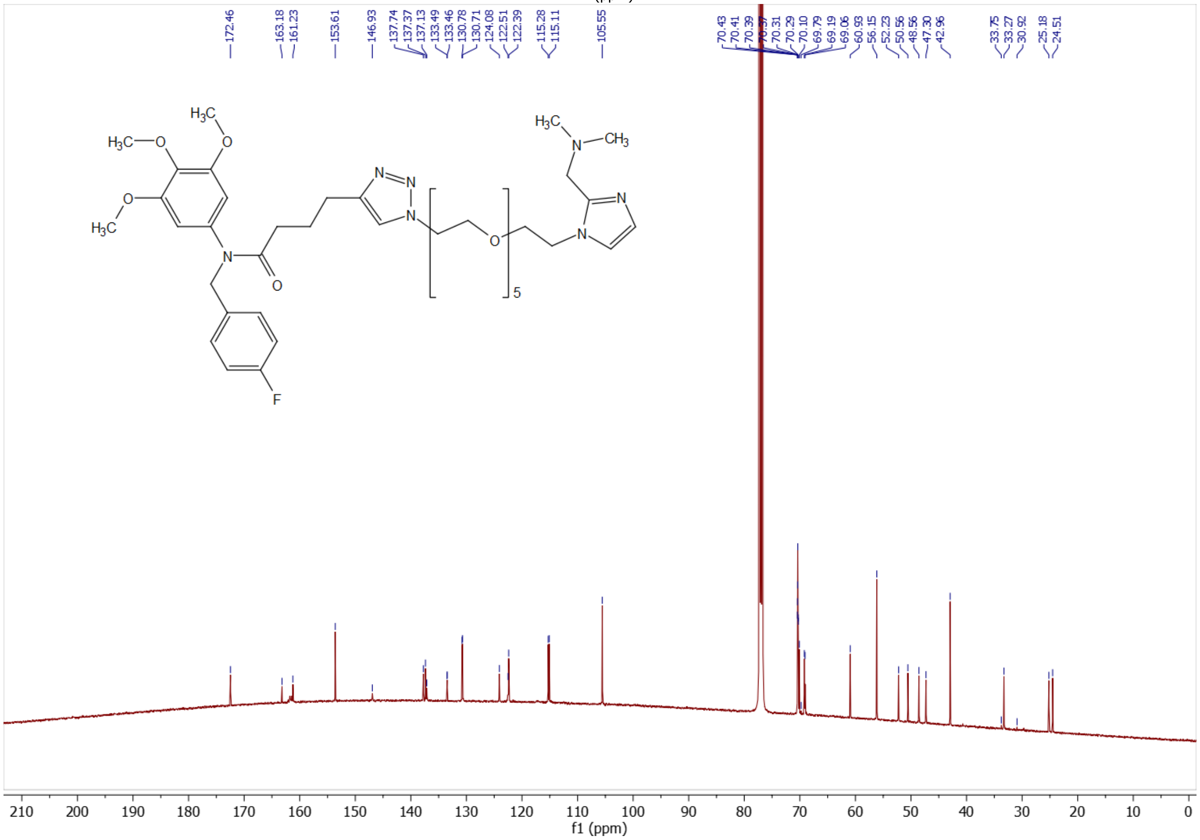

PINAD-1 FAM

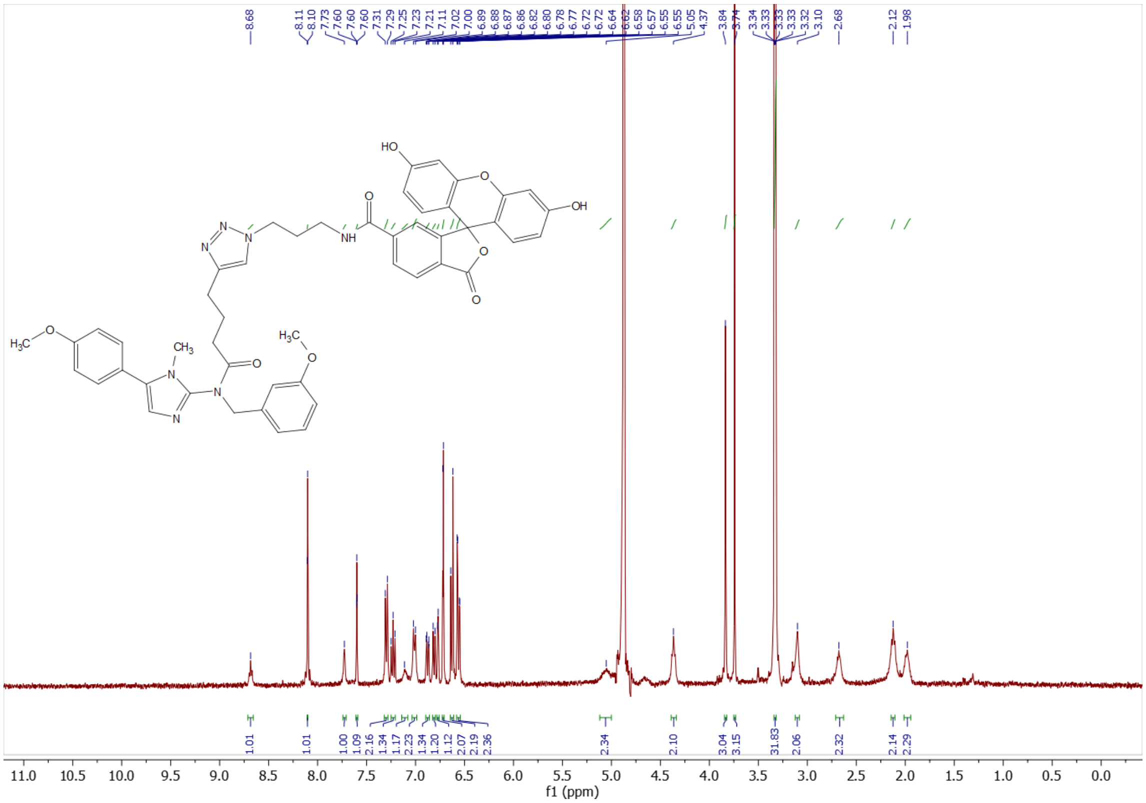

PINAD-2 FAM

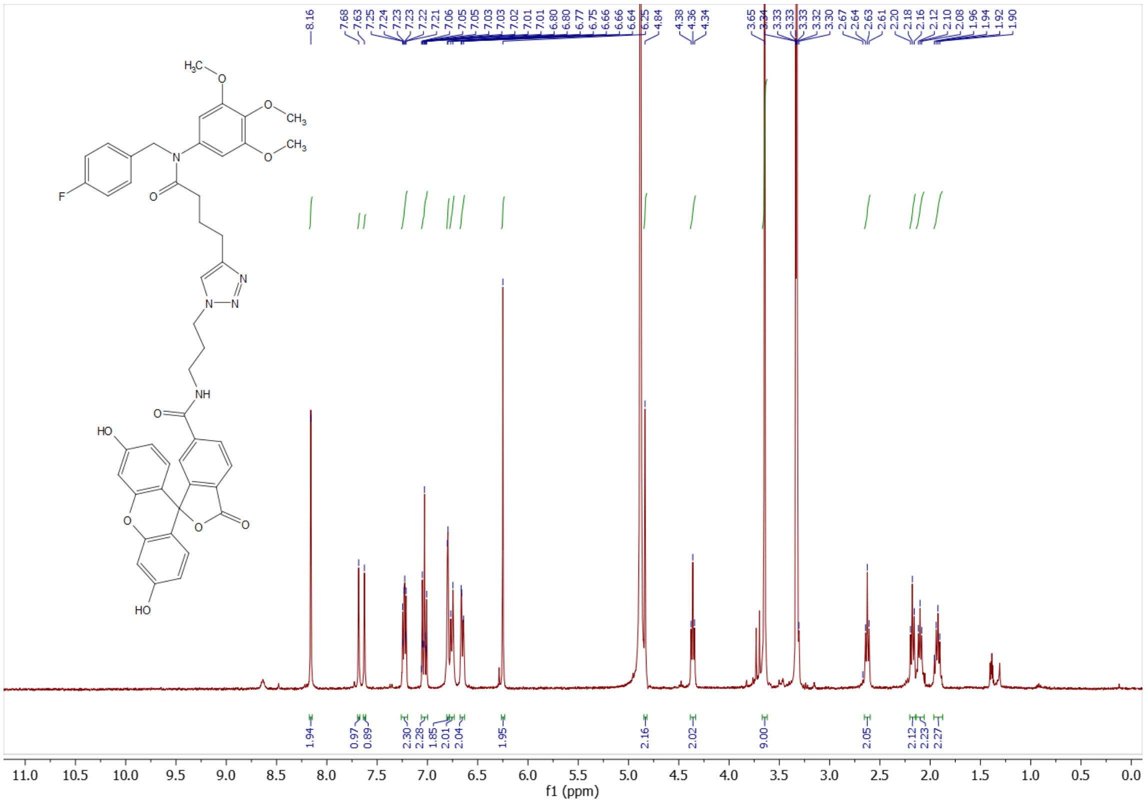

Compound 27

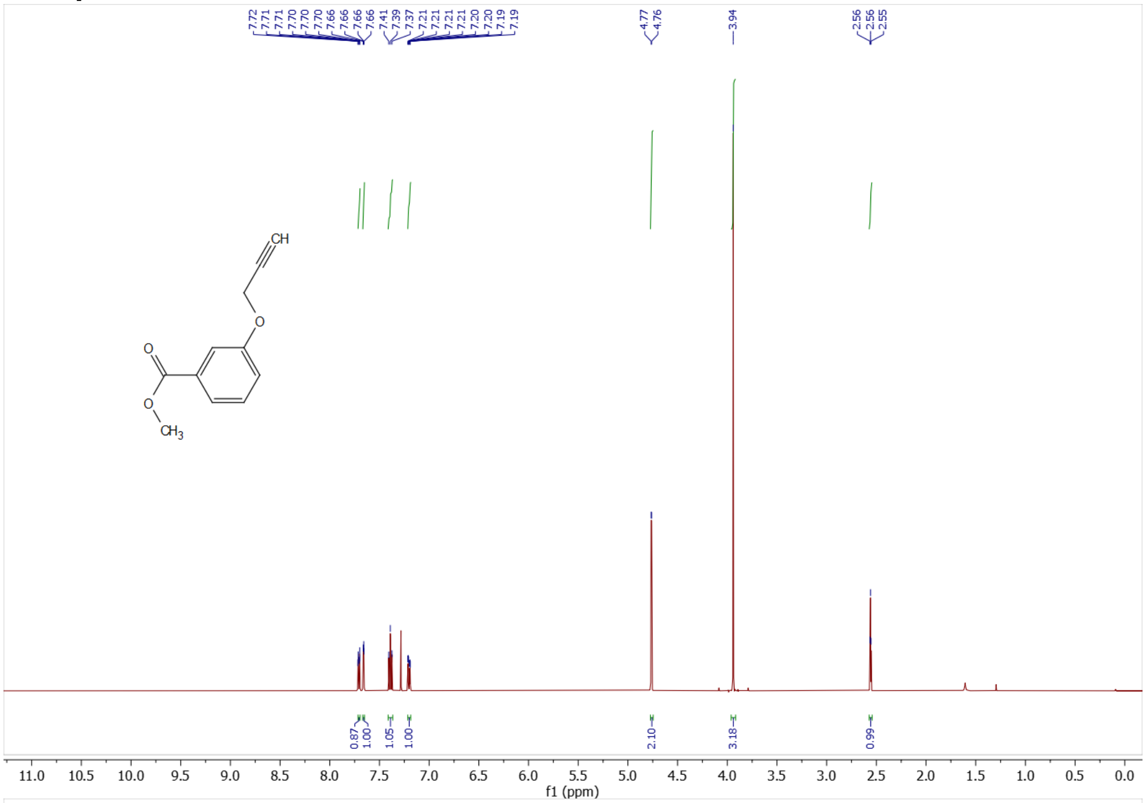

7.72
7.71
7.71
7.70
7.70
7.70
7.66
7.66
7.66
7.66
7.41
7.39
7.37
7.21
7.21
7.21
7.21
7.20
7.20
7.19
7.19
4.77
4.76
3.94
2.56
2.56
2.55
CH
O
O
O
CH3
0.87
1.00
1.05
1.00
2.10
3.18
0.99
11.0 10.5 10.0 9.5 9.0 8.5 8.0 7.5 7.0 6.5 6.0 5.5 5.0 4.5 4.0 3.5 3.0 2.5 2.0 1.5 1.0 0.5 0.0
f1 (ppm)

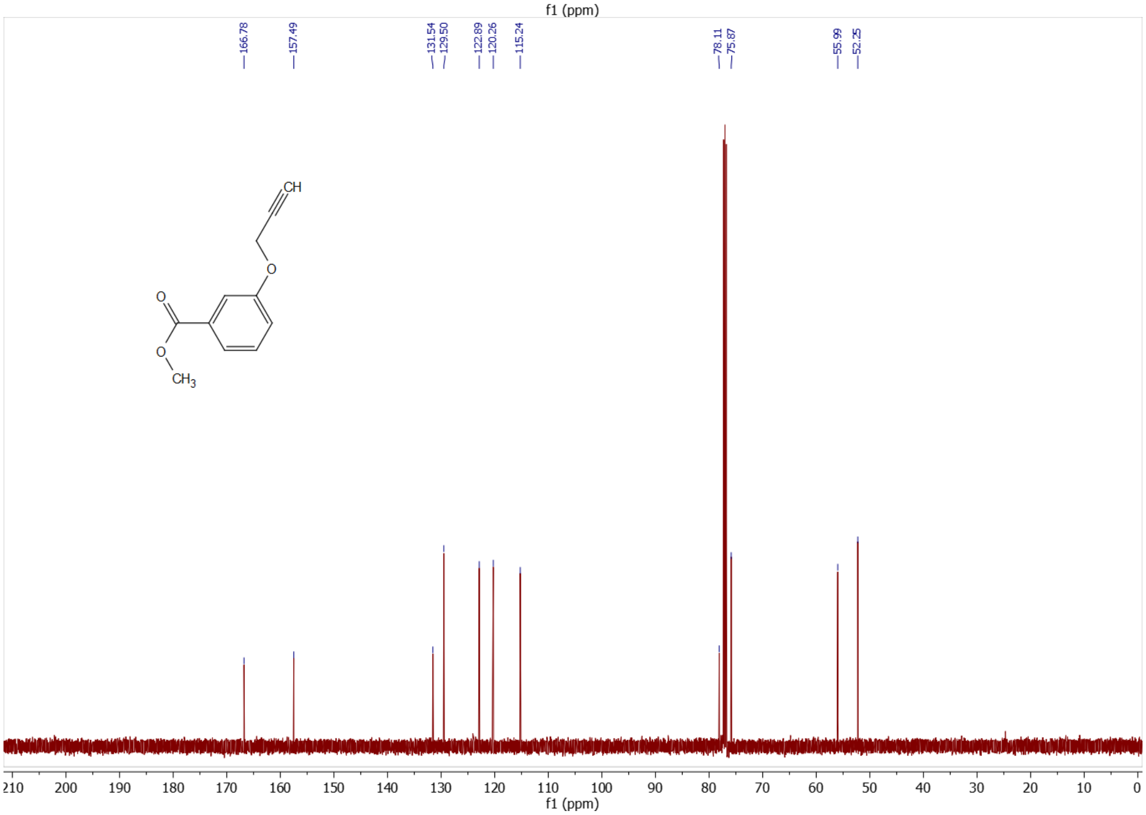

166.78
157.49
131.54
129.50
122.89
120.26
115.24
78.11
75.87
55.99
52.25
CH
O
O
O
CH3
210 200 190 180 170 160 150 140 130 120 110 100 90 80 70 60 50 40 30 20 10 0
f1 (ppm)

Compound 28

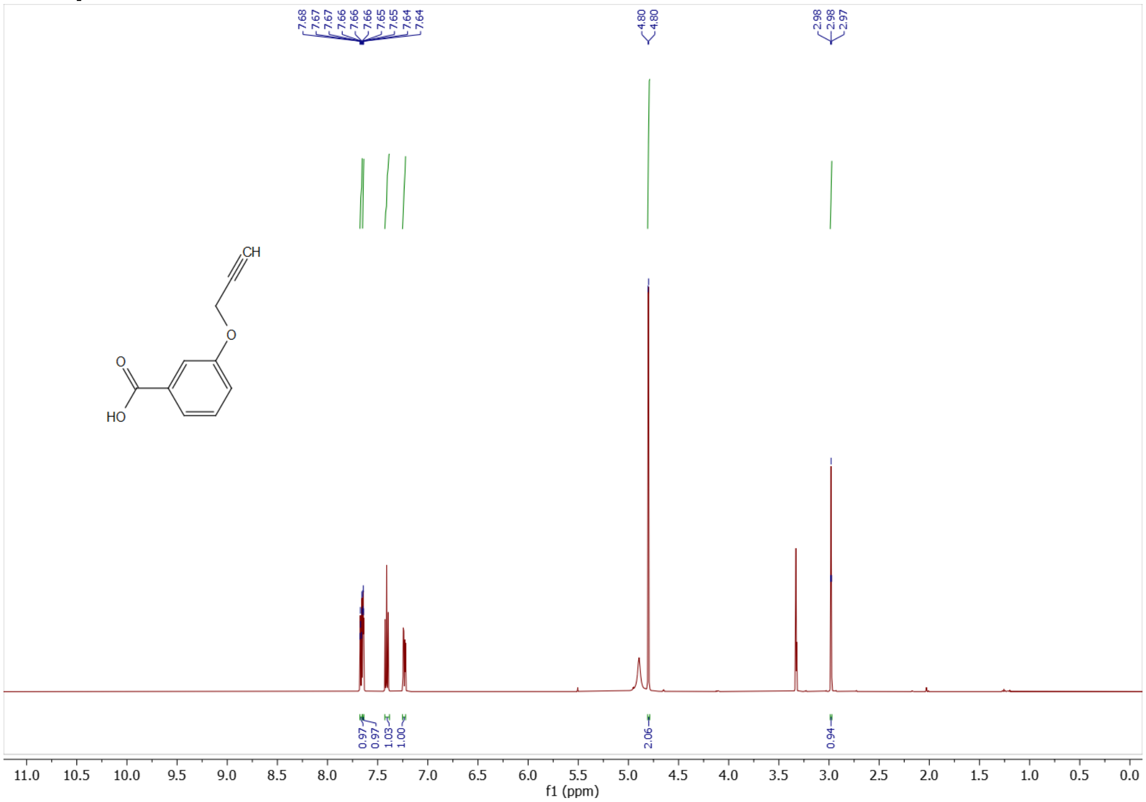

7.68
7.67
7.67
7.66
7.66
7.66
7.65
7.65
7.64
7.64
4.80
4.80
2.98
2.98
2.97
CH
O
O
HO
0.97
0.97
1.03
1.00
2.06
0.94
11.0
10.5
10.0
9.5
9.0
8.5
8.0
7.5
7.0
6.5
6.0
5.5
5.0
4.5
4.0
3.5
3.0
2.5
2.0
1.5
1.0
0.5
0.0
f1 (ppm)

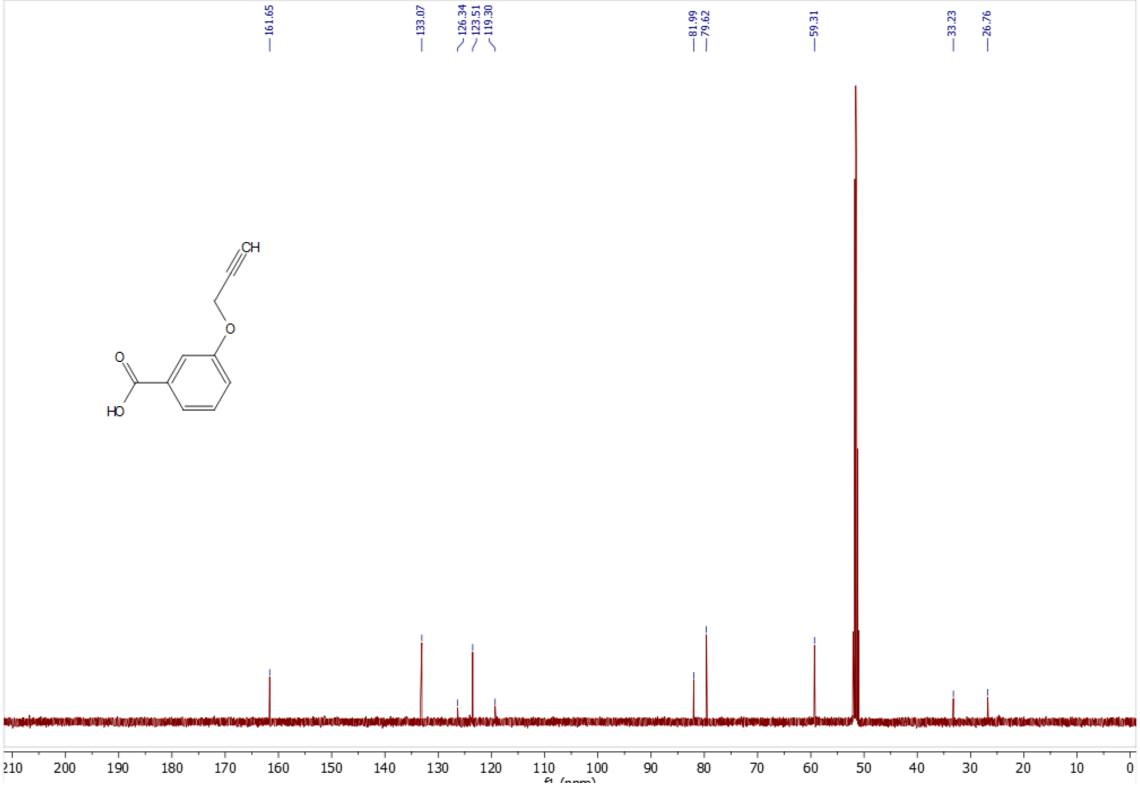

161.65
133.07
126.34
123.51
119.30
81.99
79.62
59.31
33.23
26.76
CH
O
O
HO
210
200
190
180
170
160
150
140
130
120
110
100
90
80
70
60
50
40
30
20
10
0

Compound 29

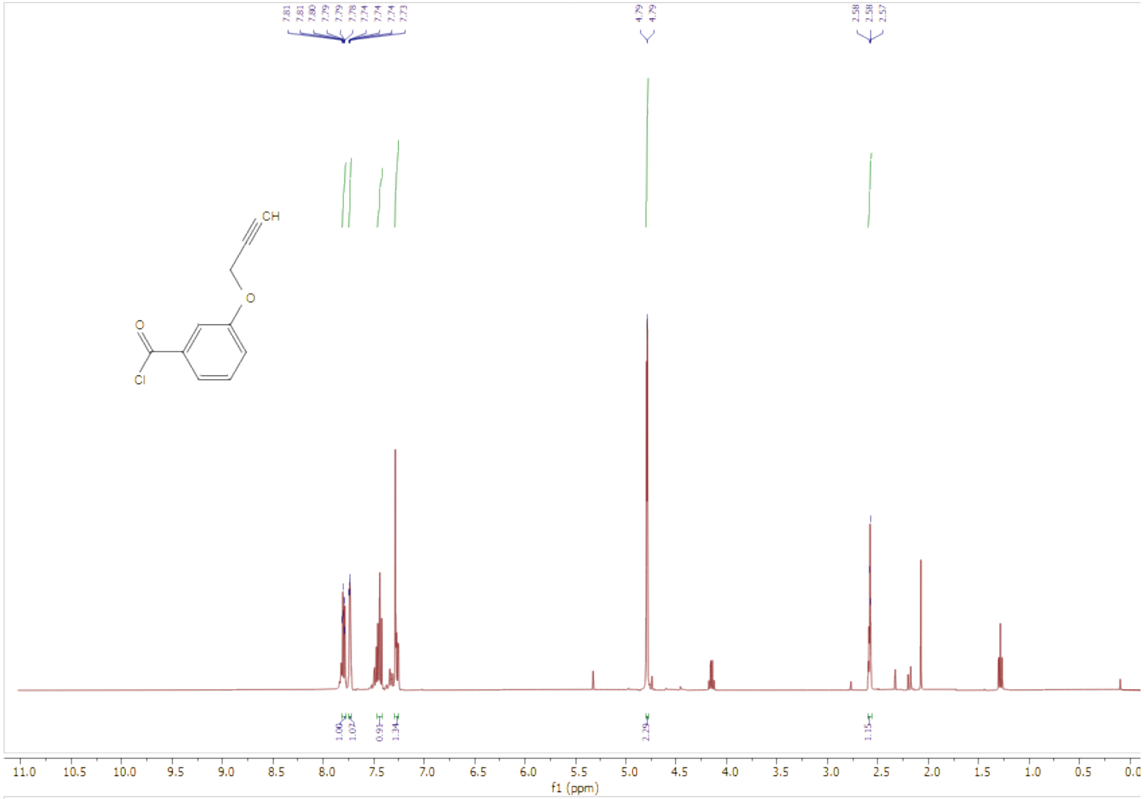

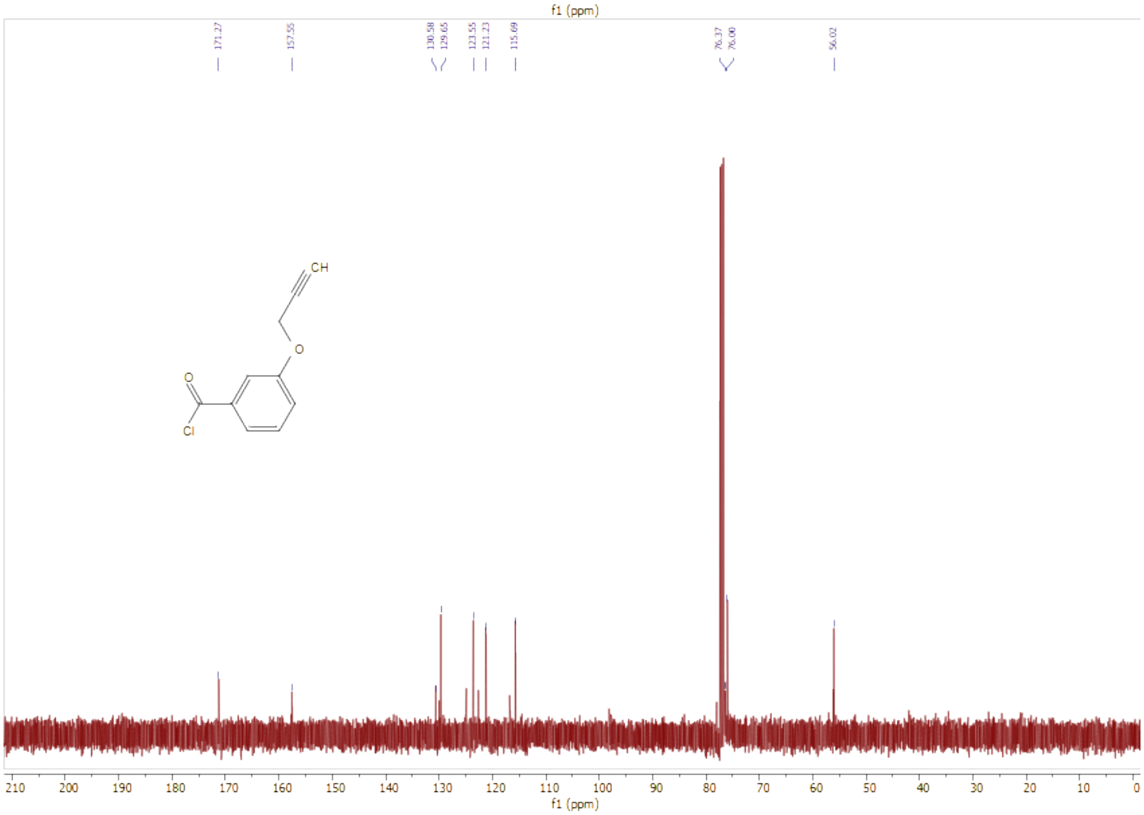

Compound 30

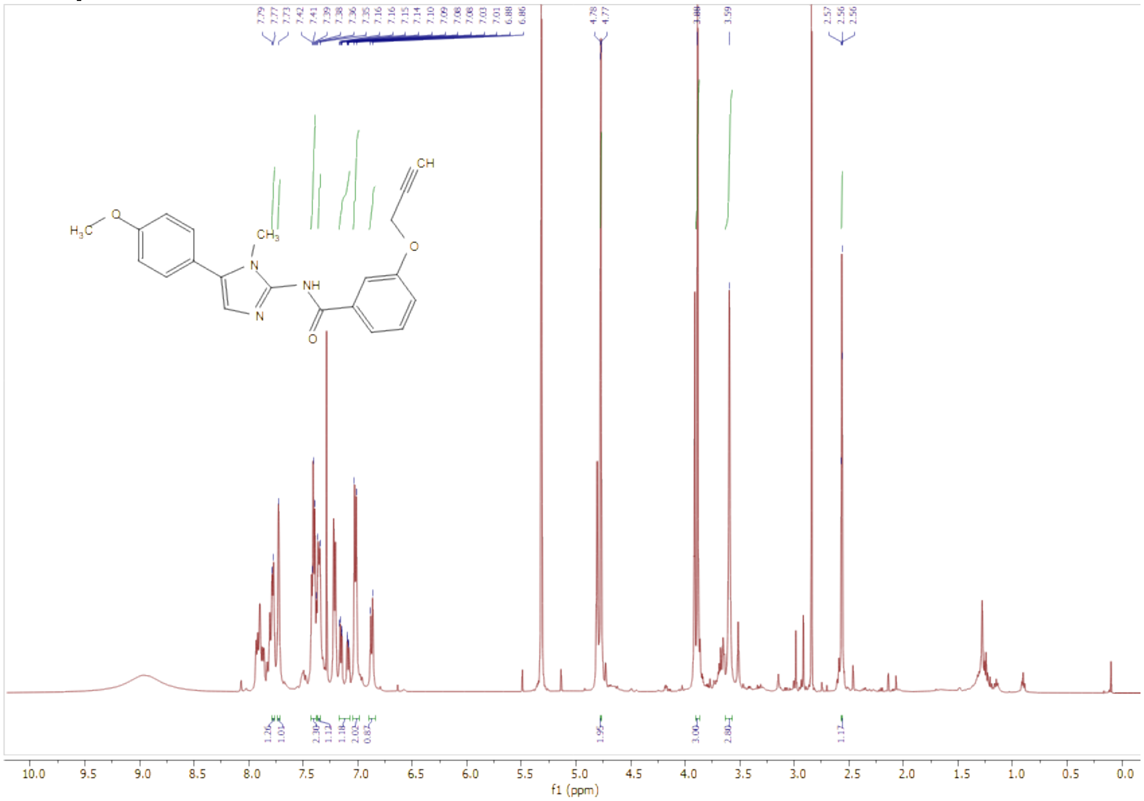

Compound 31

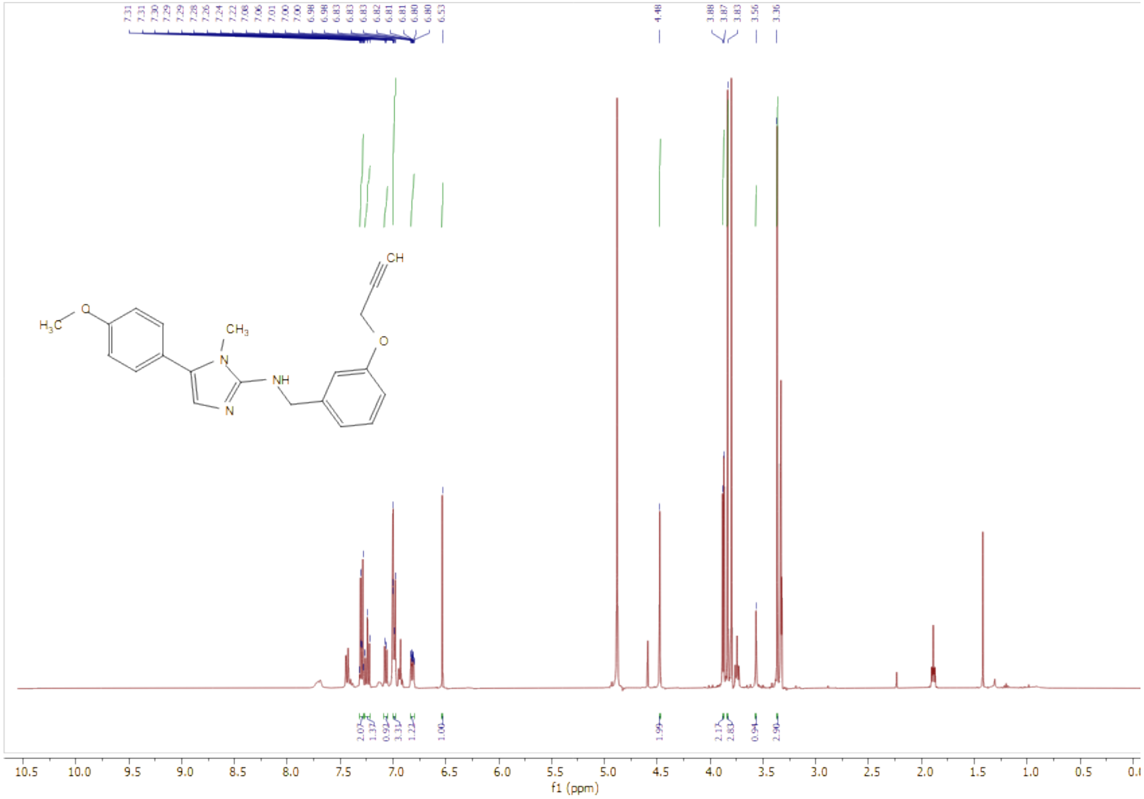

PINAD-1.2

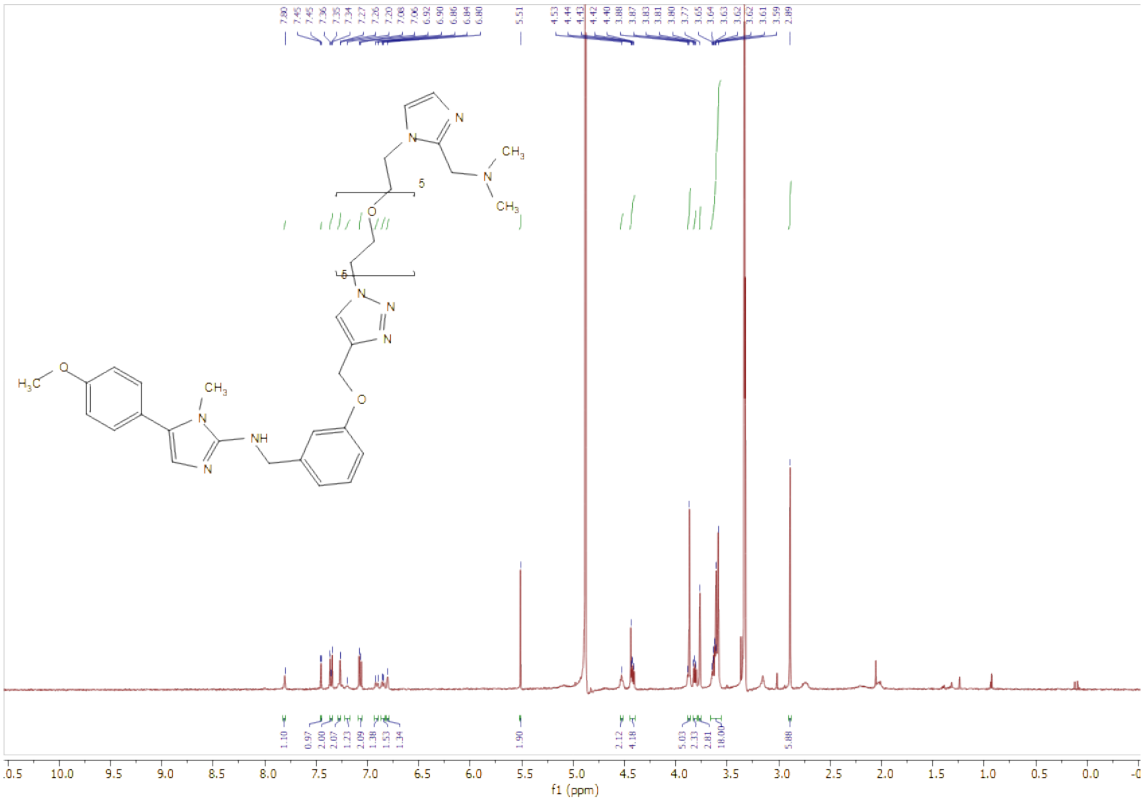

PINAD-1.2 FAM

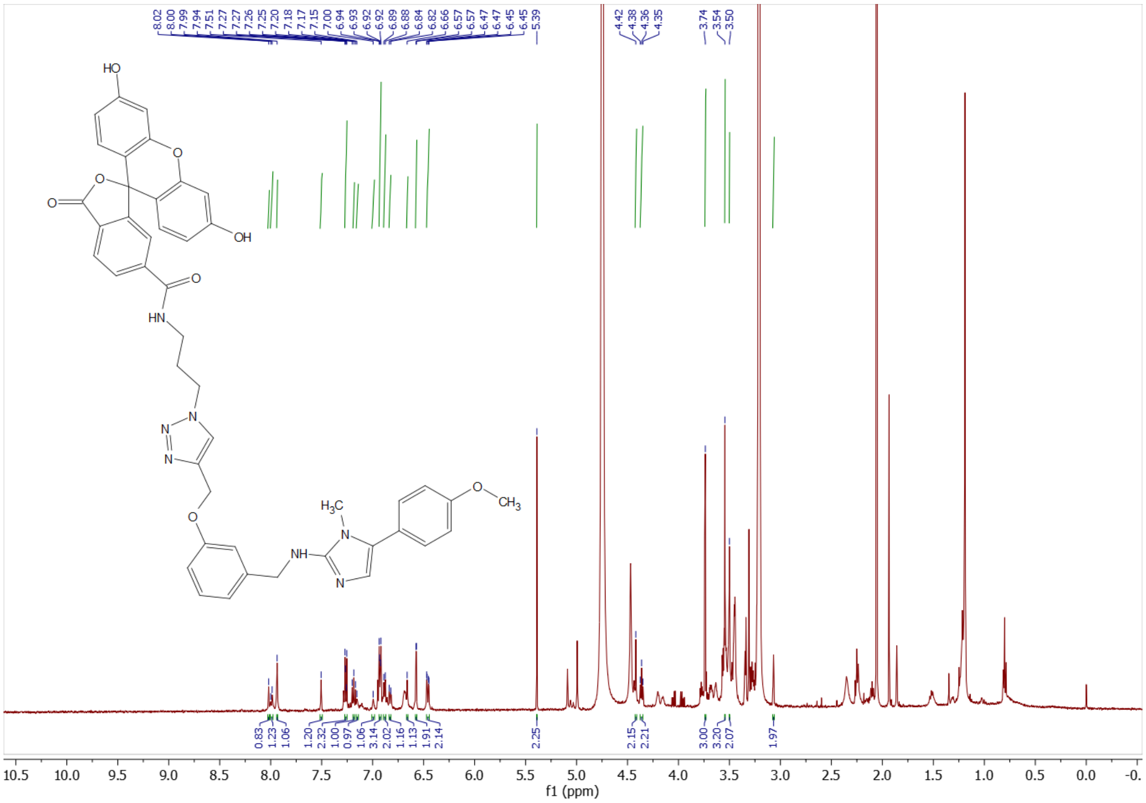

References

[1] S. Mikutis, M. Rebelo, E. Yankova, M. Gu, C. Tang, A. R. Coelho, M. Yang, M. E. Hazemi, M. Pires de Miranda, M. Eleftheriou, M. Robertson, G. S. Vassiliou, D. J. Adams, J. P. Simas, F. Corzana, J. S. Schneekloth, Jr., K. Tzelepis, G. J. L. Bernardes, *ACS Cent. Sci.* **2023**, 9, 892-904.
